# Self-assembled metallasupramolecular cages towards light harvesting systems for oxidative cyclization†

**DOI:** 10.1039/d1sc00097g

**Published:** 2021-03-01

**Authors:** Atul Kumar, Rupak Saha, Partha Sarathi Mukherjee

**Affiliations:** Department of Inorganic and Physical Chemistry, Indian Institute of Science Bangalore 560012 India psm@iisc.ac.in

## Abstract

Designing artificial light harvesting systems with the ability to utilize the output energy for fruitful application in aqueous medium is an intriguing topic for the development of clean and sustainable energy. We report here facile synthesis of three prismatic molecular cages as imminent supramolecular optoelectronic materials *via* two-component coordination-driven self-assembly of a new tetra-imidazole donor (**L**) in combination with 180°/120° di-platinum(ii) acceptors. Self-assembly of 180° *trans*-Pt(ii) acceptors **A1** and **A2** with **L** leads to the formation of cages Pt_4_**L**_2_(**1a**) and Pt_8_**L**_2_(**2a**) respectively, while 120°-Pt(ii) acceptor **A3** with **L** gives the Pt_8_**L**_2_(**3a**) metallacage. PF_6_^−^ analogues (**1b**, **2b** and **3b**) of the metallacages possess a high molar extinction coefficient and large Stokes shift. **1b–3b** are weakly emissive in dilute solution but showed aggregation induced emission (AIE) in a water/MeCN mixture as well as in the solid state. AIE active **2b** and **3b** in aqueous (90% water/MeCN mixture) medium act as donors for fabricating artificial light harvesting systems *via* Förster resonance energy transfer (FRET) with organic dye rhodamine-B (**RhB**) with high energy efficiency and good antenna effect. The metallacages **2b** and **3b** represent an interesting platform to fabricate new generation supramolecular aqueous light harvesting systems with high antenna effect. Finally, the harvested energy of the LHSs (**2b** + **RhB**) and (**3b** + **RhB**) was utilized successfully for efficient visible light induced photo-oxidative cross coupling cyclization of *N*,*N*-dimethylaniline (**4**) with a series of *N*-alkyl/aryl maleimides (**5**) in aqueous acetonitrile with dramatic enhancement in yields compared to the reactions with **RhB** or cages alone.

## Abstract 2

Synthesis of Pt(ii) based metallacages as aggregation induced emissive supramolecular architectures for fabricating artificial light harvesting systems for cross coupling cyclization under visible light is achieved.

## Graphical abstract

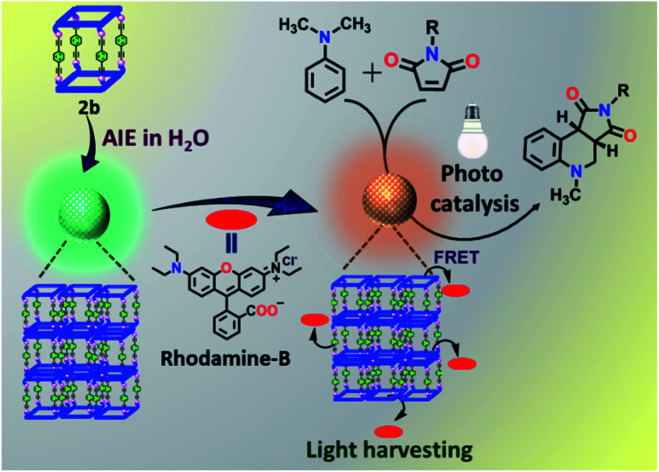

## Introduction

In nature, the photosynthesis process of harvesting solar light and its transformation to chemical energy plays a crucial role in development of life. The process of harvesting light in photosynthesis critically relies on excitation energy migration from the light absorbing pigment ‘chlorophyll’ which is embedded in light harvesting complexes known as antenna proteins to the reaction center ‘carotenoid’ where light energy is utilized for the eventual conversion into chemical energy.^1^ Fluorescence resonance energy transfer (FRET) is a radiation less transition through the dipole–dipole interaction which emerges as one of the most efficient processes of electronic excitation energy transfer within and between molecules for the development of efficient artificial light harvesting systems (LHSs) that mimic the natural photosynthetic process.^2^ In recent years, much attention has been focused by synthetic and material chemists to construct artificial LHSs *via* the FRET process which include conjugated polymers, porphyrin arrays, porous materials, dendrimers, and host–guest assemblies.^3^ Among them supramolecular systems based on non-covalent interactions draw considerable attention in the synthesis of efficient FRET systems as they avoid a multistep synthetic process which is obligatory in the synthesis of scaffold covalent compounds.^4^ Although some successful approaches were adopted for an efficient FRET process in supramolecular architectures based on covalent organic frameworks (COFs)^5^ and metal organic frameworks (MOFs),^6^ such systems are easy to synthesize but their poor solubility and stability in common solvents restrict their processability for further application. Supramolecular coordination complexes (SCCs) formed by coordination driven self-assembly provide an alternative hope in this regard due to their facile one-pot synthesis, good stability, and high solubility in common solvents.^7^ Over the past decade supramolecular materials based on SCCs ranging from 2-D macrocycles to 3-D metallacages have been synthesized which found their widespread use in host–guest chemistry, sensing, catalysis, stabilizing reactive intermediates, *etc.*^8–10^ The FRET process often requires to be performed at very low concentration in order to reduce molecular aggregation. SCCs based on the metal–ligand interaction which is even effective at micromolar to nanomolar concentration are preferred over other host–guest or H-bonded supramolecular complexes in which interactions become ineffective upon dilution due to their low association constant in common organic solvents.^11^ For the synthesis of synthetic LHSs, multiple donor molecules are required for a single acceptor, but use of multiple donors in single molecular assembly can cause fluorescence quenching by aggregation. Therefore, development of aggregation induced emissive fluorophores as donors offers an alternative in constructing efficient LHSs.

Tetraphenylethene (TPE) is weakly emissive in solution but highly emissive in the aggregate state^12^ due to the restriction of phenyl ring rotation.^13,14^ Recently Stang^15^ and other researchers^16^ have reported a series of emissive metallacycles and metallacages as AIE active supramolecular complexes by incorporation of TPE functional units within SSCs *via* coordination driven self-assembly of TPE based donor/(s) and metal-acceptor/(s), which demonstrated their profound use as light emitting and chemical/bio-sensor materials.^17^ AIE active supramolecular chromophores possess distinct features including bright emission with significant quantum yield and extraordinary stability in aqueous media, which make them ideal applicants as light emitting donors for energy transfer with water soluble organic dyes.^2*c*,4,18^ In this regard designing artificial LHSs possessing high energy efficiencies and antenna effect in green aqueous medium with the ability to photo-catalyse chemical transformation under mild conditions by output energy remains a challenging task.

**Scheme 1:**
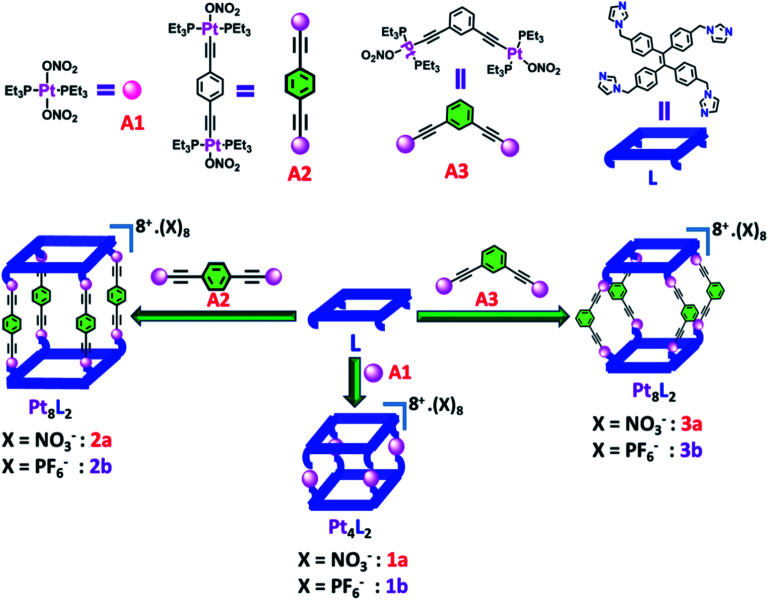
Schematic representation of the formation of Pt(ii) metallacages using a flexible tetra-imidazole donor.

Herein, we report facile synthesis of three prismatic cages *via* co-ordination driven self-assembly of a newly designed donor **L** with 180°/120° *trans*-Pt(ii) acceptors. *Trans*-[Pt(PEt_3_)_2_(ONO_2_)_2_] (**A1**) with **L** leads to the formation of a cage (**1a**) having a molecular composition of **A1**_4_**L**_2_ while 180° based *trans*-Pt(ii) acceptor **A2** and 120° acceptor **A3** with **L** form Pt_8_ cages **2a** (**A2**_4_**L**_2_) and **3a** (**A3**_4_**L**_2_), respectively (Scheme 1). PF_6_^−^ analogues (**1b**, **2b** and **3b**) of the metallacages are highly emissive in the aggregate state in aqueous acetonitrile medium due to the formation of spherical nano-aggregates of the cages having AIE active TPE units. AIE active cages **2b** and **3b** in the aggregate state represent a new platform for fabricating artificial light harvesting systems in the presence of a suitable acceptor dye **RhB**. As cages **2b**/**3b** possess strong absorption in the UV region and emit strongly at lower wavelengths of the visible region, they serve as antenna sources to efficiently transfer the energy from the UV/Visible light to **RhB** by the FRET process and hence stimulate its photocatalytic activity. Furthermore, the LHSs ((**2b** + **RhB**) and (**3b** + **RhB**)) showed dramatic enhancement in visible light mediated photocatalytic activity for the cross coupling cyclization of *N*,*N*-dimethylaniline (**4**) with a series of *N*-alkyl/aryl maleimides (**5**) in aqueous acetonitrile medium (90% water/MeCN mixture) as compared to **RhB**/cages alone. This study provides an efficient and facile approach towards development of aqueous artificial light harvesting materials and use of the harvested energy for visible-light mediated photocatalysis in aqueous acetonitrile medium.

## Results and discussion

### Synthesis and characterization of L and metallacages

Desired ligand **L** was synthesized by allylic coupling of imidazole with 1,1,2,2-tetrakis(4-(bromomethyl)phenyl)ethene **C1** in the presence of a base in 67% yield (Scheme S1†). Ligand **L** was fully characterized by NMR and ESI-MS analyses. Acceptors **A1**, **A2** and **A3** were prepared according to the reported procedures.^19^

**Fig. 1:**
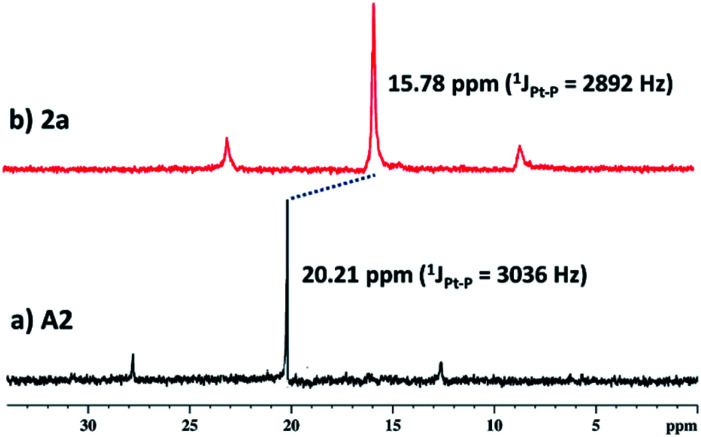
^31^P{^1^H} (162 MHz) NMR comparison of (a) acceptor **A2** and (b) cage **2a** in CD_3_OD.

**Fig. 2:**
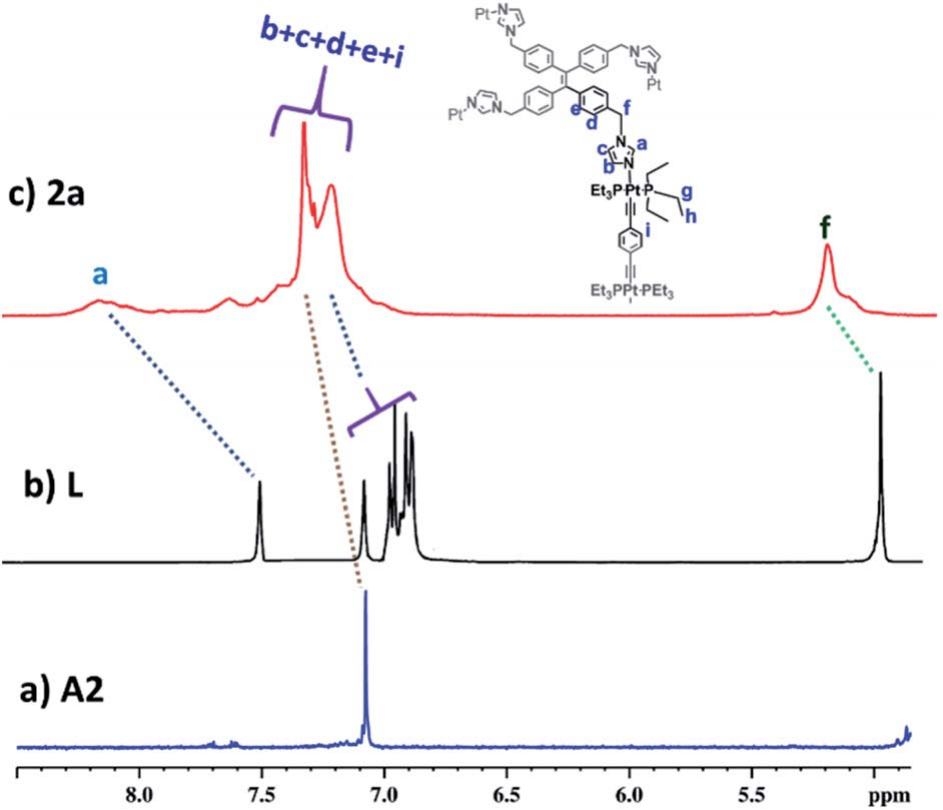
Partial ^1^H (400 MHz) NMR comparison of (a) acceptor **A2**, (b) free ligand **L** and (c) cage **2a** in CD_3_OD.

Self-assembled Pt_4_**L**_2_ molecular cage **1a** and Pt_8_**L**_2_ based cages **2a** and **3a** were prepared by co-ordination driven two-component self-assembly of flexible TPE based tetratopic donor **L** and 180° (for **1a** and **2a**)/120° (for **3a**) based Pt-acceptors. **1a** was prepared by self-assembly of *trans*-[Pt(PEt_3_)_2_(ONO_2_)_2_] (**A1**) with **L** in 2 : 1 molar ratio in methanol (MeOH) with subsequent stirring at 50 °C for 24 h. The resulting clear solution was triturated with diethyl ether to obtain pure self-assembled molecular cage **1a**. Similarly, Pt_8_**L**_2_ based **2a** and **3a** were obtained by self-assembly of donor **L** with 180° Pt-acceptor **A2** or 120° Pt-acceptor **A3** respectively, in 1 : 2 molar ratio in MeOH. **1a–3a** were characterized by ^1^H and ^31^P NMR. The ^31^P{^1^H} NMR spectra (Fig. S6, S10 and S14†) of all the metallacages showed a sharp singlet with associated ^195^Pt satellites (*δ* = 1.12, 15.78 and 15.65 for **1a**, **2a** and **3a** respectively), which suggests the formation of a symmetric single product. The upfield shifts of ∼15.94 ppm, 4.43 ppm (Fig. 1) and 4.30 ppm were observed in the ^31^P{^1^H} NMR of the metallacages **1a**, **2a** and **3a** respectively, with respect to their corresponding Pt-building blocks (**A1**, **A2** and **A3**). Such upfield shifts are due to the donation of electron density by the donor atom to the acceptor upon metal–ligand coordination. The ^1^H NMR spectrum of **2a** reveals an upfield shift with broadness of the imidazole proton (H_a_) as compared to the free ligand due to metal coordination with the formation of a larger assembly. Other aromatic protons of the TPE unit (H_d_ and H_e_), imidazole (H_b_ and H_c_) and Pt-acceptor **A2** (H_i_) merge and give a broad peak at around 7.45–7.56 ppm which is slightly upfield shifted compared to the free ligand. The characteristic CH_2_ peak of **2a** is upfield shifted by Δ*δ* ∼ 0.56 ppm as compared to free **L** (Fig. 2). Similar observation was also noticed on the formation of other two metallacages **1a** and **3a**. The nitrate analogues of the cages **1a**, **2a** and **3a** were converted to corresponding PF_6_^−^ analogues **1b**, **2b** and **3b** respectively, by treating with excess KPF_6_ in methanol. Formation of **1b**, **2b** and **3b** was further investigated by ^1^H and ^31^P NMR, DOSY NMR and ESI-MS. Diffusion-ordered NMR spectroscopy (DOSY) supported the formation of a single product by the appearance of a single band for complexes **1b**, **2b** and **3b** at log *D* = −9.38 (Fig. S17†), −9.78 (Fig. S18†) and −9.81 (Fig. S19†), respectively.

**Fig. 3:**
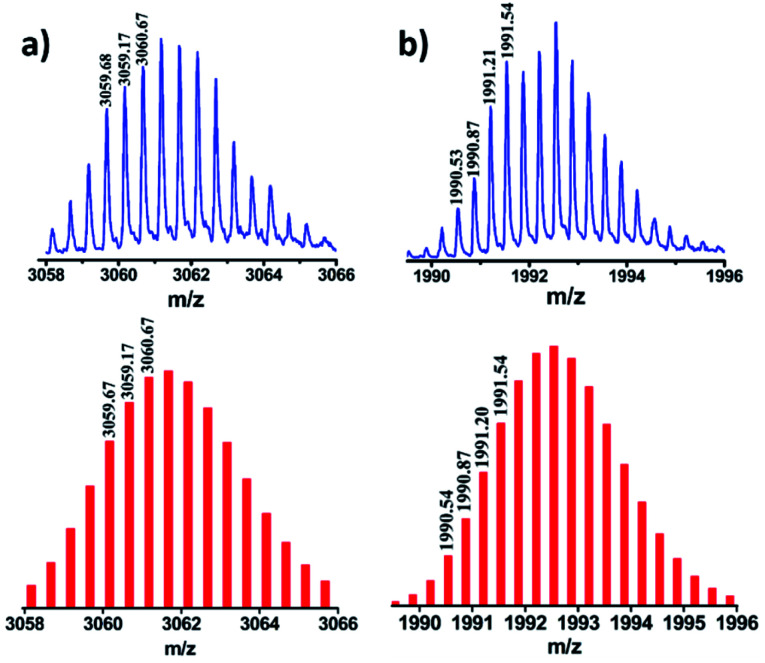
Experimental (top) and theoretical (bottom) isotopic distribution patterns of the peaks corresponding to (a) [**2b**-2PF_6_^−^]^2+^ and (b) [**2b**-3PF_6_^−^]^3+^.

Stoichiometric compositions of **1b**, **2b** and **3b** (PF_6_^−^ analogues) were confirmed by ESI-MS analysis. The mass spectrum for **2b** showed peaks at *m*/*z* = 3061.67, 1992.54, 1458.31 and 1137.49 with isotopic patterns corresponding to [**2b**-2PF_6_]^2+^, [**2b**-3PF_6_]^3+^, [**2b**-4PF_6_]^4+^ and [**2b**-5PF_6_]^5+^ charge fragments, respectively (Fig. 3 and S27†), which suggests the formation of **2b** by [4 + 2] self-assembly of **A2** and **L**. Similarly, the ESI-MS results of **1b** (Fig. S24†) and **3b** (Fig. S29†) were also consistent with the formation of [4 + 2] self-assembled cages of the respective acceptors with donor **L**. In all the cases calculated isotopic distribution patterns matched well with the theoretical patterns (Fig. S23–S29†). DOSY NMR and ESI-MS spectrometry clearly established the formation of molecular assemblies as [4 + 2] quadrangular 3D cages and ruled out the formation of any other possible structure by [2 + 1] self-assembly of the acceptor and donor.

### Computational method

**Fig. 4:**
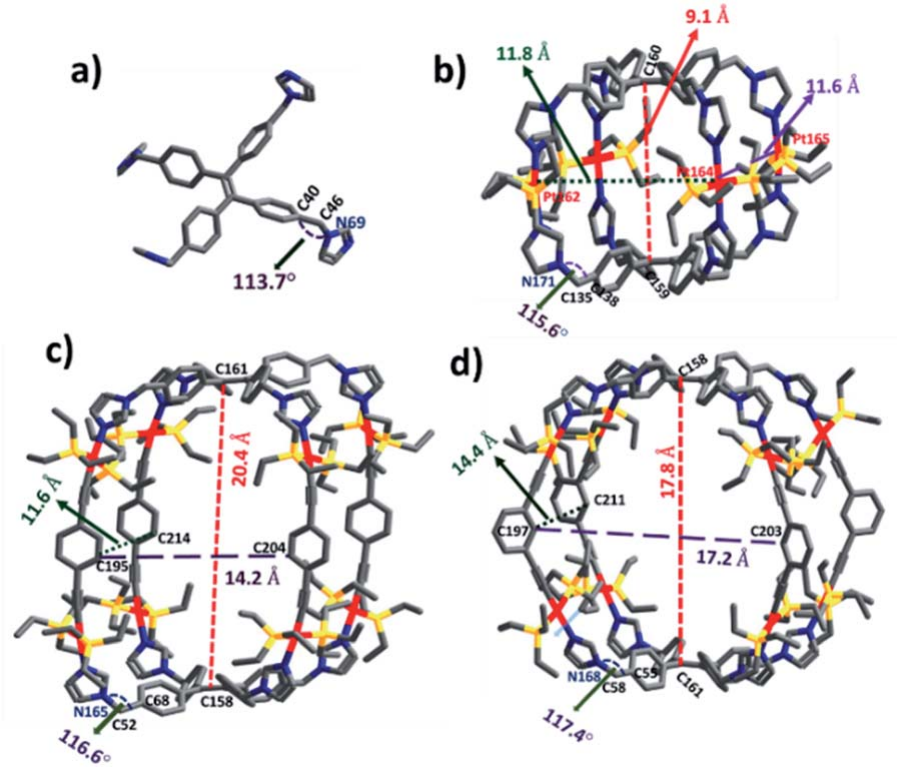
Optimized structures of (a) **L**, (b) **1**, (c) **2** and (d) **3**. Color codes: carbon (grey), nitrogen (blue), phosphorus (yellow), and platinum (red).

To obtain further information on the ground-state structures, **L**, **1**, **2** and **3** were optimized using DFT at the B3LYP/6-31g(d) level for P, C, N, H and B3LYP/LanL2DZ for heavy metal Pt, as implemented in the Gaussian 09 program package.^20^ DFT structures of all the assemblies resemble quadrangular molecular cages which possess different lengths (*l*), breadths (*b*) and heights (*h*). In metallacage **1**, two propeller units of TPE are held by four *trans*-Pt(ii) metal acceptors and separated from each other by 9.1 Å(*C*160 − *C*159), while in **2** and **3**, two units of TPE units are held by 1,4-diethenylbenzene (for **2**) and 1,3-diethenylbenzene (for **3**) and separated from each other by 20.4 Å (*C*161 − *C*158) and 17.8 Å (*C*158 − *C*161), respectively. Formation of molecular structures with two different angles either 180° (**A1** and **A2**) or 120° (**A3**) *trans*-Pt(ii) acceptor becomes feasible due to the flexibility of co- ordinating imidazole because of the CH_2_ group. The angle between phenyl and imidazole in the case of uncoordinated ligand **L** is 113.7° (∠*C*40 − *C*46 − *N*69) which increases upon the formation of metallacages as 115.6° (∠*C*138 − *C*135 − *N*171) for **1**, 116.6° (∠*C*68 − *C*52 − *N*165) for **2** and 117.4° (∠*C*58 − *C*55 − *N*168) for **3**. The overall dimensions (*l* × *b* × *h*) of the cages are ∼(11.8 × 11.6 × 9.1 Å^3^) for **1**, (14.2 × 11.6 × 20.4 Å^3^) for **2**, and (17.2 × 14.4 × 17.8 Å^3^) for **3** (Fig. 4).

### Photophysical properties

UV-Vis and fluorescence spectroscopic studies of all individual compounds **L**, **1b**, **2b** and **3b** were monitored in acetonitrile (MeCN) to probe the absorption and emission patterns (Table S1†). Ligand **L** shows a single absorption band at 280 nm ascribed to the π–π* transition of the TPE moiety. All the metal complexes show dual absorption bands, where the high energy transition corresponds to the π–π* transition similar to **L** while the low energy transition is ascribed to metal to ligand charge transfer (MLCT) and the n–π* transition (Fig. S34†). It is worth noting that bigger metallacages **2b** and **3b** show considerable bathochromic shifts of lower energy bands as compared to smaller system **1b**. Incorporation of 1,4-diethenylbenzene (for **2b**) and 1,3-diethenylbenzene (for **3b**) with additional four Pt(ii) centers provides more electronic communication throughout its structure which causes red shifts of about 20 nm in **2b** (*λ*_max_ = 345 nm) and 15 nm in **3b** (*λ*_max_ = 340 nm) compared to **1b** (*λ*_max_ = 325 nm). In solid state **L** showed a broad absorption maximum at 345 nm which is red shifted by about 65 nm from its solution state. All the metal complexes showed red shifts of around 20 nm in the solid state from their corresponding solution state absorption. This appreciable change in red shifts is common in the solid state (Fig. S35†). **L** showed very low emission with a maximum at 420 nm when excited at 290 nm. Similar large Stokes shifts were also observed for all metallacages (Fig. S34†). **2b** and **3b** showed lower emission intensity with high bathochromic shifts in emission compared to **1b** as they provide better electronic communication within their structures as compared to **1b**.

### AIE behaviour of the metallacages

**Fig. 5:**
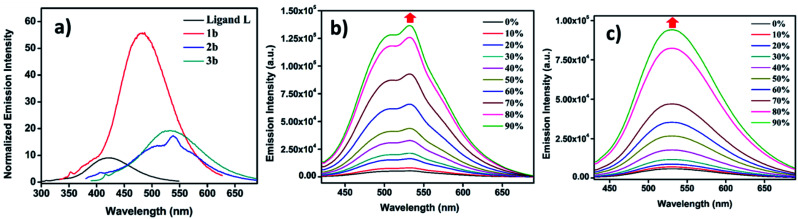
(a) Fluorescence emission of different compounds (*c* = 10^−5^ M) recorded in MeCN. Fluorescence emission with increasing water fraction in MeCN for: (b) **2b** (*λ*_ex_ = 345 nm, *c* = 10^−5^ M) and (c) **3b** (*λ*_ex_ = 340 nm, *c* = 10^−5^ M).

Since ligand **L** contains an AIE active TPE unit, to check the AIE activity of the metallacages (PF_6_^−^ analogues **1b**, **2b** and **3b**) a water/MeCN mixture was selected for supramolecular aggregation. Ligand **L** is weakly emissive in dilute solution in MeCN due to non-radiative decay and shows emission at 420 nm. However, immobilizing weakly emissive **L** in the cage structure due to metal-coordination leads to a six-fold enhancement of fluorescence (Fig. 5a) in **1b**. Metal complexes **2b** and **3b** show only ∼two-fold increase in emission intensity compared to free **L** in MeCN. Due to the greater separation of two TPE units in **2b** and **3b** as compared to **1b** provides more free rotation of the phenyl ring of the TPE units even after co-ordination in the dilute state which leads to small emission enhancement. Upon increasing the water fraction in MeCN, aggregation induced emission enhancement (AIEE) was noticed due to the greater restriction of phenyl ring rotation in the aggregate state. In a 90% water/MeCN mixture maximum emission enhancement was observed for all the cages; **1b** showed 5-fold while **2b** and **3b** showed 25-fold and 16-fold emission intensity enhancement respectively, as compared to their corresponding dilute solutions.

**2b** and **3b** showed greater AIE effects due to more rigidification of TPE units in the aggregate state as compared to **1b**. AIE properties of the metal complexes were well supported by fluorescence quantum yield (*Φ*_F_) measurements (Table S3†). A dilute solution of **L** in MeCN showed *Φ*_F_ = 3.46%, which slightly increased upon metal coordination to 4.63% for **2b** and 6.27% for **3b**, while **1b** showed a considerable increase to 12.45% due to more rigidification of TPE units within the formed supramolecular framework (Fig. S48†). In the aggregate state fluorescence quantum yields were further increased to 21.22% for **1b**, 25.67% for **2b** and 22.34% for **3b** (Fig. S49†). Ligand **L** and all metallacages are highly emissive in the solid state with further red shift in emission as compared to solution where red shifts of about 28 nm for **L**, 30 nm for **1a**, 10 nm for **2b** and 12 nm for **3b** were observed (Fig. S35†). All metal complexes showed further increase in *Φ*_F_ to 24.07%, 29.34% and 26.91% for **1b**, **2b** and **3b** respectively in the solid state (Fig. S50†).

### Morphological characterization in the aggregate state

**Fig. 6:**
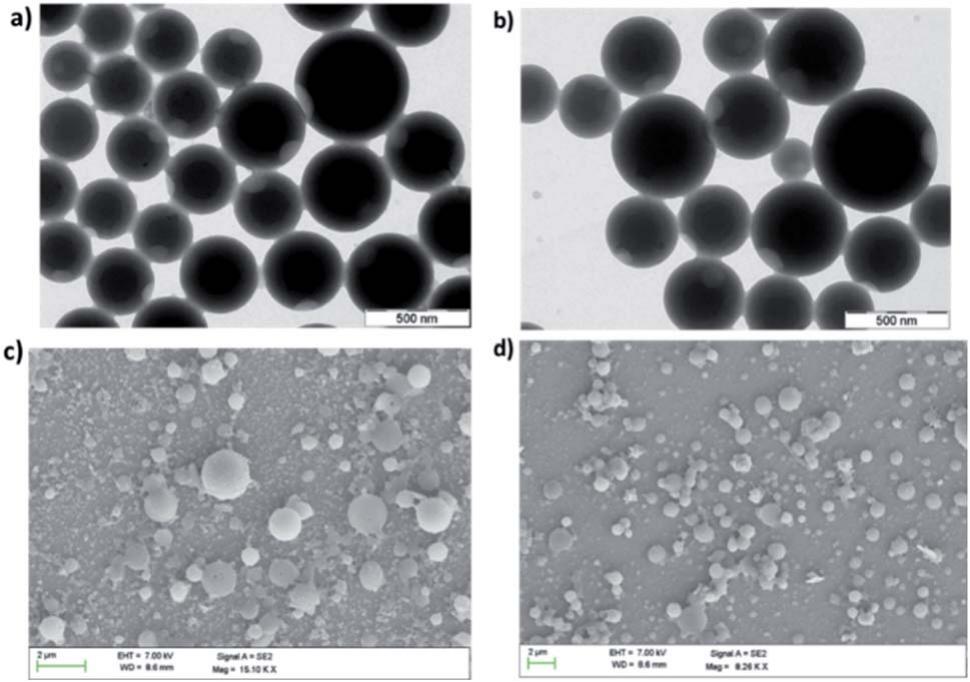
TEM images of aggregates in the 90% H_2_O/MeCN fraction for (a) **2b** and (b) **3b**. SEM images of aggregates in the same medium for (c) **2b** and (d) **3b**.

The change in emission behavior with change in water fraction for all metallacages is due to the formation of aggregates. Therefore, morphologies of the supramolecular aggregates were investigated by scanning electron microscopy (SEM) and transmission electron microscopy (TEM). For SEM analysis 150 μL of 10^−5^ M solution of individual metal complexes **1b**, **2b** and **3b** in a 90% water/MeCN mixture was deposited on silicon wafer followed by drying in a vacuum. SEM analysis of **1b** (Fig. S53†), **2b** (Fig. 6c), and **3b** (Fig. 6d) showed the formation of spherical nanoparticles. For TEM analysis, 5 μL of 10^−5^ M solution in a 90% water/MeCN mixture of respective metal complexes was dropcast on copper grids and dried to afford the final sample. TEM analysis also supports the formation of agglomerated spherical nano-aggregates. The average size of nano-spheres varies from 200 nm to 400 nm for **1b** (Fig. S52†), 350 to 500 nm for **2b** (Fig. 6a) and 300 to 550 nm for **3b** (Fig. 6b). To further support our observation, dynamic light scattering (DLS) analysis was performed on aggregates of all the metallacages in a 90% water/MeCN mixture where we obtained a mean hydrodynamic diameter of around 305 nm for **1b** (Fig. S54a†), 462 nm for **2b** (Fig. S54b†) and 412 nm for **3b** (Fig. S54c†).

### Solvatochromism

Photophysical properties of all the assemblies were investigated in different solvents from non-polar toluene to polar DMSO. The UV-Vis spectra of all the metallacages displayed a slight change in absorption profiles (Fig. S39–S41†). Considerable shifts in fluorescence emission profiles were observed for all metallacages (summarized in Table S2†). In non-polar solvents like toluene and chloroform **1b** showed emission maxima at 460 and 472 nm respectively, which are red shifted in polar solvents like MeCN (*λ*_em_ = 480) and DMF (*λ*_em_ = 479 nm). All metallacages show high emission intensity in non-polar solvents like toluene and chloroform as compared to polar solvents like MeCN or DMF, since non-polar solvents lead to aggregate formation of metallacages due to their poor solvation (Fig. S39–S41†). As all metallacages have AIE active TPE units, their emission got enhanced upon aggregate formation in non-polar solvents.

**Fig. 7:**
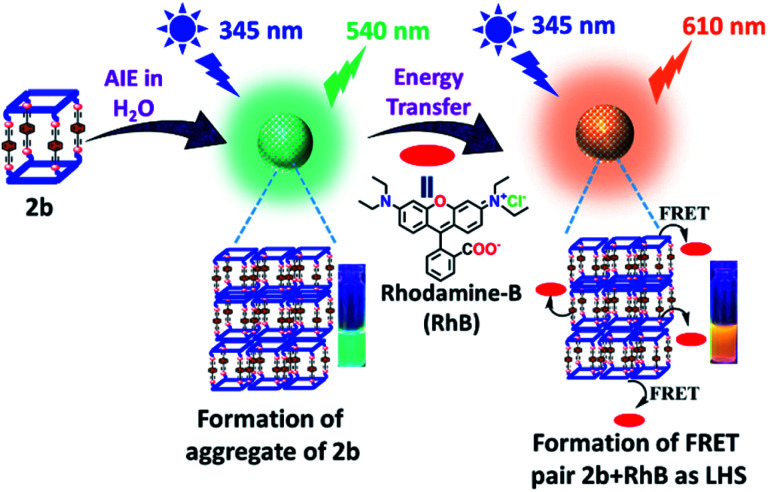
Schematic illustration of AIE and light harvesting function of metallacage **2b**.

### Artificial light harvesting by Förster resonance energy transfer

Metallacage **1b** showed blue emission while **2b** and **3b** showed light-green emission in the aggregate state. The maximum emission intensity was observed in a 90% water/MeCN fraction for all the metallacages. Therefore, the 90% water/MeCN solvent medium was selected for investigation of the energy transfer process as in this medium the cage is stable and doesn't shows any self-quenching over time due to precipitation. Water soluble organic fluorescent dyes are potential candidates for sensors in biological medium or used in energy transfer for artificial light harvesting. For efficient energy transfer by FRET there should be considerable overlap of donor emission and acceptor absorption as the primary requirement.

**Fig. 8:**
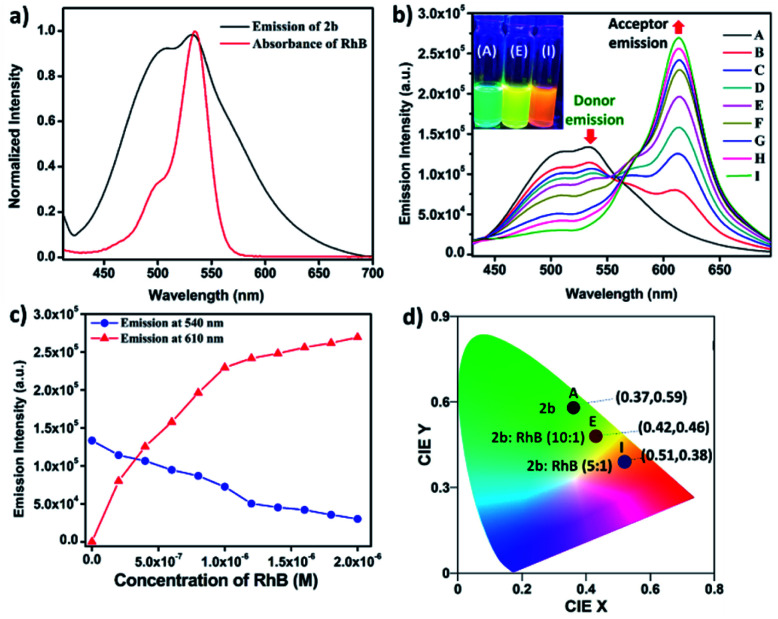
(a) Normalized plot for the emission of **2b** and the absorption of **RhB**. (b) Fluorescence spectra of metallacage **2b** with gradual addition of **RhB** (*λ*_ex_ = 345 nm) in a 90% H_2_O/MeCN mixture. Inset of Fig. 8(b): photograph of **2b** (left), (**2b** + **RhB**, 10 : 1 equiv.) (middle), (**2b** + **RhB**, 5 : 1 equiv.) (right) under UV irradiation at 365 nm. (c) Change in fluorescence emission intensity for **2b** upon gradual addition of **RhB** at 540 nm and 610 nm. (d) The 1931 CIE chromaticity coordinate changes of **2b** (10^−5^ M in a 90% water/MeCN mixture) upon titration with **RhB** (0 to 0.2 equiv.).

Here, we have chosen rhodamine-B (**RhB**) as an acceptor molecule for artificial LHS by the FRET mechanism (Fig. 7). **RhB** absorbs light from 450 nm to 560 nm with the absorbance maximum at 547 nm and emits light at 580 nm in a 90% water/MeCN mixture (Fig. S42†). Cages **2b** (*λ*_em_ = 540 nm)/**3b** (*λ*_em_ = 530 nm) show a considerable overlap of their broad emission with the absorption of **RhB**, while **1b** (*λ*_em_ = 490 nm) doesn't show such considerable overlap (Fig. 8a and S43†). Therefore, **2b** and **3b** become possible donors for energy transfer in artificial LHSs for acceptor **RhB**. The stability of cage **2b** in the presence of **RhB** was first investigated by ^1^H and ^31^P NMR titration. Due to poor ^1^H and ^31^P peak intensity in lower concentration, we checked the stability of cages in the 10^−3^ M concentration range in MeCN-d_3_. Both ^1^H and ^31^P NMR suggest that chemical shifts of **2b** remain unaffected and only an increase in the ^1^H signal of **RhB** was observed upon its gradual addition (up to six equivalents with respect to **2b**) to a cage solution of **2b** (Fig. S20 and S21†).

The energy transfer process was studied by fluorescence titration of **RhB** with the solution of **2b**/**3b**. When 20 μL of 2 × 10^−5^ M (90% water/MeCN mixture) solution of **RhB** was gradually added to 2 mL of 10^−5^ M (90% water/MeCN mixture) cage solution of **2b**, a sharp decrease in emission intensity at 540 nm corresponds to **2b** was observed while fluorescence emission intensity at 610 nm which was further red shifted by 30 nm corresponding to the emission of **RhB** increased upon excitation at 345 nm (Fig. 8b and c). This decrease in emission intensity of **2b** and increase in emission intensity of **RhB** suggest energy transfer from donor **2b** to acceptor **RhB**. This process of energy transfer got saturated upon 200 μL of 2 × 10^−5^ M addition of **RhB** to 2 mL of 10^−5^ M **2b**, suggesting the maximum energy transfer for 5 : 1 ratio for (**2b** + **RhB**). However, under similar conditions a 2 × 10^−6^ M solution of **RhB** in a 90% water–MeCN mixture was found to be non-emissive when excited at 345 nm, which rules out the emission of dye by direct excitation (Fig. S46†). The CIE chromaticity diagram also confirms the change in fluorescence colour from light green (**2b**) [CIE coordinate (0.37 : 0.59)] to yellow (**2b** : **RhB**: 10 : 1) [CIE coordinate (0.42 : 0.46)] to orange-red (**2b** : **RhB**: 5 : 1) [CIE coordinate (0.51 : 0.38)] (Fig. 8d). Similar observation of energy transfer was observed in the case of **3b** with decrease in emission intensity at 530 nm and increase in emission intensity at 615 nm with further red shift by about 35 nm in the emission of **RhB** (Fig. S44†).

The energy transfer process is also evident by the increase in fluorescence quantum yield (*Φ*_F_). When energy transfer is maximum {[cage **2b** or **3b**] = 10^−5^ M and [**RhB**] = 2 × 10^−6^ M} *Φ*_F_ increases to 32.76% for **2b** and 30.54% for **3b**, which were substantially high as compared to *Φ*_F_ of **2b** or **3b** alone in both aggregate (90% water/MeCN) and solid states (Fig. S51†).

**Fig. 9:**
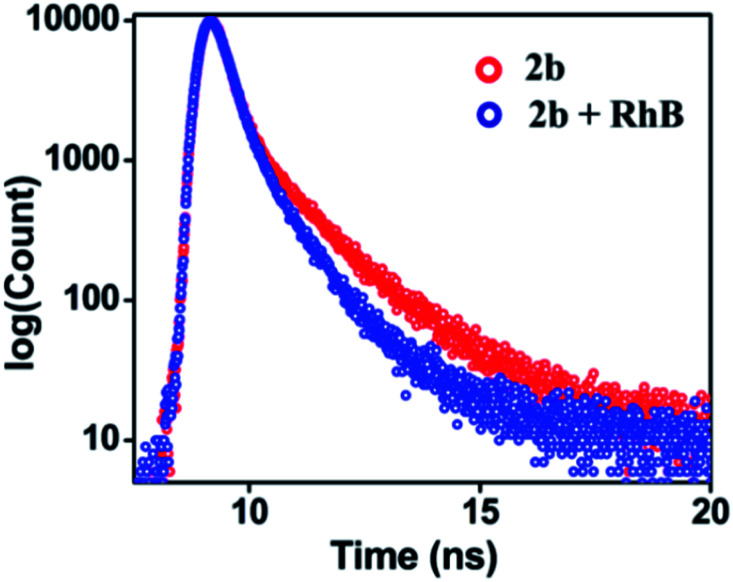
Fluorescence decay profile for **2b** (red, *λ*_ex_ = 340 nm) and (**2b** + **RhB**) (blue, *λ*_ex_ = 340 nm) {[**2b**] = 10^−5^ M, [**RhB**] = 2 × 10^−6^ M} in 90% water/MeCN.

UV-Visible study was performed by gradual addition of a 2 × 10^−5^ M (90% water/MeCN) solution of **RhB** to a 10^−5^ M (90% water/MeCN) cage solution of **2b**. Absorption (*λ*_max_ = 345 nm) corresponding to **2b** remains constant while absorption intensity corresponding to **RhB** at *λ*_max_ = 547 nm increases with gradual addition of **RhB**, which suggests the absence of any ground sate interactions between **2b** and **RhB** molecules (Fig. S45†). The process of FRET from cage **2b** or **3b** to **RhB** was well supported by measurement of fluorescence lifetime of the donor and donor in the presence of the acceptor which is summarized in Table S4.† The fluorescence life-time was measured by the time-correlated single-photon counting technique (TCSPC) which reveals that the fluorescence life time of cage **2b** (*τ*_1_ = 0.97 ns, *τ*_2_ = 3.90 ns, *τ*_av_ = 1.83 ns) or **3b** (*τ*_1_ = 0.80 ns, *τ*_2_ = 3.98 ns, *τ*_av_ = 2.09 ns) was found to be shorter in the presence of dye **RhB** ({*τ*_1_ = 0.74 ns, *τ*_2_ = 2.98 ns, *τ*_av_ = 1.20 ns}) for (**2b** + **RhB**) or {*τ*_1_ = 0.70 ns, *τ*_2_ = 3.21 ns, *τ*_av_ = 1.47 ns} for (**3b** + **RhB**) (Fig. 9 and S47c†). The energy transfer efficiencies (*Φ*_ET_) of fluorescent cages **2b** and **3b** were calculated (eqn S(2)†) as 77% and 58% at a donor/acceptor ratio of 5 : 1 in a 90% H_2_O/MeCN mixture, respectively (Table S5†). In controlled experiments donor–acceptor LHSs (**2b** + **RhB** and **3b** + **RhB**) were excited at acceptor's (**RhB**) absorption maximum at 547 nm (Fig. S55 and S56†) where both LHSs (**2b** + **RhB**) [eqn S(3), Table S6†] and (**3b** + **RhB)** [eqn S(4), Table S7†] show good antenna effect of 21 and 16, respectively.

### Photocatalytic activity and regulation

To explore the use of a light-harvesting system (**2a** + **RhB**) as an efficient energy source, we investigated its photocatalytic activity for the photo-oxidative cross coupling cyclization reaction between *N*,*N*-dimethylaniline (**4**) and *N*-phenyl maleimide (**5a**) in aqueous acetonitrile medium (90% water/MeCN mixture) under visible light to afford 1,2,3,4-tetrahydroquinoline, which is an important product for pharmaceutical industries.^21^ The **LH** complex (**2a** + **RhB**) under optimized conditions showed enhanced photo-oxidative catalytic efficiency with a shorter reaction time in aqueous (90% water/ACN) solution under visible light in comparison to the organic catalyst such as naphthochromenones,^22^ Eosin Y,^23^ metal catalyst,^24^ COF system,^25^ chlorophyll,^26^ and catalyst free system (under UV light/fluorescent lamp)^27^ for the tetrahydroquinoline preparation.

**Fig. 10:**
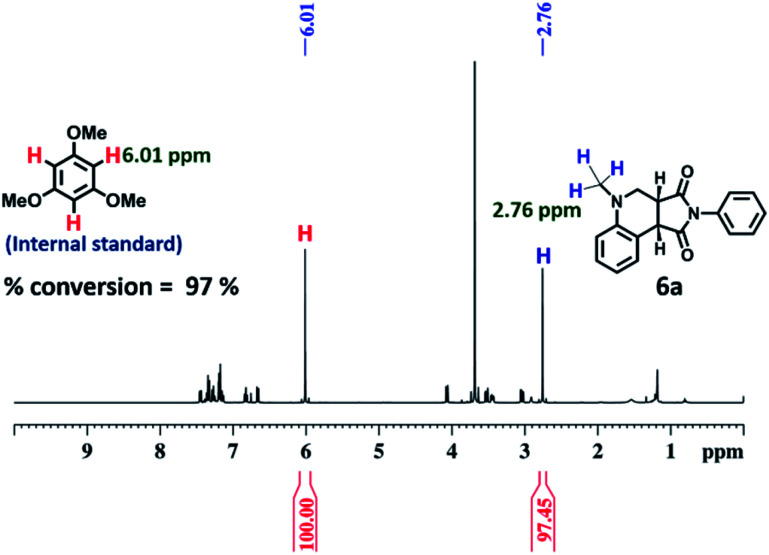
^1^H NMR (400 MHz, CDCl_3_) of the reaction of (**4** + **5a**) using {5 mol% cage **2b** + 1 mol% **RhB**} as the photocatalyst for 12 h under visible light. Percentage conversion of the product **6a** was determined from ^1^H NMR integration based on the starting material with internal standard 1,3,5-trimethoxy benzene.

**Fig. 11:**
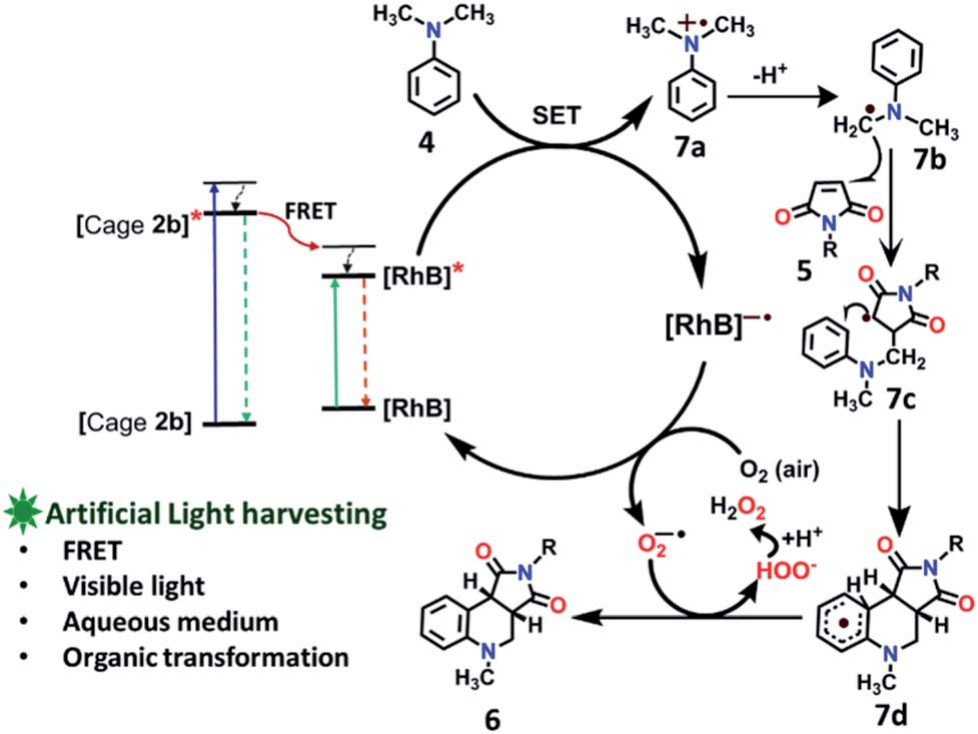
Proposed reaction pathways of photo-oxidative cyclization of *N*,*N*-dimethylaniline **4** and *N*-alkyl/aryl maleimides **5**.

After optimization of the reaction conditions (Table 1, Fig. S58–S67†), we observed that the LH complex (**2a** + **RhB**) {5 mol% **2a**+ 1 mol% **RhB**} readily catalysed the formation of **6a** by the photo-oxidative cyclization reaction of *N*,*N*-dimethylaniline **4** and *N*-phenyl maleimide **5a** in aqueous acetonitrile medium (90% water/MeCN) in an excellent yield of 97% under visible light irradiation for 12 h (Fig. 10). In contrast, **RhB** (1 mol%) alone under similar conditions resulted **6a** in just 5% yield (Fig. S58†). Increasing the amount of **RhB** to 5 mol% marginally increased the product conversion to 13% (Fig. S59†). However, no trace of product (**6a**) formation was observed when the reaction was performed with 5 mol% of cage **2a** alone (Fig. S60†). **RhB** dye has very low absorption at lower wavelengths of visible and UV regions, while cage **2b** possesses strong absorption in the UV region and emits strongly at lower wavelengths of the visible region (*λ*_em_ = 540 nm). Hence, **2b** serves as an antenna source to efficiently transfer energy from UV/Visible light to **RhB** to perform photo-oxidative catalytic reaction. Thus, cage **2b** represents an artificial light harvesting system which mimics the natural photosynthetic process. The above photocatalytic reaction was triggered under very mild conditions by using solar light as an energy source (here we used a white light LED as a solar light simulator). However, very low catalytic activity was observed when the reaction was performed in the dark (Table 1) suggesting the importance of visible light in photo sensitization in the catalytic cycle. As shown in Fig. 11, the reaction proceeds by photosensitizer **RhB** by the single electron transfer (SET) mechanism^21–27^ whose efficiency is substantially improved by energy transfer (by the FRET mechanism) from cage **2b** (Fig. 11). In the above catalytic cycle, molecular oxygen plays a pivotal role in oxidation of the intermediate **7d**. Generation of hydrogen peroxide as a side product during catalysis was detected using the KI/starch indicator (Table S8†). Although the LH system (**3b** + **RhB**) has lower energy transfer efficiency and antenna effect as compared to (**2b** + **RhB**), the product conversion with (**3b** + **RhB**) as a photocatalyst under similar conditions gave an excellent yield of 92% (Fig. S68†).

**Table 1:** Optimization of reaction conditions for the photo-oxidative cyclization reaction^a^

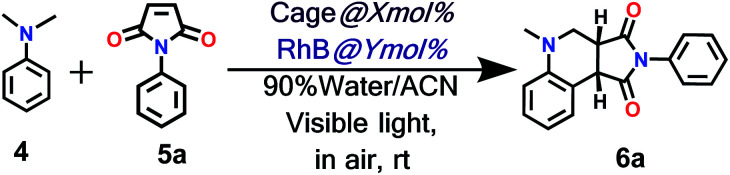					
Entry	Cage **2b** (*X* mol%)	**RhB** (*Y* mol%)	Time (in h)	Light source	% yields of **6a**^b^
1	5	1	4	Visible	37
2	5	1	8	Visible	59
3	5	1	12	Visible	97
4	5	0.5	12	Visible	55
5	5	1.5	12	Visible	98
6	5	1	12	Dark	13
7	None	1	12	Visible	05
8	None	5	12	Visible	16
9	5	None	12	Visible	Trace
10	Ligand **L** (5 mol%)	1	12	Visible	8
11	Cage **3b** (5 mol%)	1	12	Visible	92

a Reaction conditions: *N*,*N*-dimethylaniline **4** (0.04 mmol), *N*-phenyl maleimide **5** (0.04 mmol), water (4.5 mL) + MeCN (0.5 mL), stirring.

b Crude yields determined from ^1^H NMR based on the starting material with internal standard 1,3,5-trimethoxy benzene. Philips 40 W LED bulb (4000 Lm, 6500 K) has been used as a visible light source.

Further, the scope of (**2a** + **RhB**) as a photocatalyst was examined using a series of *N*-alkyl/aryl maleimides (**5**). Substrates with alkyl groups *i.e.*, ethyl (**5b)** and cyclohexyl (**5c**), electron withdrawing aryl groups, *i.e.*, 4-Br-Ph (**5e**), and electron donating aryl groups 4-CH_3_-Ph (**5d**) are consistent with this method and the desired products were obtained in excellent yields of 97–99% (Table 2, Fig. S69–S72†). This artificial light harvesting complex (**2a** + **RhB**) in a 70% water/MeCN mixture is even successful with bulkier *N*-pyrenemalemide (**5f**), which yielded the desired product **6f** in a good yield of 74% (Fig. S73†) easily. Recyclability of the cage **2b** was well examined in the model reaction of **4** and **5a**. After extraction of the reaction mixture with CDCl_3_, the catalysis reaction was further performed with the recovered cage **2b** which catalyses the reaction of **4** and **5a** as fast as the first cycle and doesn't lose its catalytic activity up to four cycles (Fig. S57†).

**Table 2:** Visible light induced photo-catalysis by LHS (**2b** + **RhB**)^a^

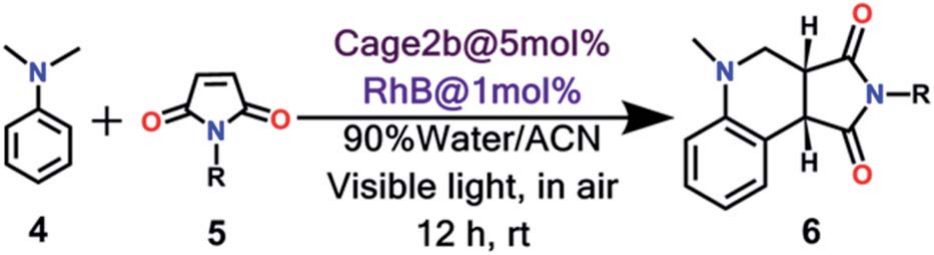			
Entry	R	Product (**6**)	% yields^b^
1	Ph (**5a**)	**6a**	97
2	Ethyl (**5b**)	**6b**	98
3	Cyclohexyl (**5c**)	**6c**	98
4	4-CH3-Ph (**5d**)	**6d**	98
5	4-Br-Ph (**5e**)	**6e**	99
6	1-Pyrene (**5f**)^c^	**6f**	74

a *N*,*N*-Dimethylaniline **4** (0.04 mmol), *N*-alkyl/aryl maleimides **5** (0.04 mmol), catalyst **A** (5 mol%), **RhB** (1 mol%) water (4.5 mL), MeCN (0.5 mL), stirring at rt in open air.

b Crude yields determined from ^1^H NMR based on the starting material with internal standard 1,3,5-trimethoxy benzene.

c Reaction was performed in a 70% water/MeCN mixture (1.5 mL MeCN + 3.5 mL water).

## Conclusions

In summary, we report here facile synthesis of three tetragonal prismatic metallacages *via* two-component self-assembly of a newly designed flexible tetraimidazole donor (**L**) with 180°/120° *trans*-Pt acceptors (**A1–A3**). Due to the presence of the AIE active TPE backbone, the assemblies are highly emissive in both aggregate and solid states, which is supported by increased quantum yield in aggregate and solid states compared to their dilute solution state. Formation of spherical supramolecular nano-aggregates in 90% H_2_O/MeCN was confirmed by SEM, TEM and DLS studies. Moreover, AIE active cages **2b** and **3b** have been utilized for fabricating artificial light harvesting materials in aqueous acetonitrile medium (90% water/MeCN mixture) where they act as donors for energy transfer by the FRET mechanism to organic dye rhodamine-B (**RhB**). The process of energy transfer is well supported by the increase in quantum yield, decrease in lifetime, colour change from light green to red-orange of the cages in the presence of dye **RhB**. The maximum energy transfer efficiencies obtained for (**2b** + **RhB**) and (**3b** + **RhB**) at a donor/acceptor ratio of 5 : 1 in a 90% H_2_O/MeCN mixture are as high as 77% and 58%, respectively. Both the LHSs ((**2b** + **RhB**) and (**3b** + **RhB**)) showed good antenna effect of 21 and 16, respectively. Most importantly, light harvesting materials (**2b** + **RhB**)/(**3b** + **RhB**) have been successfully utilized as visible-light photocatalysts for cross coupling cyclization of *N*,*N*-dimethylaniline and *N*-alkyl/aryl maleimides in aqueous acetonitrile with much enhanced yields compared to similar reactions with dye or cages alone. Overall, this article demonstrates a facile synthetic strategy to obtain AIE active supramolecular architectures for fabricating artificial light harvesting systems in aqueous acetonitrile medium where the output energy is successfully utilized for catalytic chemical transformation for practical application.

## Author contributions

A. K. performed synthesis/characterization of the cages and light harvesting systems. R. S. was involved in data interpretation and catalysis. P. S. M. designed the studies, involved in manuscript preparation. All authors discussed the results and were involved in manuscript writing.

## Conflicts of interest

There are no conflicts to declare.

## Supplementary Material

SC-012-D1SC00097G-s001
