# Supplementary material for: Self-assembled metallasupramolecular cages towards light harvesting systems for oxidative cyclization
Source: Chem Sci. 2021 Mar 1;12(14):5319–29. doi: 10.1039/d1sc00097g (PMC8179592; doi:10.1039/d1sc00097g)
Supplement: SC-012-D1SC00097G-s001 [file SC-012-D1SC00097G-s001.pdf]

Electronic Supplementary Material (ESI) for Chemical Science.
This journal is © The Royal Society of Chemistry 2021

Supporting information

Self-assembled metallasupramolecular cages towards light harvesting systems for oxidative cyclization

Atul Kumar, Rupak Saha, and Partha Sarathi Mukherjee*

Department of Inorganic and Physical Chemistry, Indian Institute of Science, Bangalore 560012, India. E-mail: psm@iisc.ac.in

Experimental section

Materials and methods

The chemicals and solvents used in the present study were purchased from commercial sources and used without further purification. NMR spectra were recorded in a Bruker 400 MHz spectrometer and the chemical shifts (*δ*) in the ^{1}H NMR spectra are reported in ppm relative to tetramethylsilane (Me_4Si) as an internal standard (0.0 ppm) or proton resonance resulting from incomplete deuteration of the solvents $CDCl_3$ (7.26 ppm), CD_3OD (3.30 ppm) and CD_3CN (1.94 ppm). ^{13}C NMR spectra were recorded at 100 MHz, and the chemical shifts (*δ*) are reported in ppm relative to external $CDCl_3$ at 77.8–77.2 ppm. $^{31}P\{^{1}H\}$ NMR spectra were recorded at 162 MHz spectrometer using an external unlocked sample of 85% H_3PO_4 ($\delta = 0$) as reference. Electrospray ionization mass spectrometry (ESI-MS) experiments were carried out in Agilent 6538 Ultra-High Definition (UHD) Accurate Mass Q-TOF spectrometer using standard spectroscopic-grade solvents. IR spectra were recorded with a Bruker ALPHA FTIR spectrometer. Analab μ-ThermoCal10 instrument was used for melting point determination. UV/Vis and fluorescence spectra were recorded with PerkinElmer Lambda-750 and Horiba Jobin Yvon Fluoromax4 spectrophotometers, respectively. The 1931 Commission Internationale de l'e´clairage (CIE) chromaticity coordinates for the corresponding photoluminescence spectra were calculated by "GOCIE" software.[s1] Time resolved fluorescence measurements were carried out on Fluorolog 04 using 325 and 340 nm nano-LED source. The absolute fluorescence quantum yields were measured by Quanta-ϕ Horiba Instrument coupled with Fluorolog spectrophotometer. Transmission Electron Microscopy (TEM) was performed with a JEOL JEM-2100F instrument operating at 200 kV. Dynamic Light Scattering (DLS) measurements were performed with a Zetaseizer instrument ZEN3600 (Malvern, UK) with a 1738 back scattering angle and He-Ne laser (*l*=633 nm). Scanning Electron Microscopy (SEM) image was obtained using Zeiss Ultra-55 SEM instrument with the sample deposited on a silicon wafer. The MeCN/water mixtures with various water fractions were prepared by slowly adding ultrapure water into the MeCN solution of samples. Starting materials for the synthesis of ligand **C1** and *trans*-Pt acceptors **A1**, **A2** and **A3** were prepared according to the reported procedures.[s2] Philips 40W LED bulb (4000 Lm, 6500K) has been used as a white light source for catalysis.

Computational methodology

Full geometry optimizations were performed using the Gaussian 09 package.[s3] The hybrid B3LYP functional was used in all calculations as implemented in the Gaussian 09 package, mixing the exact Hartree−Fock-type exchange with Becke's expression for the exchange functional[s4] that was proposed by Lee, Yang, and Parr for the correlation contribution.[s5] The LanL2DZ basis set was used in the case of the platinum atom and the 6-31g(d) basis set for all other atoms in all calculations. The calculations were carried out methodically with the cationic units in gas phase. Solvent molecules or solvent model or any counter ions were not considered in the calculations.

Synthesis of C1

Compound **C1** was prepared according to the slightly modified reported procedure.[s2a] 1,1,2,2-tetra-p-tolylethene (1 g, 2.57 mmol), N-bromosuccinimide (2.01 g, 11.30 mmol), benzoyl peroxide (31 mg, 0.12 mmol) and CCl_4 (30 mL) were taken in 100 mL round-bottom flask and heated to reflux for 24 h. The reaction mixture was then cooled to ambient temperature and filtered to remove by-product. Crude product was purified by column chromatography using silica 60 – 120 (hexane/ethyl acetate: 70/30) as pale yellow solid. Yield: 1.61 g (89%). m.p. 140 °C. ^{1}H NMR (400 MHz; $CDCl_3$) δ (ppm): 7.13 (d, 8H, *J= 8* Hz), 6.96 (d, 8H, *J= 8* Hz), 4.42 (s, 8H). ^{13}C NMR)100 MHz, $CDCl_3$(δ (ppm): 143.6, 136.7, 132.0, 129.0, 126.5, 33.8. FT-IR (KBr), ν (cm^{-1}): 3925, 3024, 2968, 1915, 1705, 1605, 1509, 1410, 1227, 1097, 1018, 789, 611, 489. ESI MS (m/z): Calcd. For $[M+Na]^+$ 726.83, found 726.84 for $[M+Na]^+$.

Synthesis of L

Imidazole (0.77 g, 11.36 mmol), KOH (0.64 g, 11.36 mmol) and 25 mL of acetonitrile were taken in a 50 mL round bottom flask and the reaction mixture was stirred for 2 h at room temperature. Then compound **C1** (1 g, 1.42 mmol) was added and stirring continued for further 36 h. Subsequently, solvent was evaporated, and water was added to it and extracted with ethyl acetate. Crude product was purified by column chromatography using neutral alumina ($CHCl_3$/methanol: 95/5) as yellow solid. Yield: 0.62 g (67%). m.p. 190 °C. ^{1}H NMR)400 MHz, $CDCl_3$(δ: 7.44)4H, s(, 7.03)4H, s(, 6.84 – 6.93)20H, m(, 5.00)8H, s(. ^{13}C NMR (100 MHz, $CDCl_3$) δ (ppm): 143.4, 140.9, 137.7, 135.1, 132.1, 129.9, 127.3, 119.8, 50.9. FT-IR (KBr), ν (cm^{-1}): 3388, 3112, 3023, 2926, 1923, 1669, 1509, 1282, 1231, 1108, 1076, 1020, 914, 821, 744, 662. ESI MS (m/z): Calcd. For $[M+Na]^+$ 675.30, found, 675.28 for $[M+Na]^+$.

Self-assembly of 1a

Ligand **L** (50.0 mg, 0.07 mmol) and acceptor **A1** (85.1 mg, 0.15 mmol) were mixed in 5 mL methanol followed by stirring at 50 °C for 24 h. Then the reaction mixture was centrifuged and obtained clear solution was concentrated and precipitated with excess diethyl ether to obtain the product as white powder by filtration. Isolated yield: 116.1 mg (86%). m.p. >200 °C. ^{1}H NMR)400 MHz, CD_3OD(δ (ppm): 8.51)8H), 7.30 (12H), 6.86 – 6.96)36H), 5.15)16H), 1.89 (48H), 1.26- 1.29 (72H). ^{31}P{^{1}H} NMR (CD_3OD, 162 MHz) δ (ppm): 1.12 ^{195}Pt satellites, δ (ppm): 10.93 ppm (d, $^1J_{Pt–P}$ = 3924 Hz), -8.51 ppm (d, $^1J_{Pt–P}$ = 3912 Hz).

Self-assembly of 1b

1a (100 mg, 0.03 mmol) was dissolved in 2 mL methanol and excess KF_6 (66.26 mg, 0.36 mmol) was added to it. Then the reaction mixture was stirred at room temperature for 24 h. The obtained precipitate was filtered off and washed several times with methanol and dried in vacuum to get pure metal complex as white powder. Isolated yield: 109 mg (92%). m.p. >200 °C. ^{1}H NMR)400 MHz, CD_3OD(δ (ppm): 8.01)8H), 6.86 – 7.09)48H), 5.01-5.03 (16H), 1.80 (48H), 1.19- 1.24 (72H). ^{31}P{^{1}H} NMR (CD_3OD, 162 MHz) δ (ppm): 1.35 ^{195}Pt satellites, δ (ppm): 11.30 ppm (d, $^1J_{Pt–P}$ = 3980 Hz), -8.34 ppm (d, $^1J_{Pt–P}$ = 3976 Hz). DOSY)400 MHz, CD_3CN) : logD = -9.38 ESI-MS (m/z): 1950.01 [(**1b**) – $2PF_6]^{2+}$, 1252.10 [(**1b**) –

$3PF_6]^{3+}$. FT-IR (KBr), ν (cm^{-1}): 3663, 3594, 3439, 3150, 2977, 2939, 2882, 1671, 1533, 1459, 1417, 1243, 1110, 1037, 848, 557.

Self-assembly of 2a

Ligand **L** (30 mg, 0.045 mmol) and acceptor **A2** (102 mg, 0.09 mmol) were mixed in 5 mL methanol followed by stirring at 50°C for 24 h. Then reaction mixture was centrifuged and obtained clear solution was concentrated and precipitated with excess diethyl ether to obtain the product as white powder by filtration. Isolated yield: 115 mg (87%). m.p. >200 °C. ^{1}H NMR)400 MHz, CD_3OD(δ (ppm): 8.34)8H), 7.45 - 7.56)64H), 5.56 (16H), 2.15 (94H), 1.48 – 1.51 (144H). ^{31}P{^{1}H} NMR (CD_3OD, 162 MHz) δ (ppm): 15.78 ^{195}Pt satellites, δ (ppm): 22.96 ppm (d, $^1J_{Pt–P}$ = 2872 Hz), 8.55 ppm (d, $^1J_{Pt–P}$ = 2892 Hz).

Self-assembly of 2b

2a (100 mg, 0.017 mmol) was dissolved in 2 mL methanol and excess KF_6 (38 mg, 0.21 mmol) was added to it. Then the reaction mixture was stirred at room temperature for 24h. The obtained precipitate was filtered off and washed several times with methanol which was dried in vacuum to get pure metal complex as white powder. m.p. >200 °C. Isolated yield: 104 mg (94%); ^{1}H NMR)400 MHz, CD_3CN(δ (ppm): 7.94)8H), 7.05 - 7.25)64H), 5.16)16H), 1.74 (94H), 1.07 (144H). ^{31}P{^{1}H} NMR (CD_3CN, 162 MHz) δ (ppm): 16.34 ^{195}Pt satellites, δ (ppm): 23.56 ppm (d, $^1J_{Pt–P}$ = 2888 Hz), 9.14 ppm (d, $^1J_{Pt–P}$ = 2880 Hz); δ (ppm) for PF_6^- (septate): -131.34, -135.71, -140.08, -144.44, -148.81, -153.18, -157.50. DOSY (400 MHz, CD_3CN) : logD = -9.78. ESI MS (m/z): 3061.67 [(**2b**) – $2PF_6]^{2+}$, 1992.54 [(**2b**) – $3PF_6]^{3+}$, 1458.31 [(**2b**) – $4PF_6]^{4+}$, 1137.49 [(**2b**) – $5PF_6]^{5+}$. FT-IR (KBr), ν (cm^{-1}): 3646, 3444, 3147, 2973, 2938, 2879, 2123, 1531, 1456, 1414, 1379, 1240, 1109, 1036, 847, 764, 558.

Self-assembly of 3a

Ligand **L** (40 mg, 0.06 mmol) and acceptor **A3** (136 mg, 0.12 mmol) were mixed in 5 mL methanol followed by stirring at 50°C for 24 h. Then reaction mixture was centrifuged and obtained clear solution was concentrated and precipitated with excess diethyl ether to obtain the product as white powder by filtration. Isolated yield: 145 mg (82%). m.p.> 200°C. ^{1}H NMR)400 MHz, CD_3OD(δ (ppm): 8.00)8H), 7.11- 7.21)64H), 5.22 (16H), 1.81 (94H), 1.13- 1.16 (144H). ^{31}P{^{1}H} NMR (CD_3OD, 162 MHz) δ (ppm): 15.65. ^{195}Pt satellites, δ (ppm): 22.81 ppm (d, $^1J_{Pt–P}$ = 2904 Hz), 8.31 ppm (d, $^1J_{Pt–P}$ = 2936 Hz).

Self-assembly of 3b

3a (100 mg, 0.017 mmol) was dissolved in 2 mL methanol and excess KF_6 (38 mg, 0.21 mmol) was added to it. Then the reaction mixture was stirred at room temperature for 24 h. The obtained precipitate was filtered off and washed several times with methanol which was dried in vacuum to get pure metal complex as white powder. m.p.> 200°C. Isolated yield: 100 mg (90%); ^{1}H NMR)400 MHz, CD_3CN(δ (ppm): 8.23)8H), 7.34 - 7.73)64H), 5.45 (16H), 1.74 (94H), 1.07 (144H). ^{31}P{^{1}H} NMR (CD_3CN, 162 MHz) δ (ppm): 16.42 ^{195}Pt satellites, δ (ppm): 23.58 ppm (d, $^1J_{Pt–P}$ = 2864 Hz), 9.09 ppm (d, $^1J_{Pt–P}$ = 2932 Hz); δ (ppm) for PF_6^- (septate): -131.34, -135.71, -140.08, -144.44, -148.81, -153.18, -157.50. DOSY (400

MHz, CD_3CN) : logD = -9.81. ESI MS (m/z): 1992.54 [(**3b**) – $3PF_6]^{3+}$, 1137.49 [(**3b**) – $5PF_6]^{5+}$, 923.80 [(**3b**) – $6PF_6]^{6+}$. FT-IR (KBr), ν (cm^{-1}): 3508, 3133, 2969, 2939, 2881, 2126, 1586, 1530, 1457, 1417, 1272, 1158, 1032, 767, 638, 537, 517.

Reaction scheme

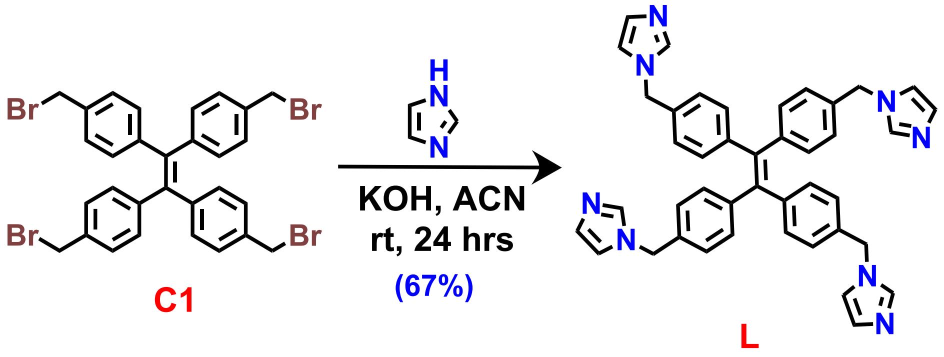

Scheme S1 Reaction scheme for synthesis of **L**.

Spectroscopic characterization

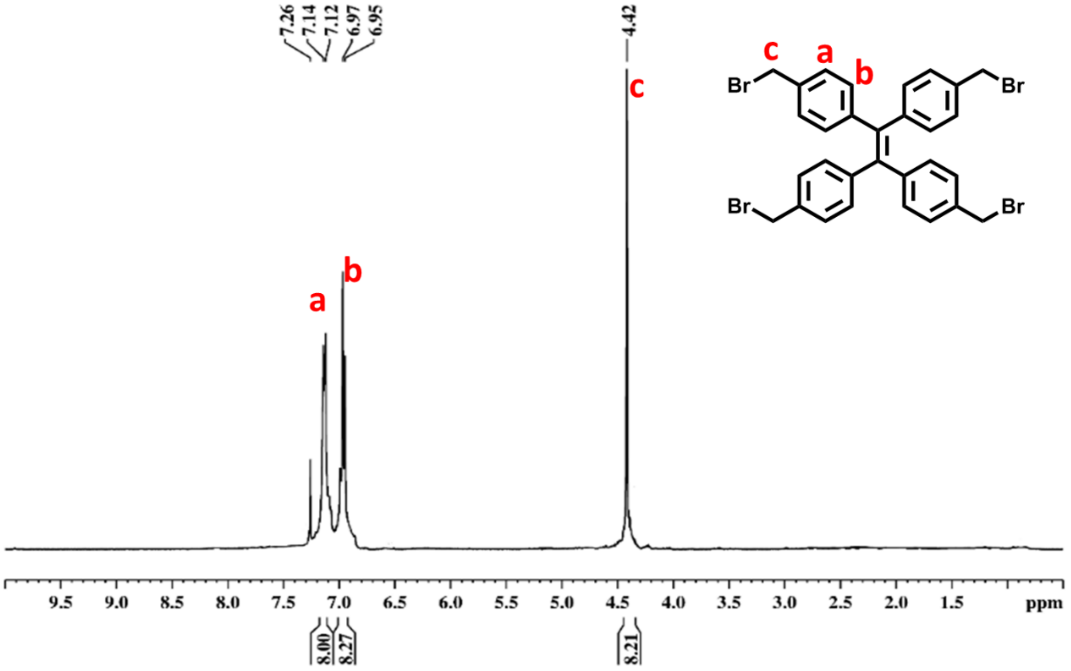

Fig. S1 ^1H NMR ($CDCl_3$, 400 MHz) of **C1**.

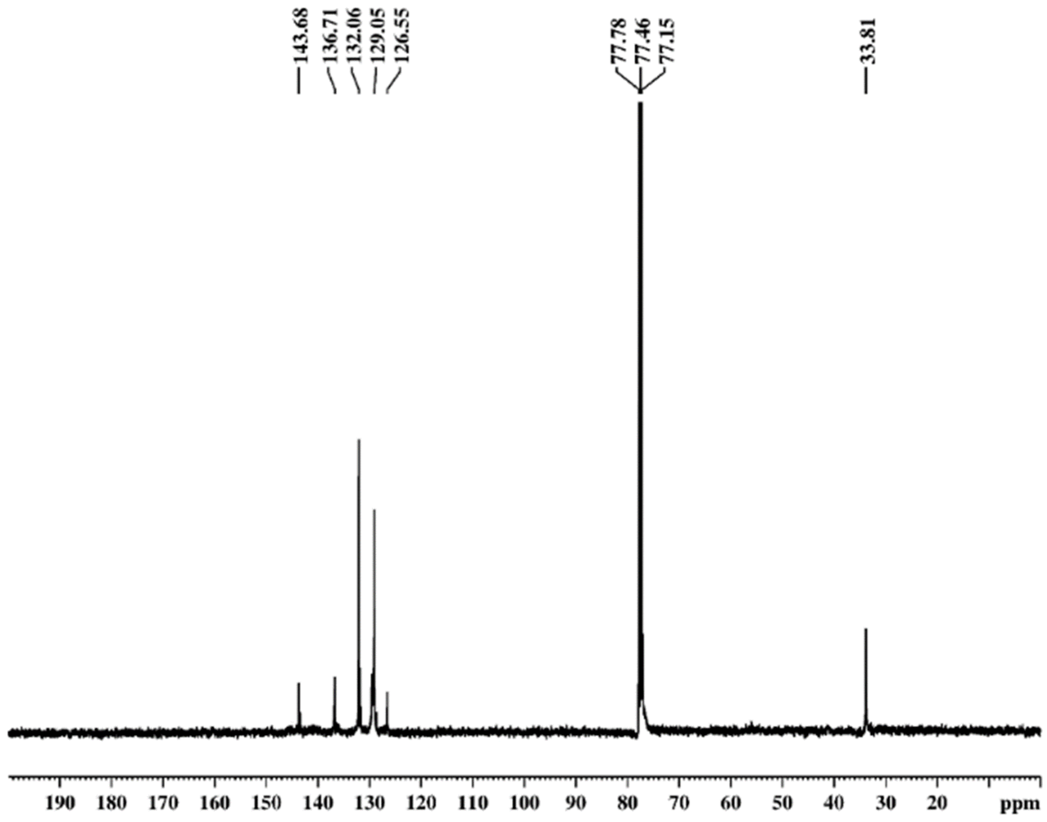

Fig. S2 ^{13}C NMR ($CDCl_3$, 100 MHz) of **C1**.

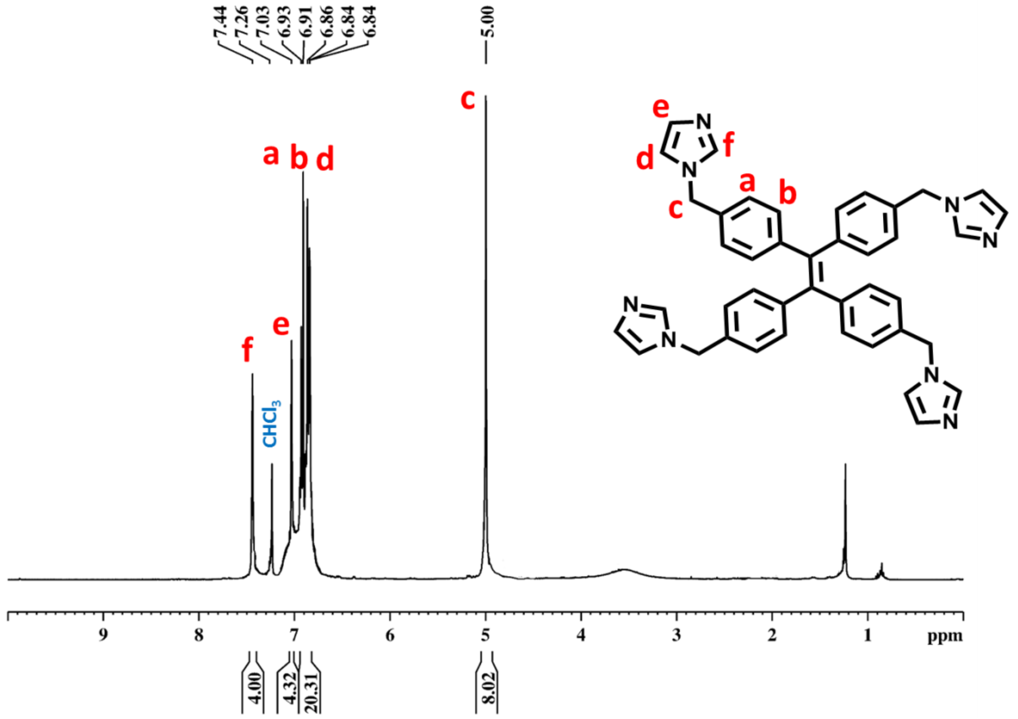

Fig. S3 ^{1}H NMR ($CDCl_3$, 400 MHz) of **L**.

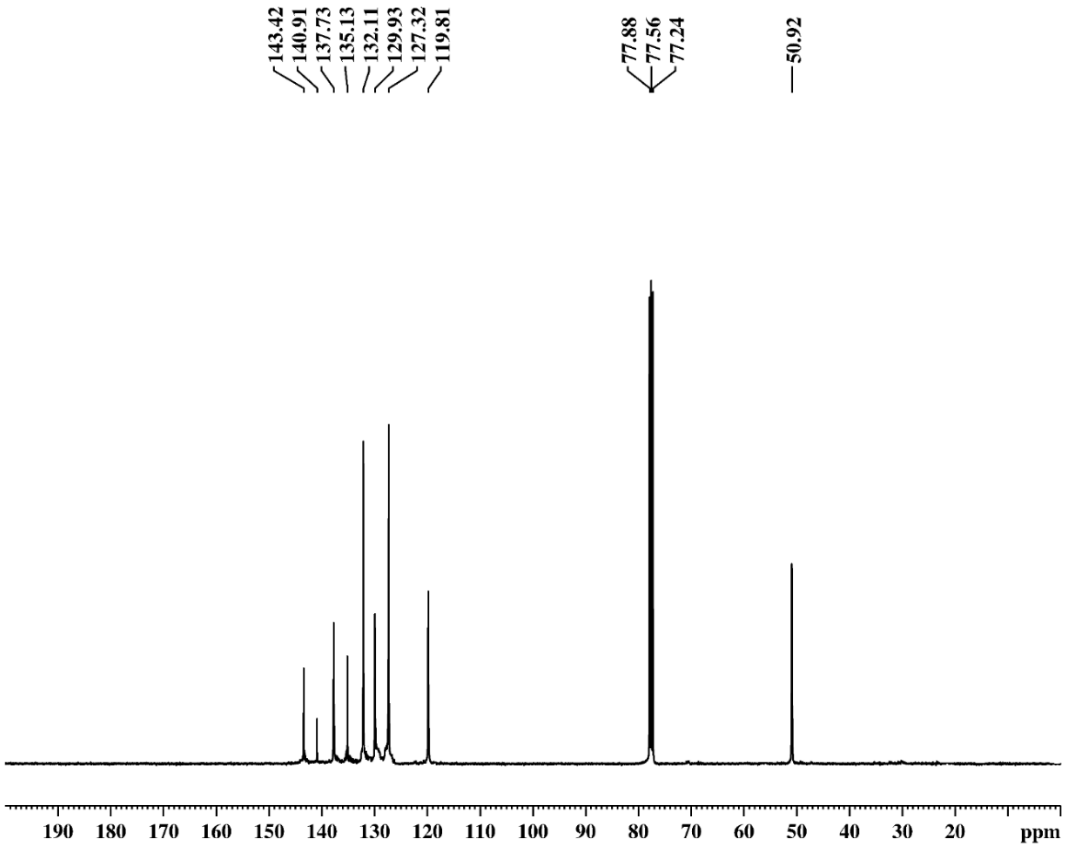

Fig. S4 ^{13}C NMR ($CDCl_3$, 100 MHz) of ligand **L**.

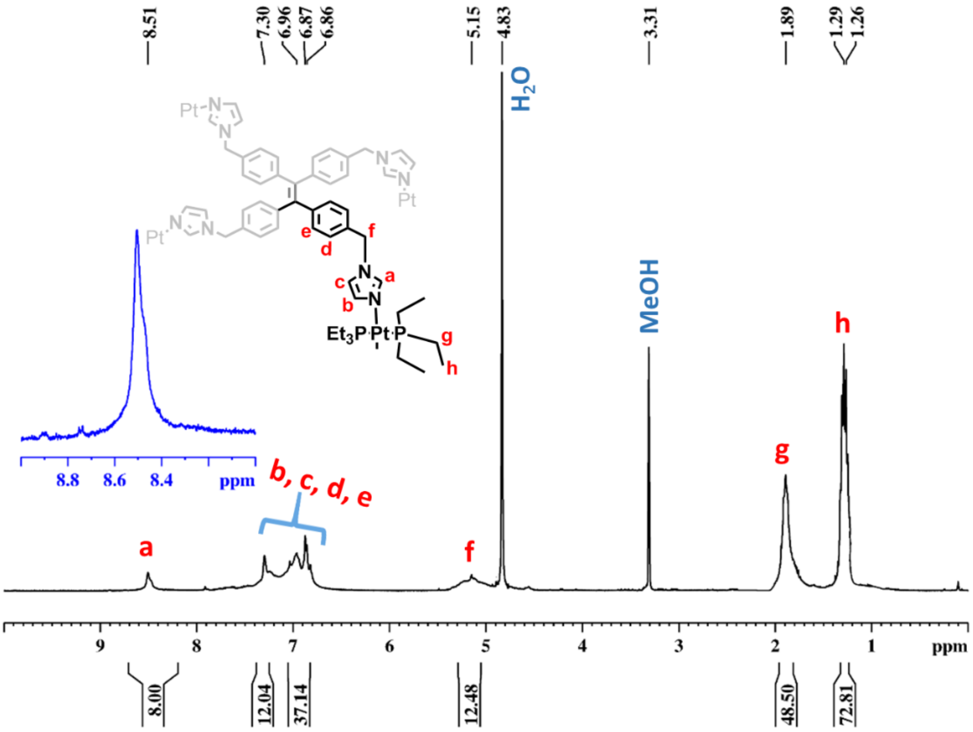

Fig. S5 ^{1}H NMR (CD_3OD, 400 MHz) of cage **1a**.

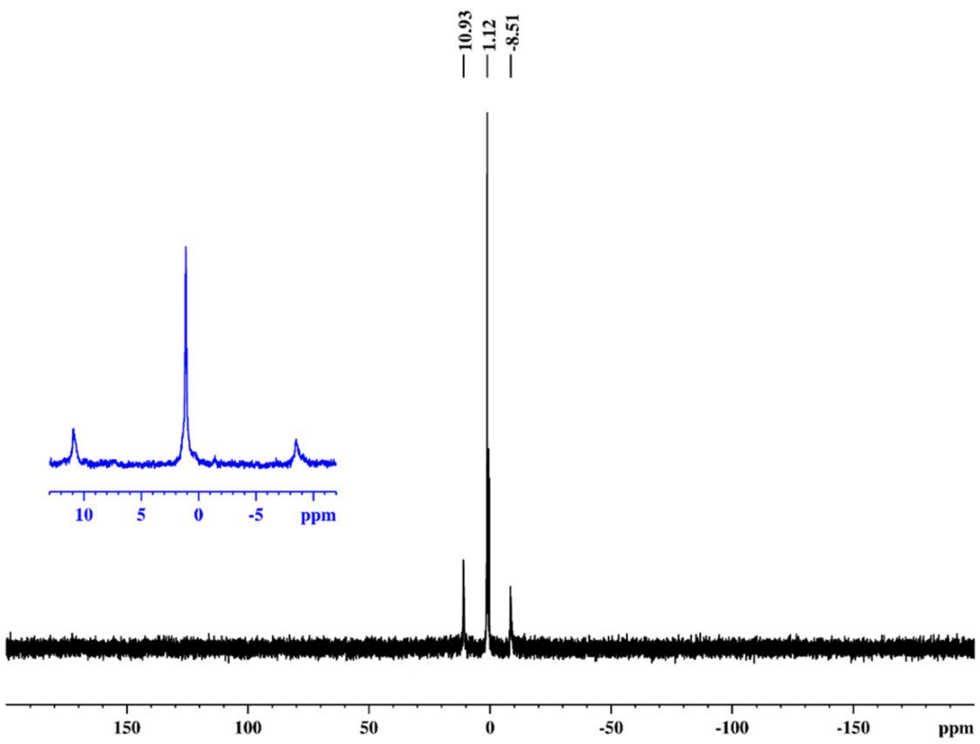

Fig. S6 $^{31}P\{^{1}H\}$ NMR (CD_3OD, 162 MHz) of cage **1a**.

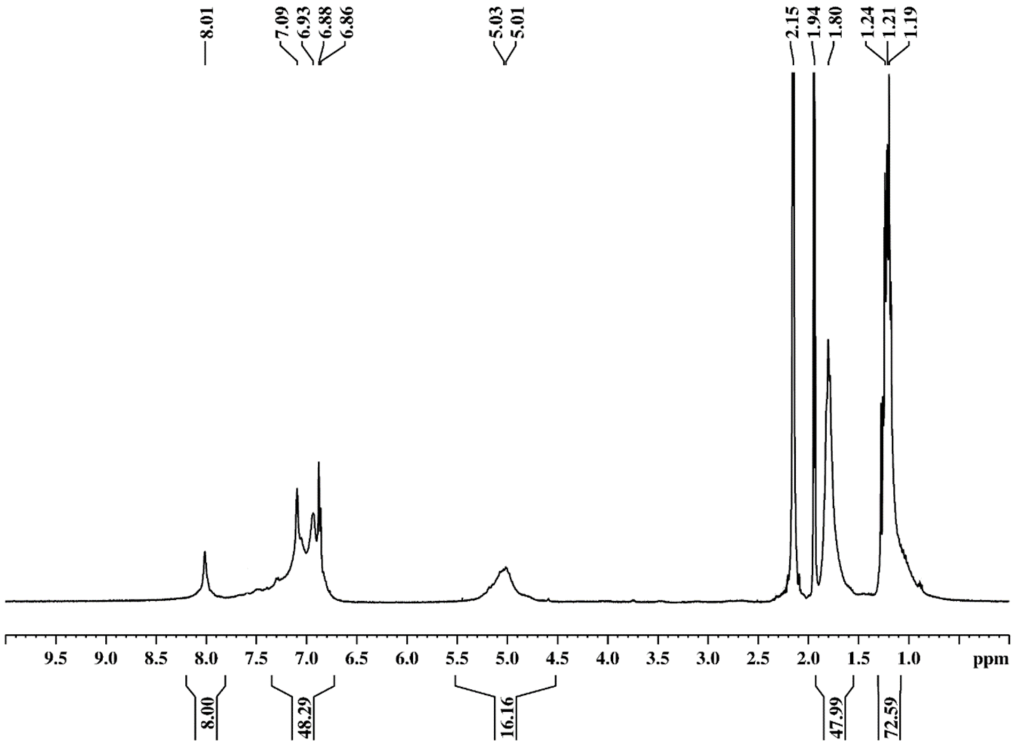

Fig. S7 ^{1}H NMR (CD_3CN, 400 MHz) of cage **1b**.

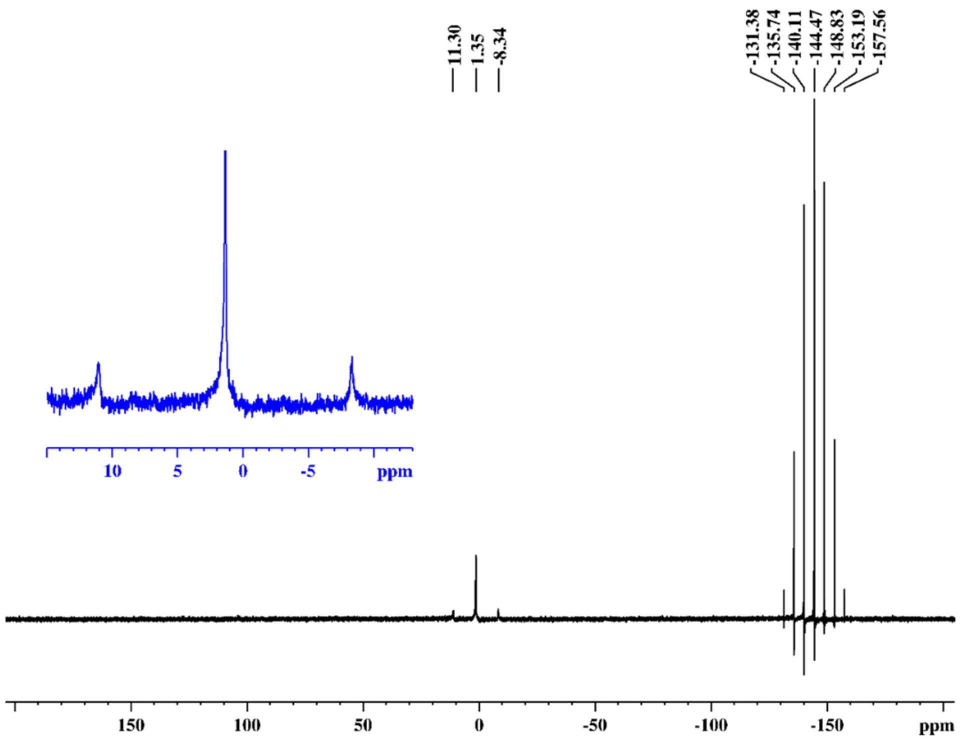

Fig. S8 $^{31}P\{^{1}H\}$ NMR (CD_3CN, 162 MHz) of cage **1b**.

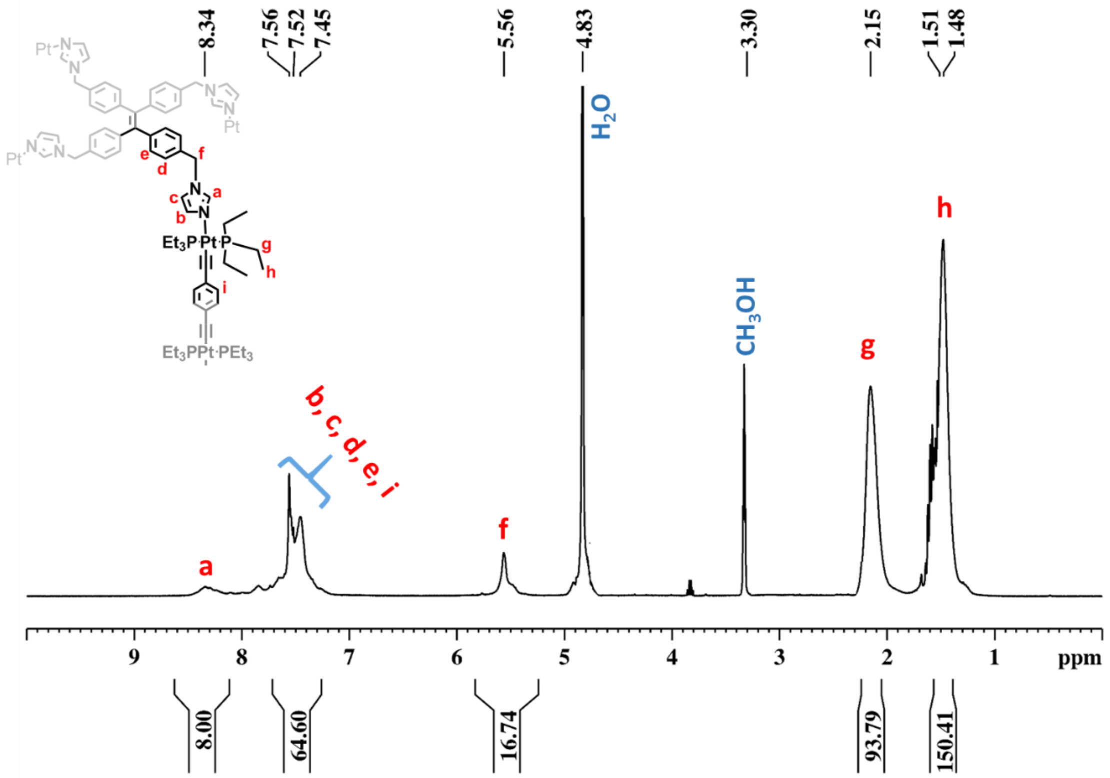

Fig. S9 ^{1}H NMR (CD_3OD, 400 MHz) of cage **2a**.

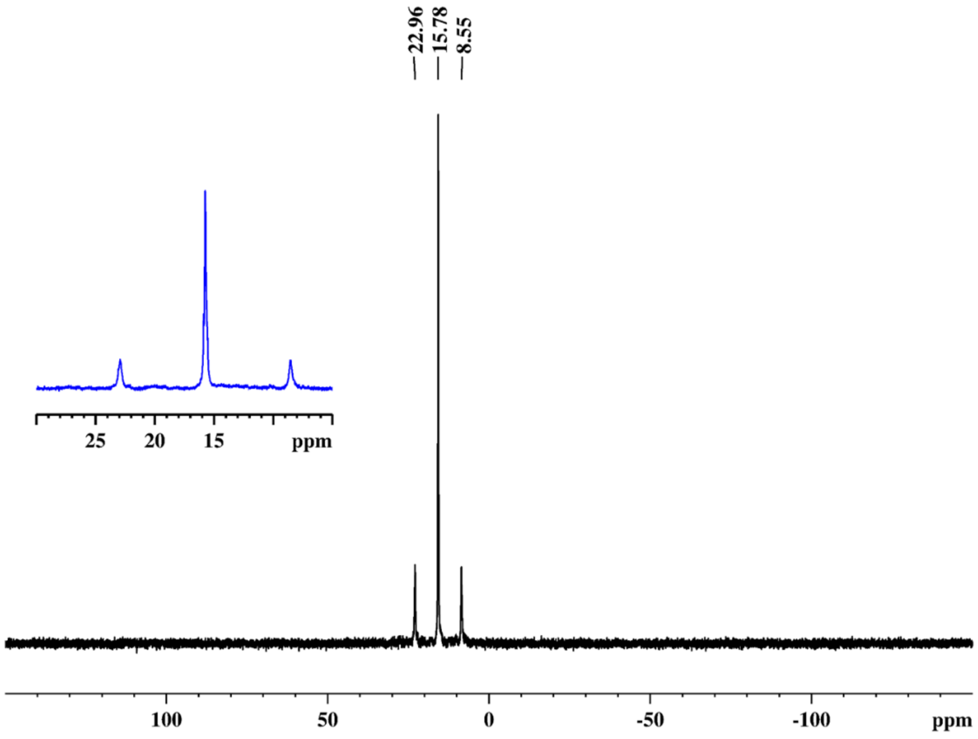

Fig. S10 $^{31}P\{^1H\}$ NMR (CD_3OD, 162 MHz) of cage **2a**.

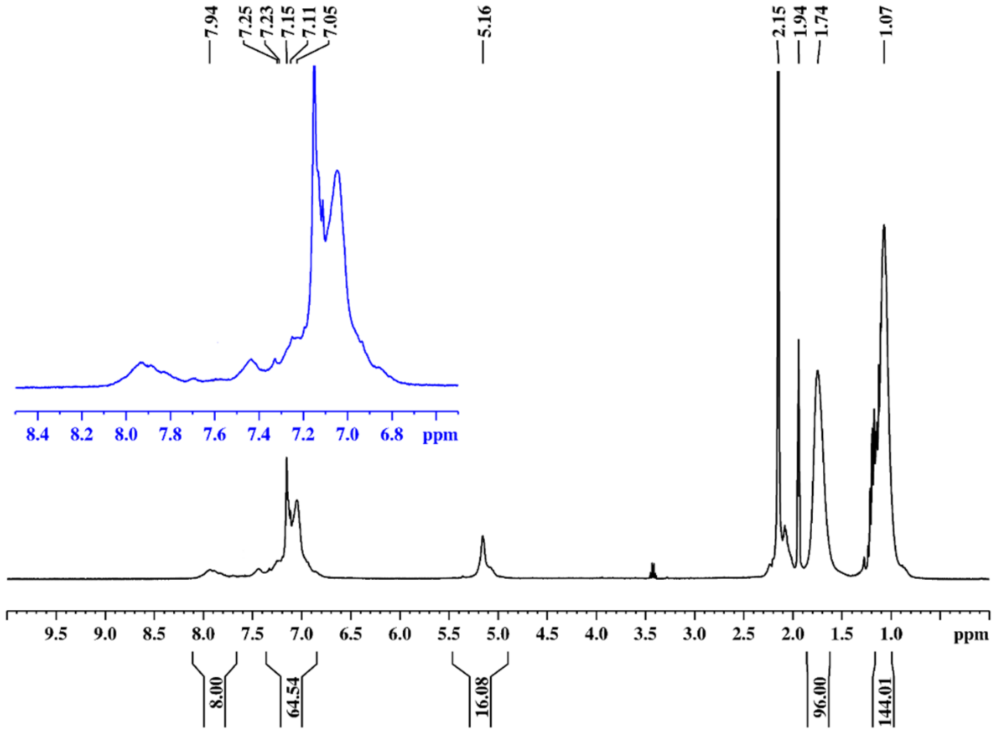

Fig. S11 ^1H NMR (CD_3CN, 400 MHz) of cage **2b**.

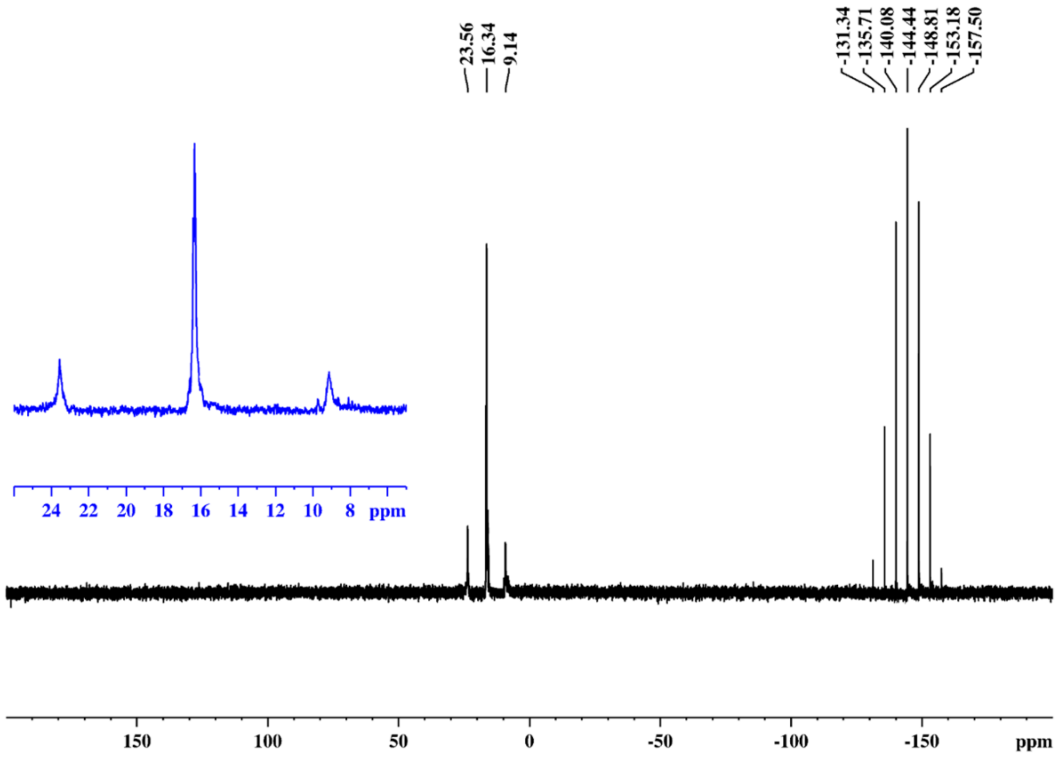

Fig. S12 $^{31}P\{^{1}H\}$ NMR (CD_3CN, 162 MHz) of cage **2b**.

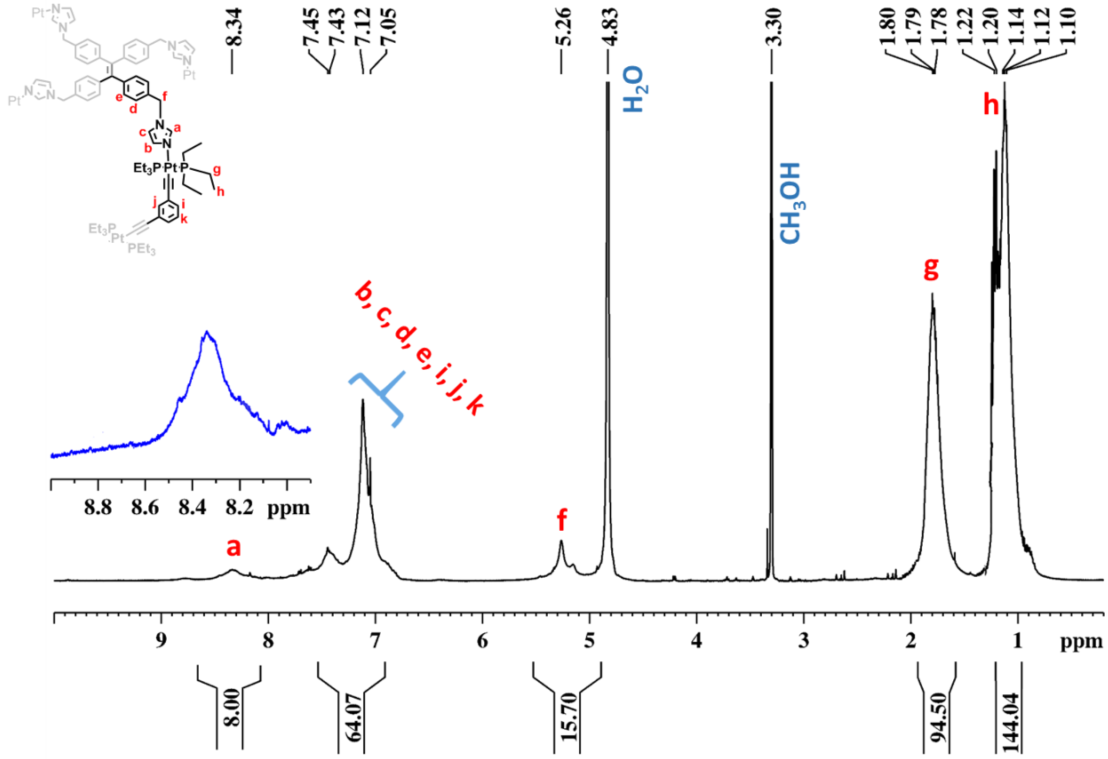

Fig. S13 ^{1}H NMR (CD_3OD, 400 MHz) of cage **3a**.

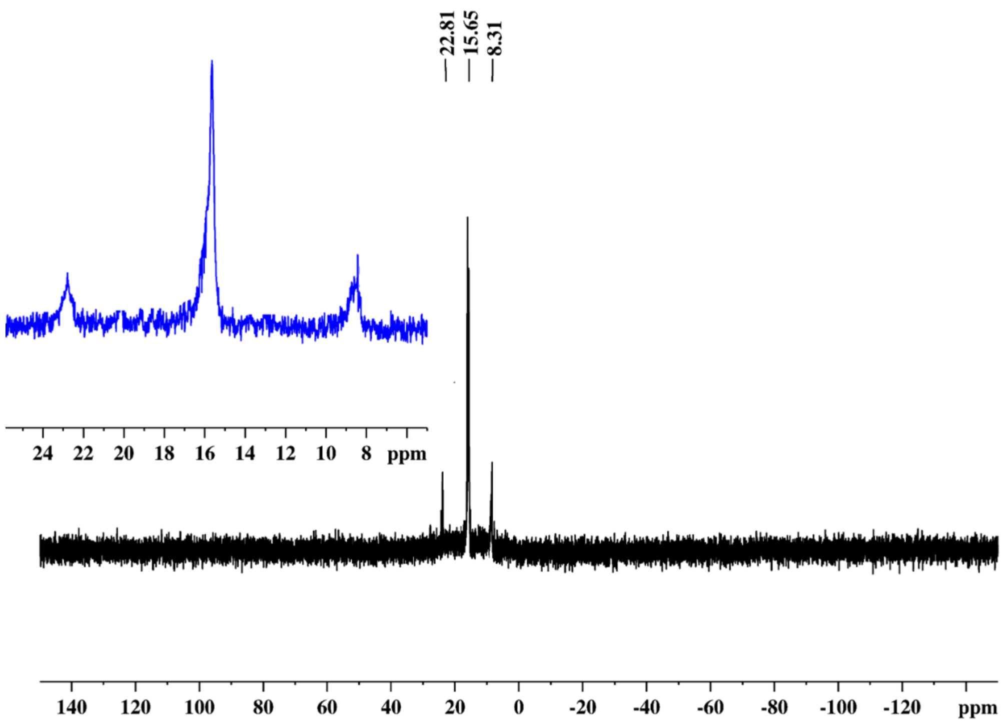

Fig. S14 $^{31}P\{^{1}H\}$ NMR (CD_3OD, 162 MHz) of cage **3a**.

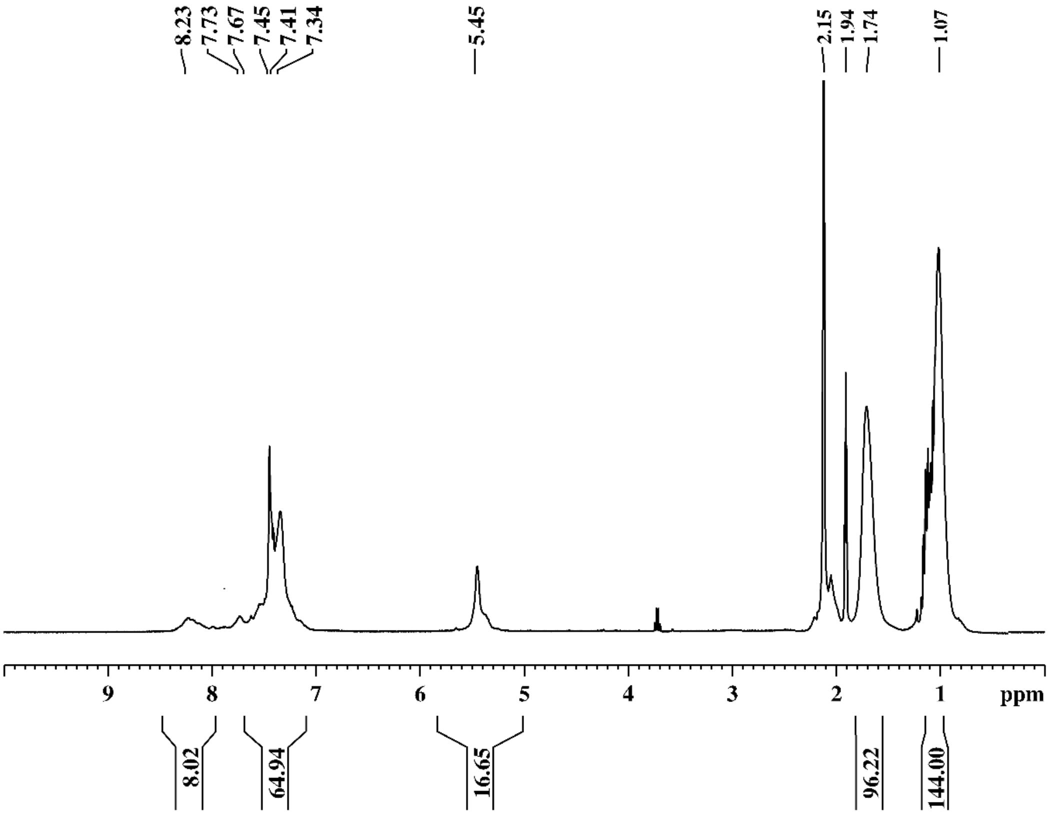

Fig. S15 ^{1}H NMR (CD_3CN, 400 MHz) of cage **3b**.

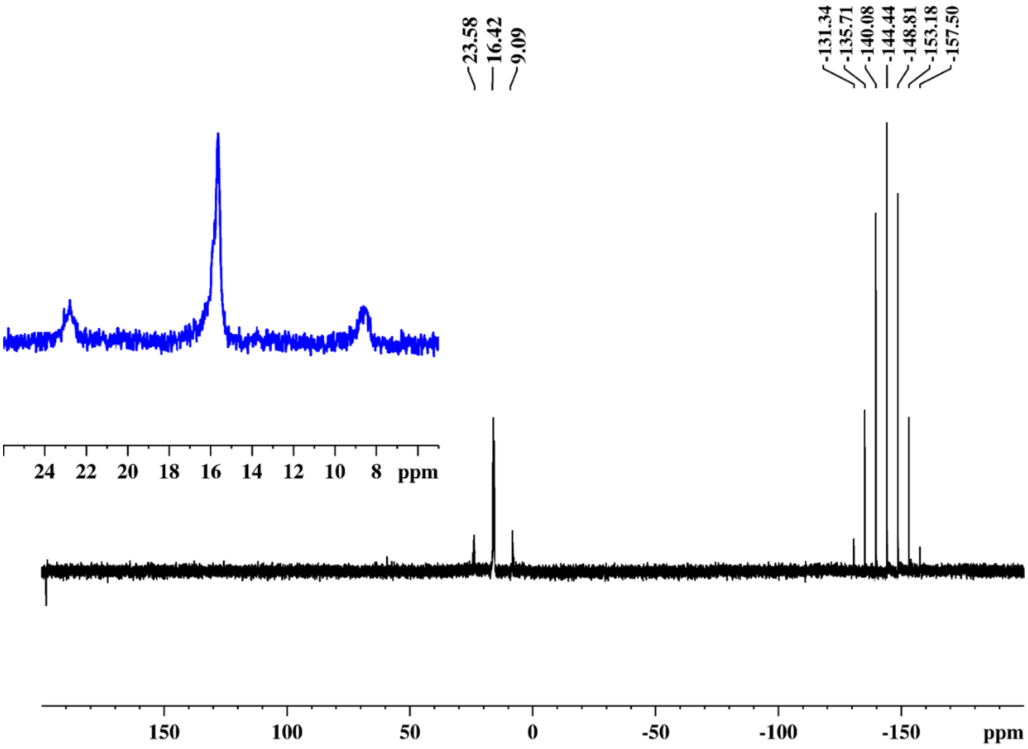

Fig. S16 $^{31}P\{^{1}H\}$ NMR (CD_3CN, 162 MHz) of cage **3b**.

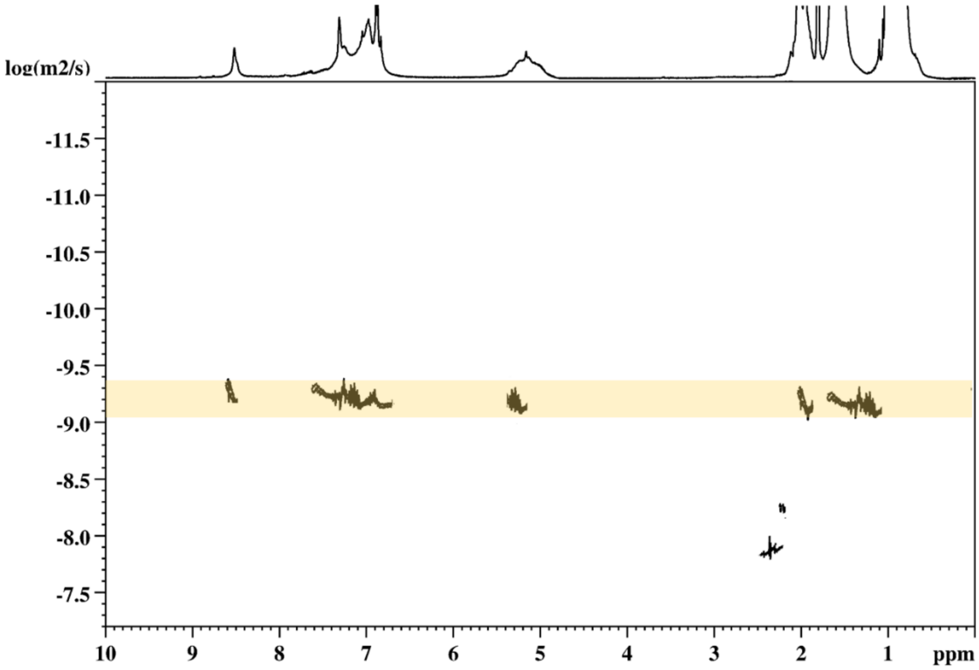

Fig. S17 DOSY NMR (CD_3CN, 400 MHz) of compound **1b**.

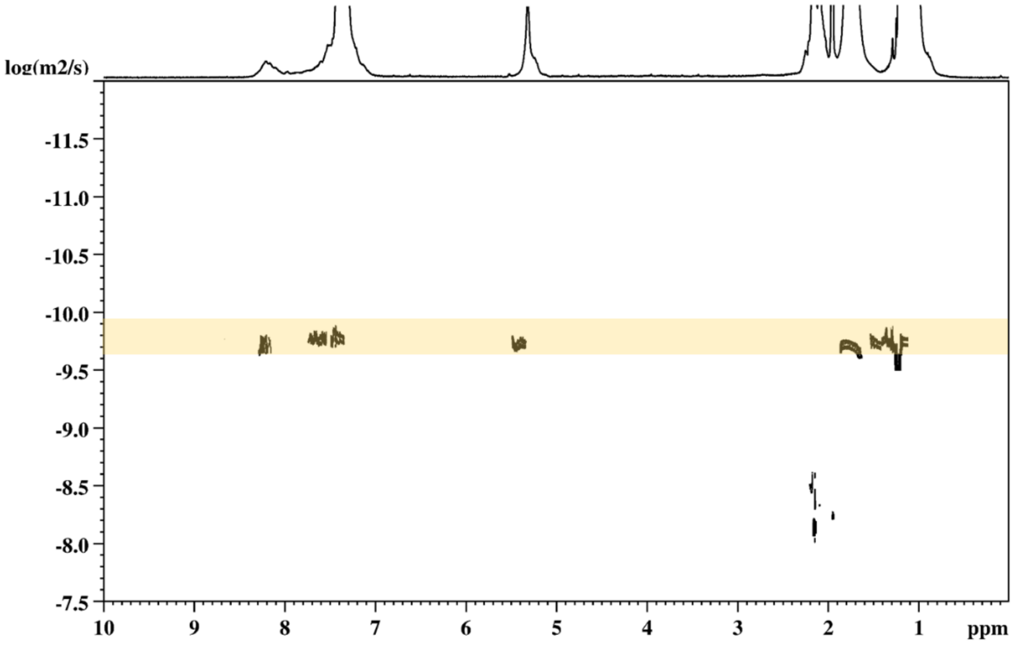

Fig. S18 DOSY NMR (CD_3CN, 400 MHz) of compound **2b**.

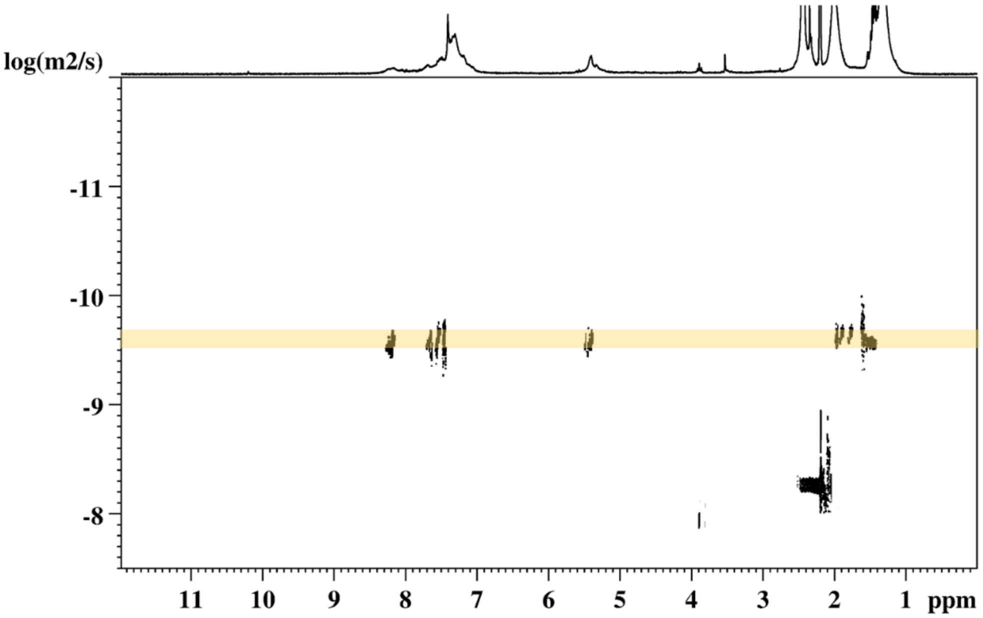

Fig. S19 DOSY NMR (CD_3CN, 400 MHz) of compound **3b**.

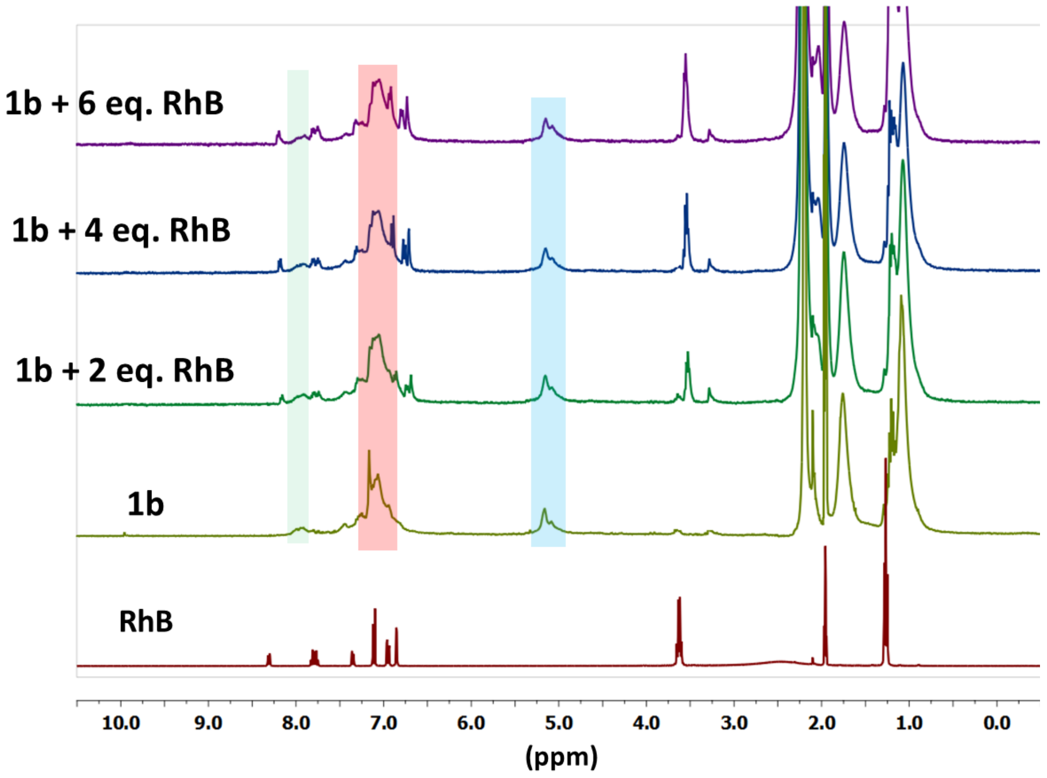

Fig. S20 ^{1}H NMR (CD_3CN, 400 MHz) titration of 10^{-3} M solution of **2b** with **RhB**.

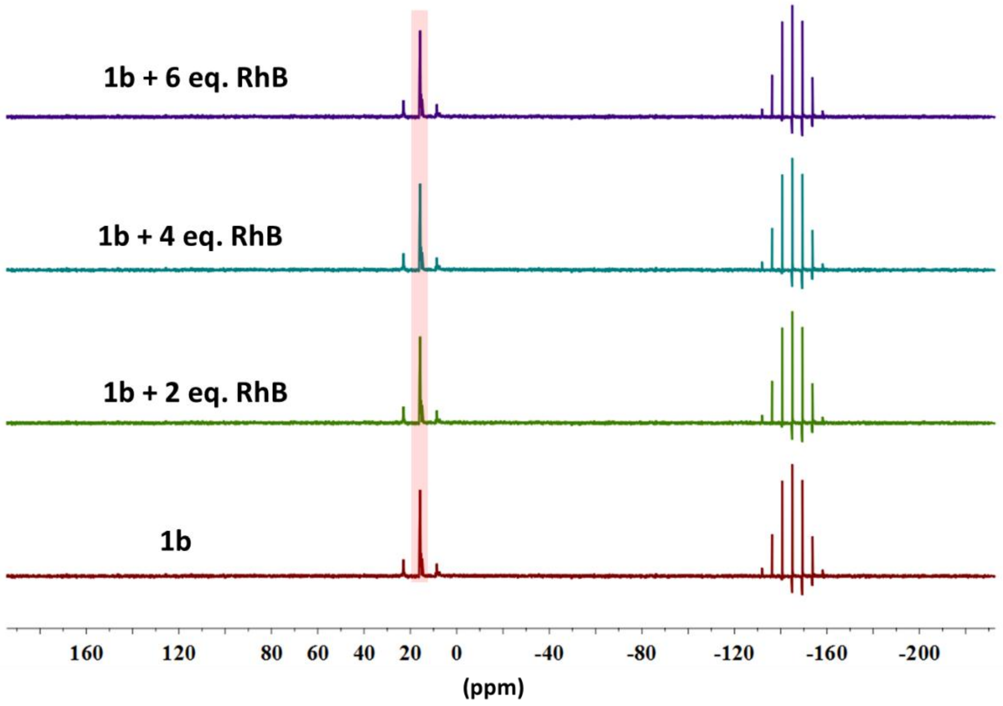

Fig. S21 ^{31}P NMR (CD_3CN, 162 MHz) titration of 10^{-3} M solution of **2b** with **RhB**.

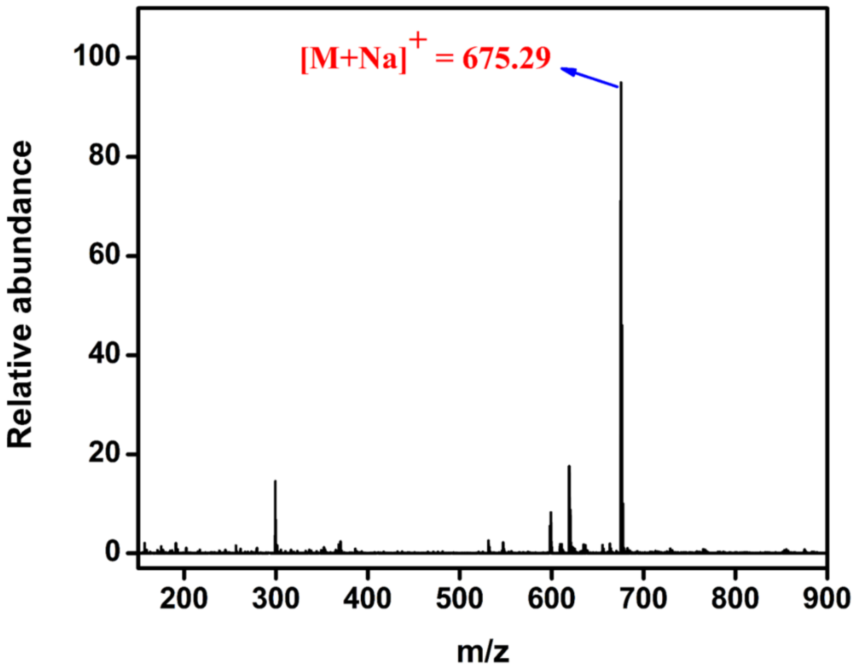

Fig. S22 ESI-MS spectrum of **L** in acetonitrile.

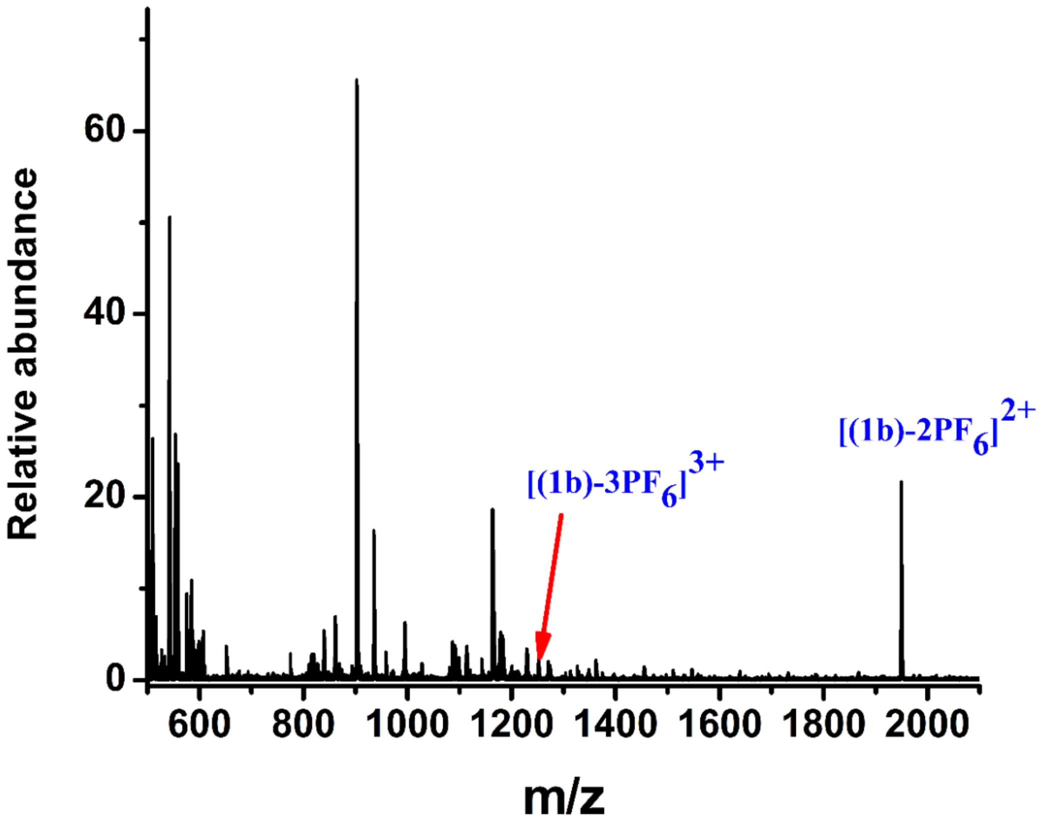

Fig. S23 ESI-MS spectrum of **1b** in acetonitrile.

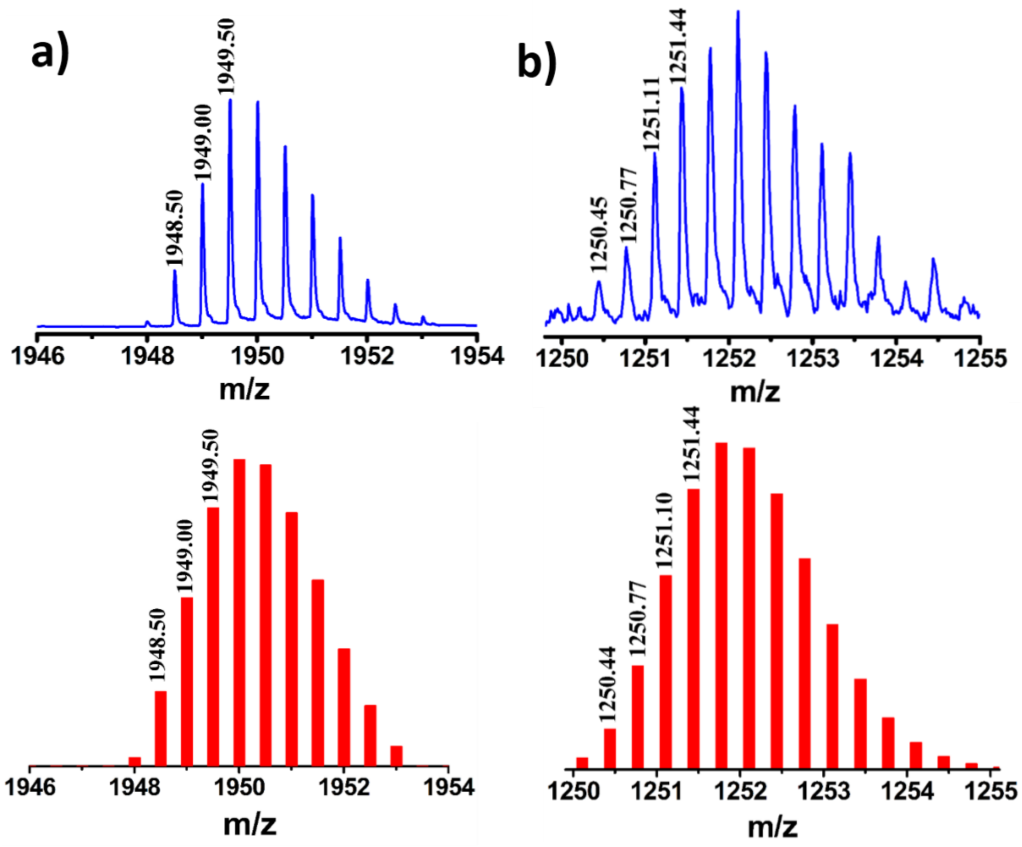

Fig. S24 Experimental (top) and theoretical (bottom) isotopic distribution patterns of the peaks corresponding to a) [(**1b**)-2PF_6^-]$^{2+}$ and b) [(**1b**)-3PF_6^-]$^{3+}$ fragments.

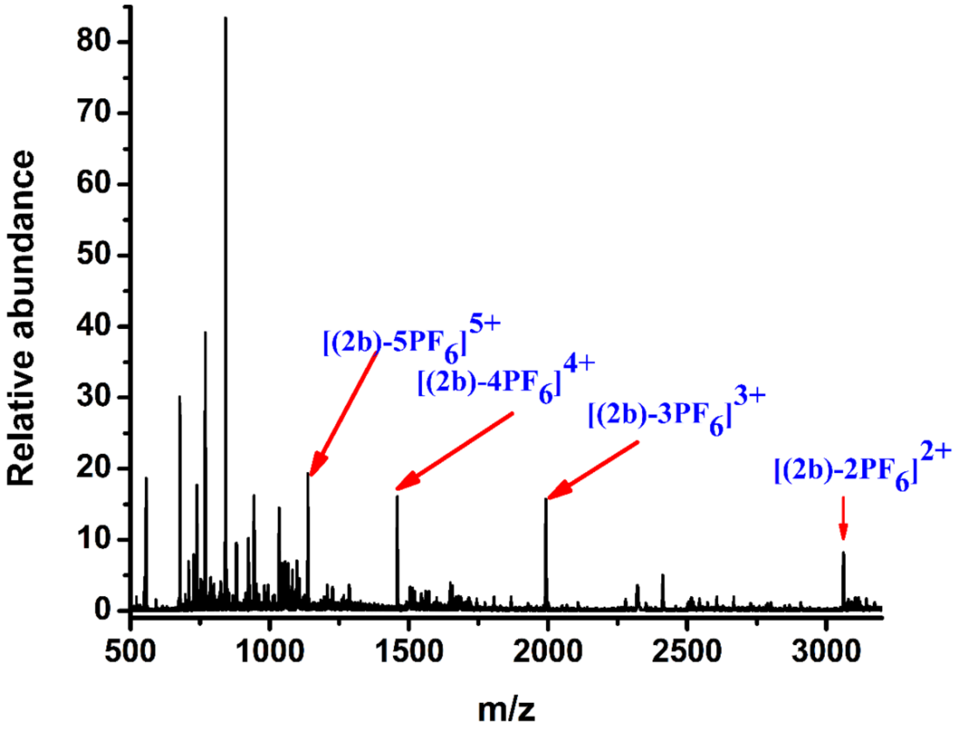

Fig. S25 ESI-MS spectrum of **2b** recorded in acetonitrile.

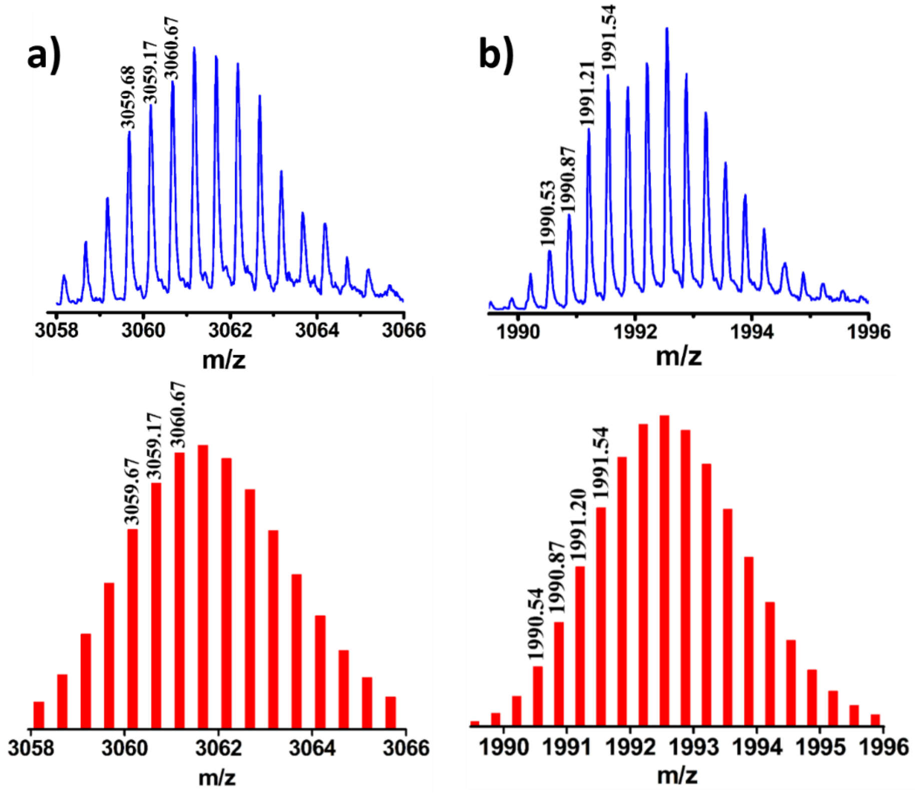

Fig. S26 Experimental (top) and theoretical (bottom) isotopic distribution patterns of the peaks corresponding to a) [**(2b)**-2PF_6^-]$^{2+}$ and b) [**(2b)**-3PF_6^-]$^{3+}$ fragments.

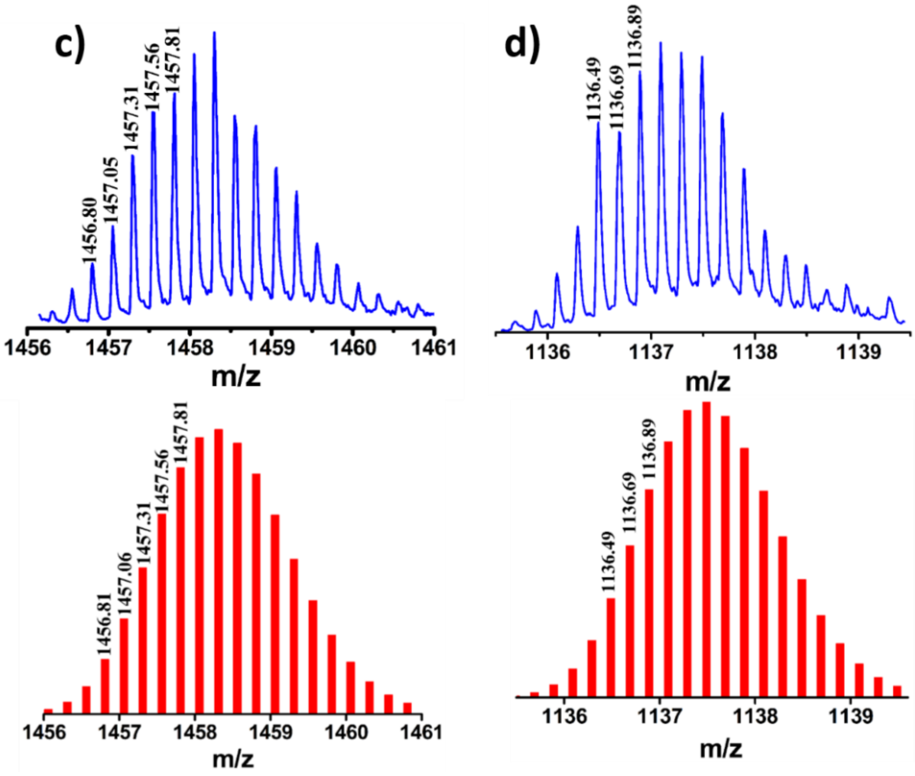

Fig. S27 Experimental (top) and theoretical (bottom) isotopic distribution patterns of the peaks corresponding to fragments a) [**(2b)**-4PF_6^-]$^{4+}$ and b) [**(2b)**-5PF_6^-]$^{5+}$.

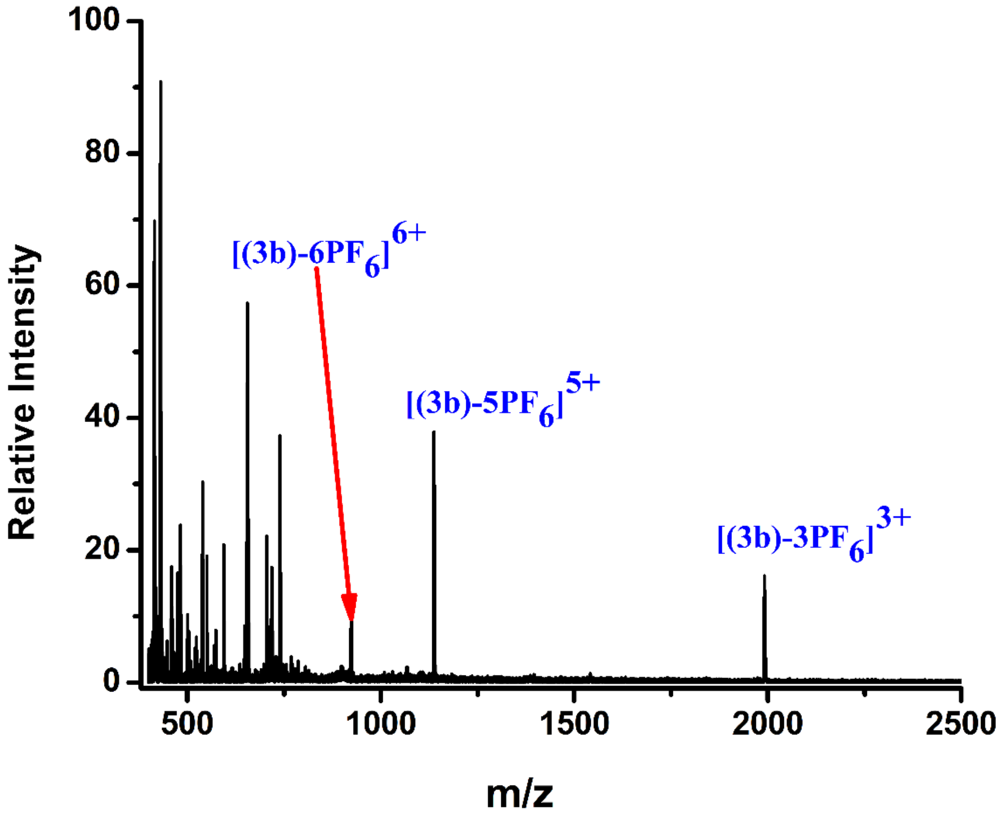

Fig. S28 ESI-MS spectrum of **3b** recorded in acetonitrile.

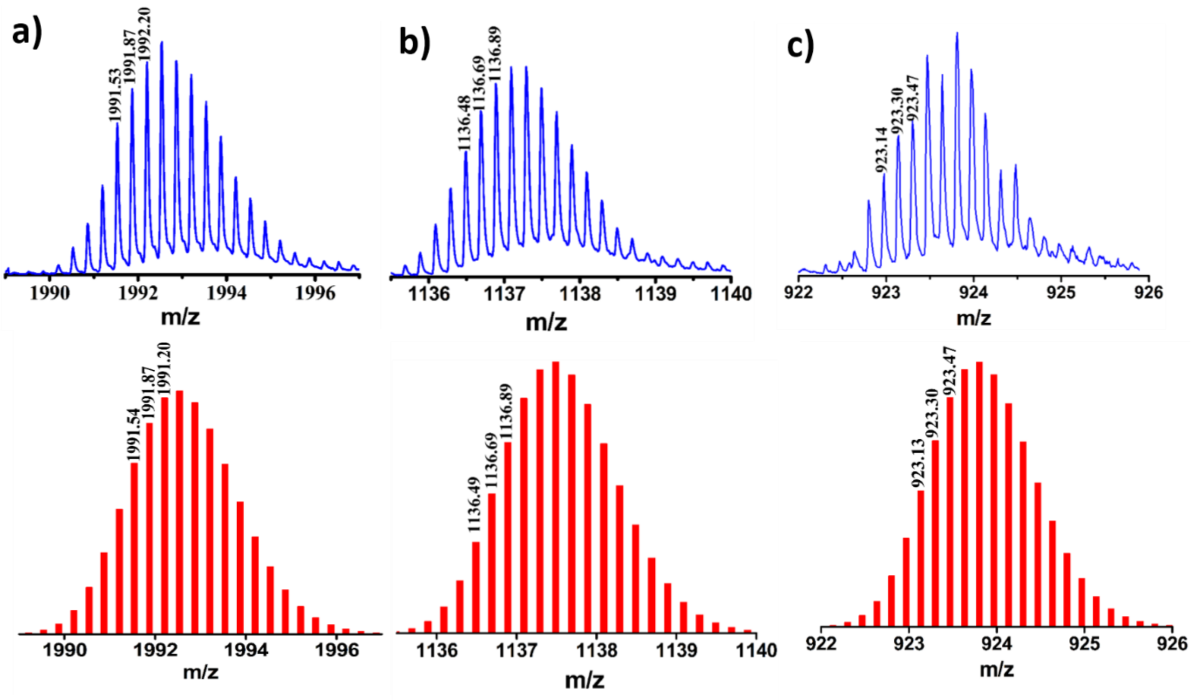

Fig. S29 Experimental (top) and theoretical (bottom) isotopic distribution patterns of the peaks corresponding to a) [(**3b**)-3PF_6^-]$^{3+}$; b) [(**3b**)-5PF_6^-]$^{5+}$ and c) [(**3b**)-6PF_6^-]$^{6+}$.

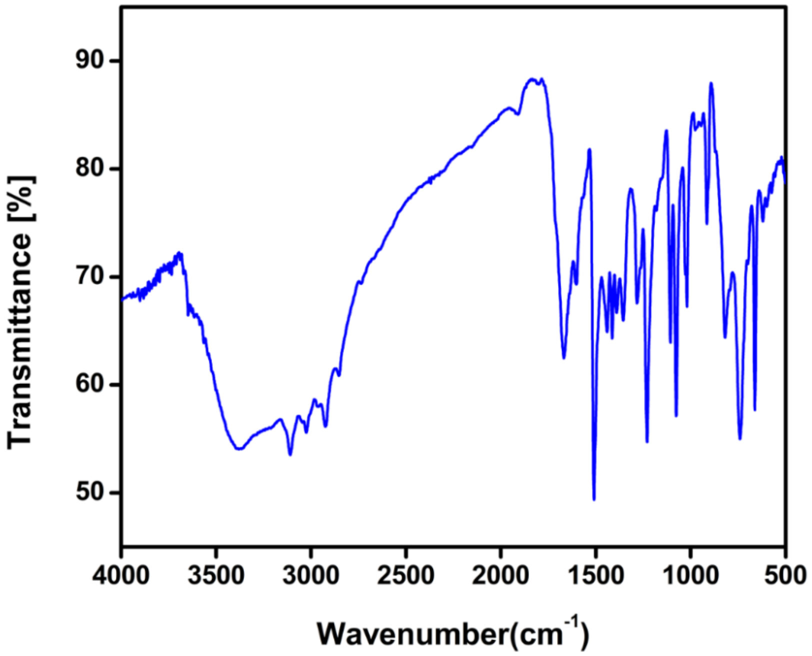

Fig. S30 FT-IR spectrum of **L**.

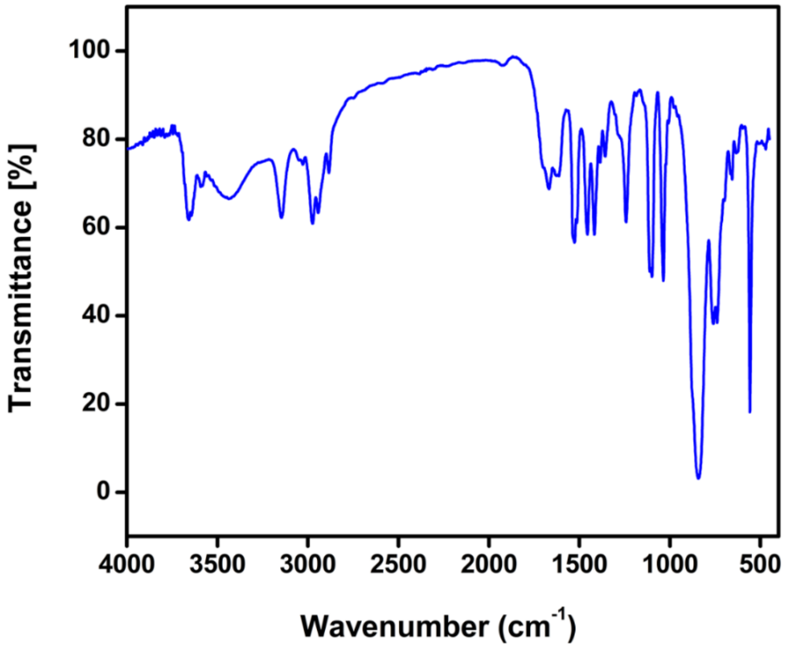

Fig. S31 FT-IR spectrum of **1b**.

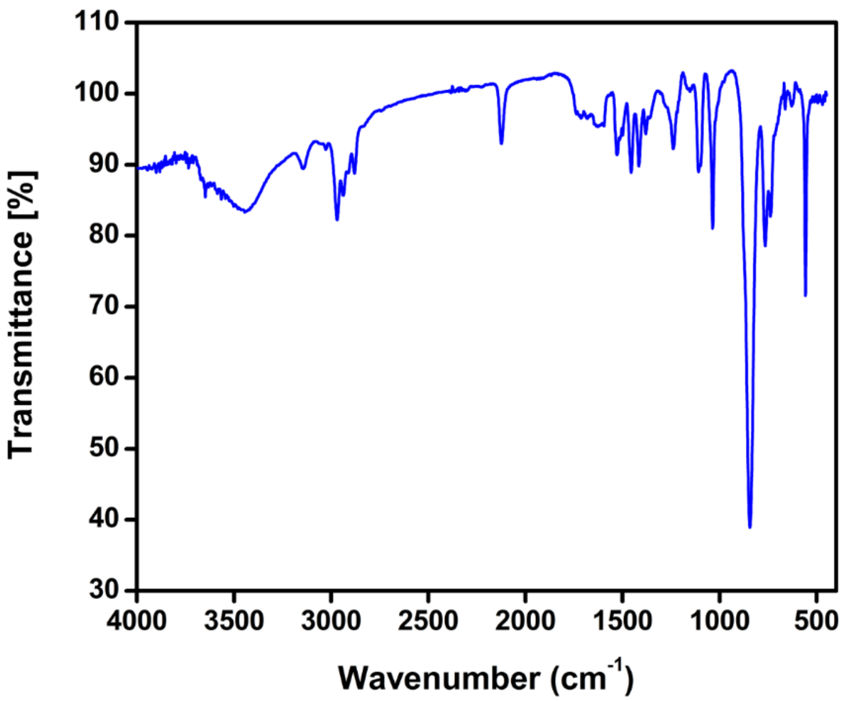

Fig. S32 FT-IR spectrum of **2b**.

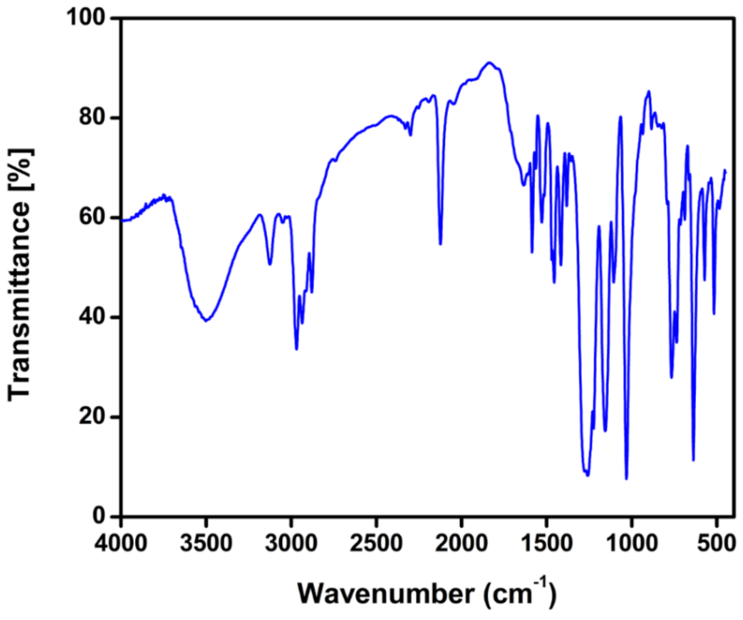

Fig. S33 FT-IR spectrum of **3b**.

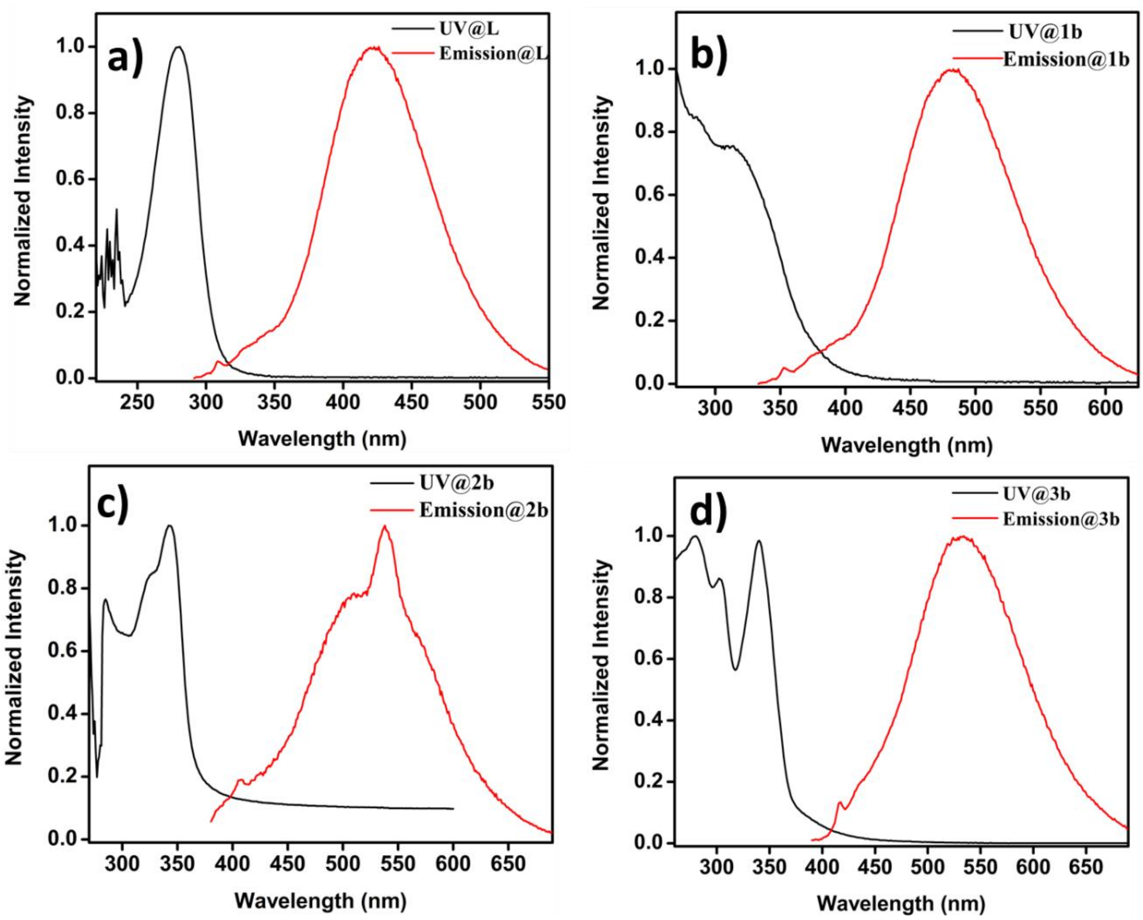

Fig. S34 Normalized UV-Vis and emission spectra in MeCN for a) **L**; b) **1b**; c) **2b** and d) **3b**.

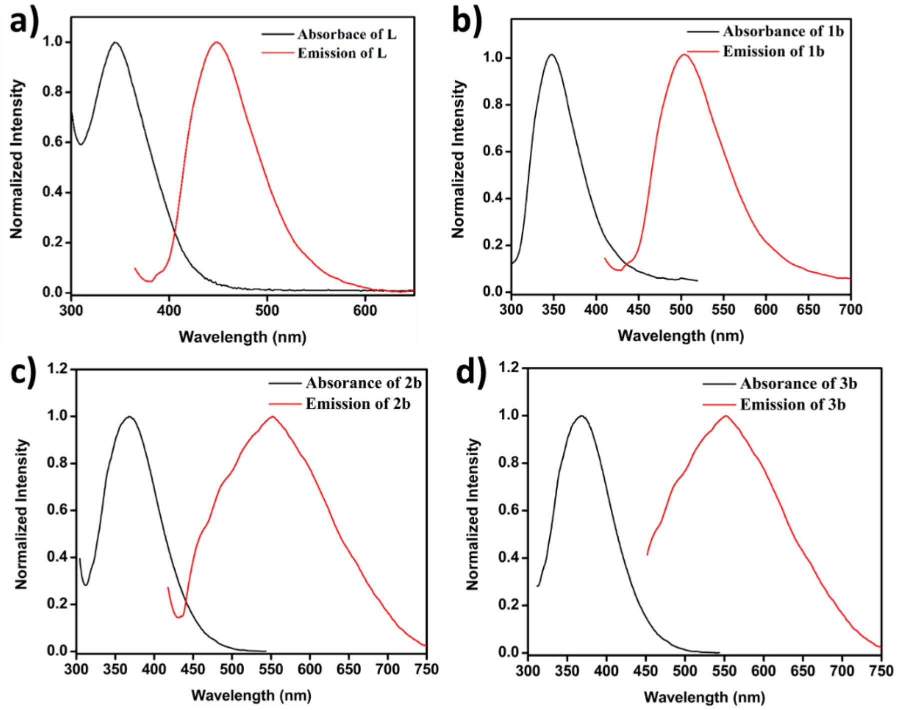

Fig. S35 Normalized UV-Vis and emission spectra in solid state for a) **L;** b) **1b;** c) **2b** and d) **3b**.

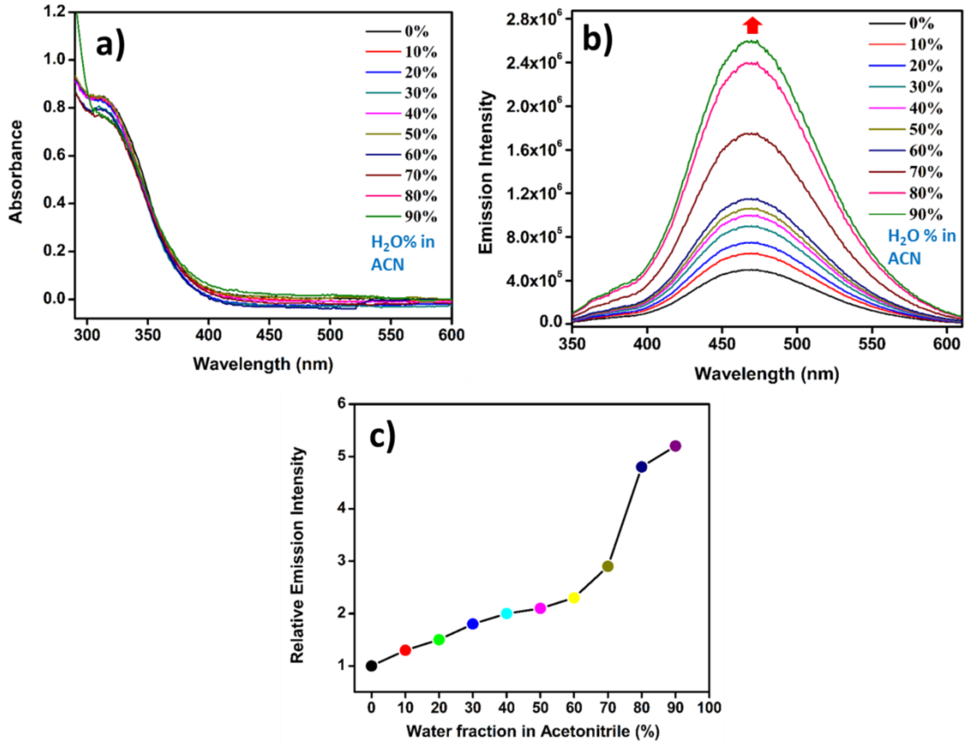

Fig. S36 a) UV-Vis spectra of **1b** in the water/MeCN mixture; b) Fluorescence emission spectra in the water/MeCN mixture ($\lambda_{ex} = 325\,nm$, c = 10^{-5} M); c) Plot of variation of relative emission intensity vs water fraction (%) in MeCN.

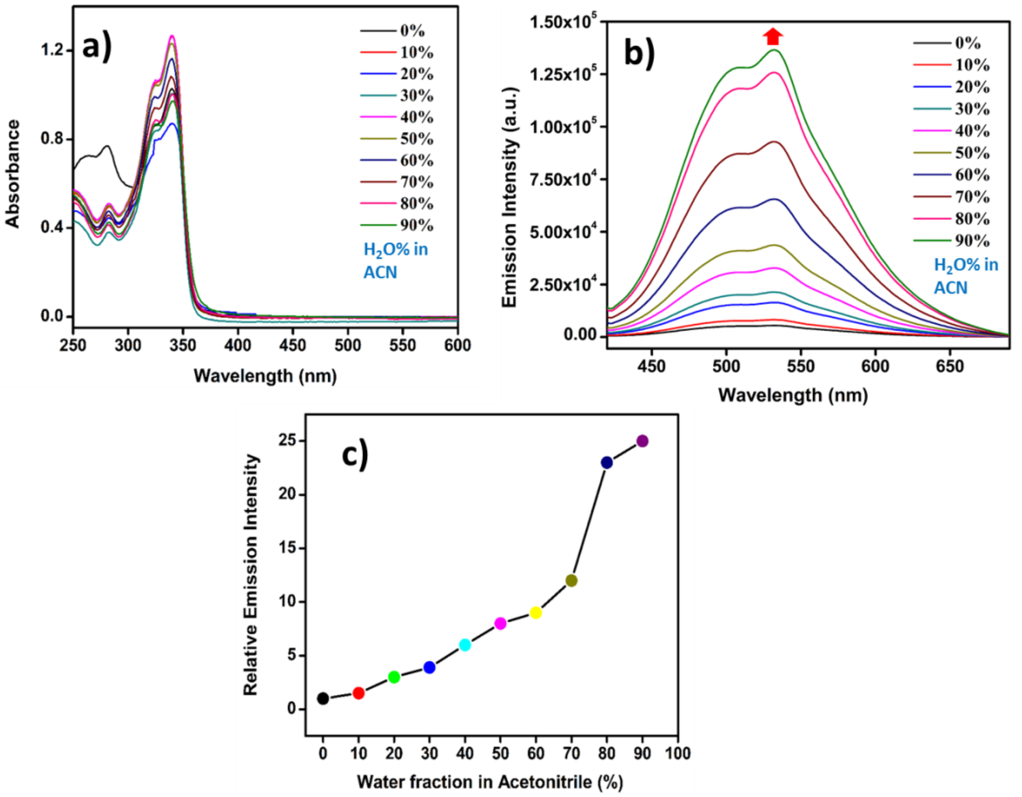

Fig. S37 a) UV-Vis spectra of **2b** in the water /MeCN mixture; b) Fluorescence Emission spectra in the water/MeCN mixture ($\lambda_{ex} = 345\,nm$, c = 10^{-5}M); c) Plot of variation of relative emission intensity vs water fraction (%) in MeCN.

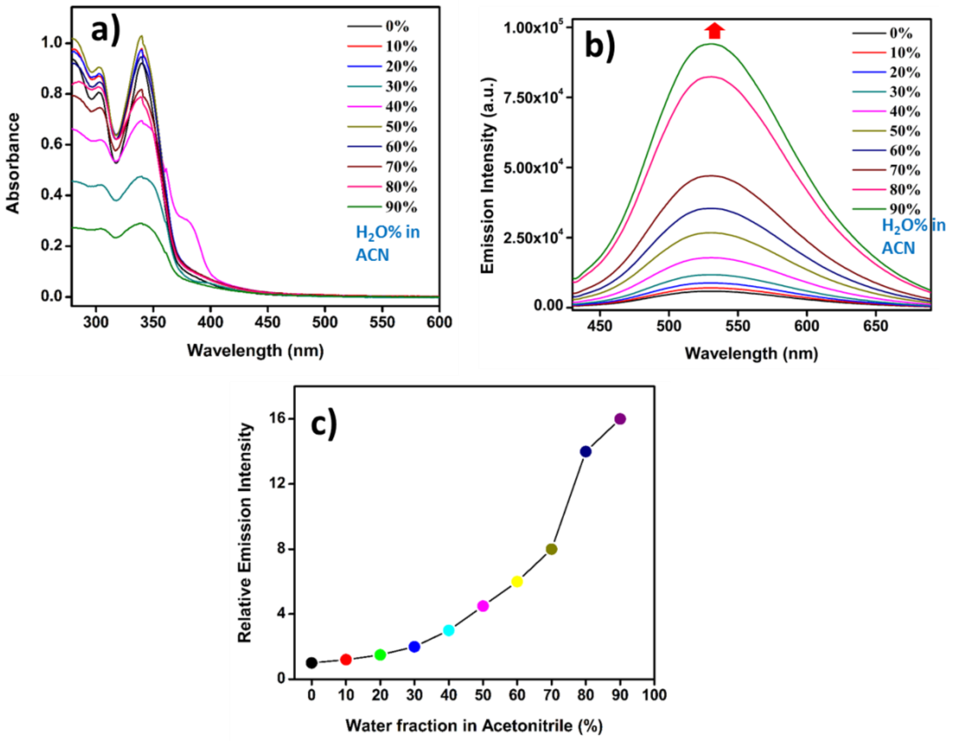

Fig. S38 a) UV-Vis spectra of **3b** in the water /MeCN mixture; b) Fluorescence Emission spectra in water/MeCN mixture ($\lambda_{ex} = 340\ nm$, c = 10^{-5}M); c) Plot of variation of relative emission intensity vs water fraction (%) in MeCN.

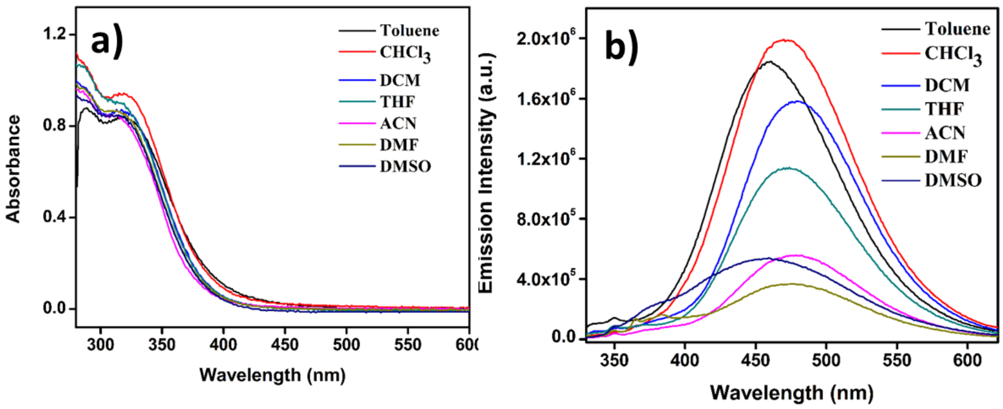

Fig. S39 a) UV-Vis spectra of **1b** in different solvents (c = 10^{-5}M); b) Fluorescence emission spectra of **1b** in different solvents ($\lambda_{ex} = 325\ nm$, c = 10^{-5}M).

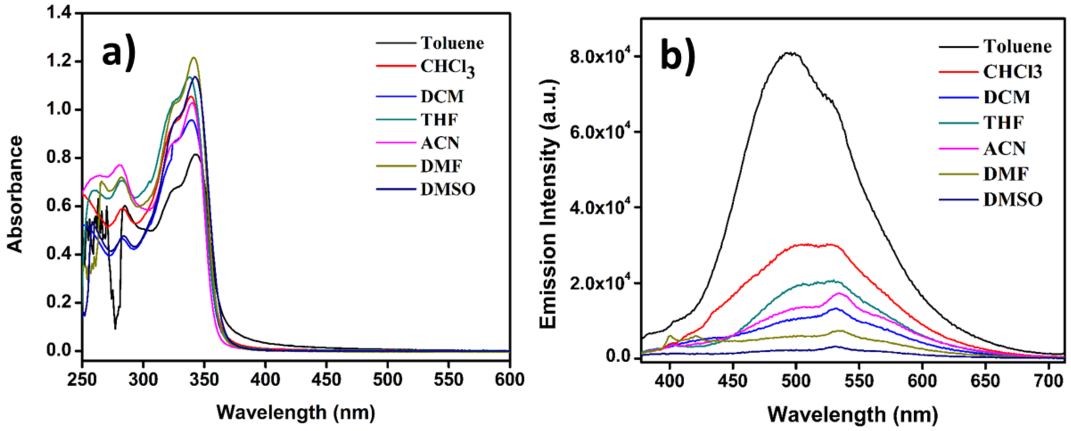

Fig. S40 a) UV-Vis spectra of **2b** in different solvents (c = 10^{-5}M); b) Fluorescence emission spectra of **2b** in different solvents ($\lambda_{ex} = 345\ nm$, c = 10^{-5}M).

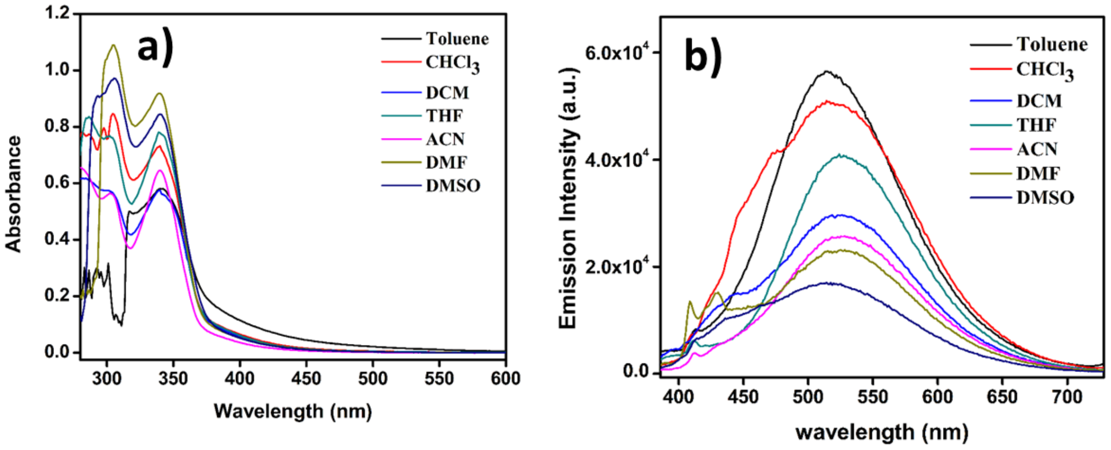

Fig. S41 a) UV-Vis spectra of **3b** in different solvents (c = 10^{-5}M); b) Fluorescence emission spectra of **3b** in different solvents ($\lambda_{ex} = 340\ nm$, c = 10^{-5}M).

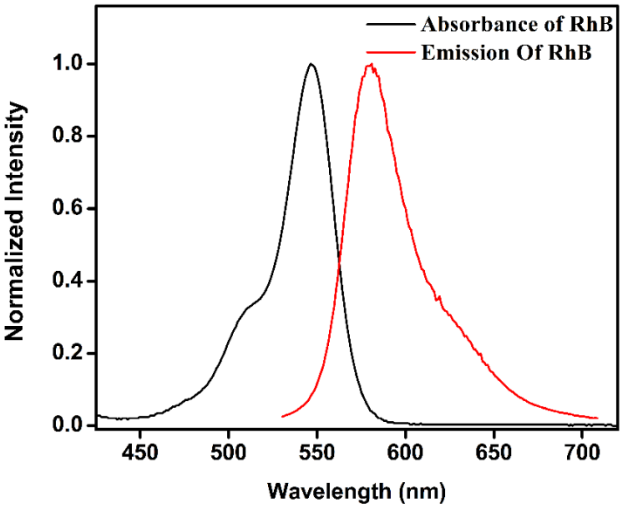

Fig. S42 a) UV-Vis spectra of **RhB** in 90% water in MeCN (c = 10^{-5}M); b) Fluorescence emission spectra of **RhB** in 90% water in MeCN ($\lambda_{ex} = 547\ nm$, c = 10^{-5}M).

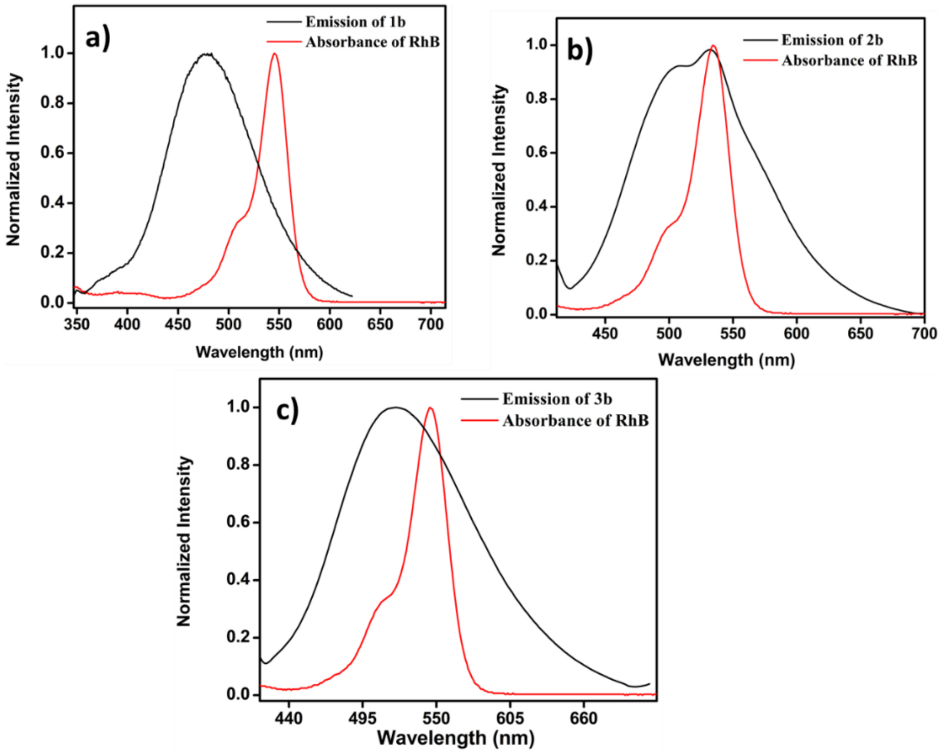

Fig. S43 Normalized plots of a) Emission of **1b** and absorbance of **RhB**; b) Emission of **2b** and absorbance of **RhB**; c) Emission of **3b** and absorbance of **RhB.**

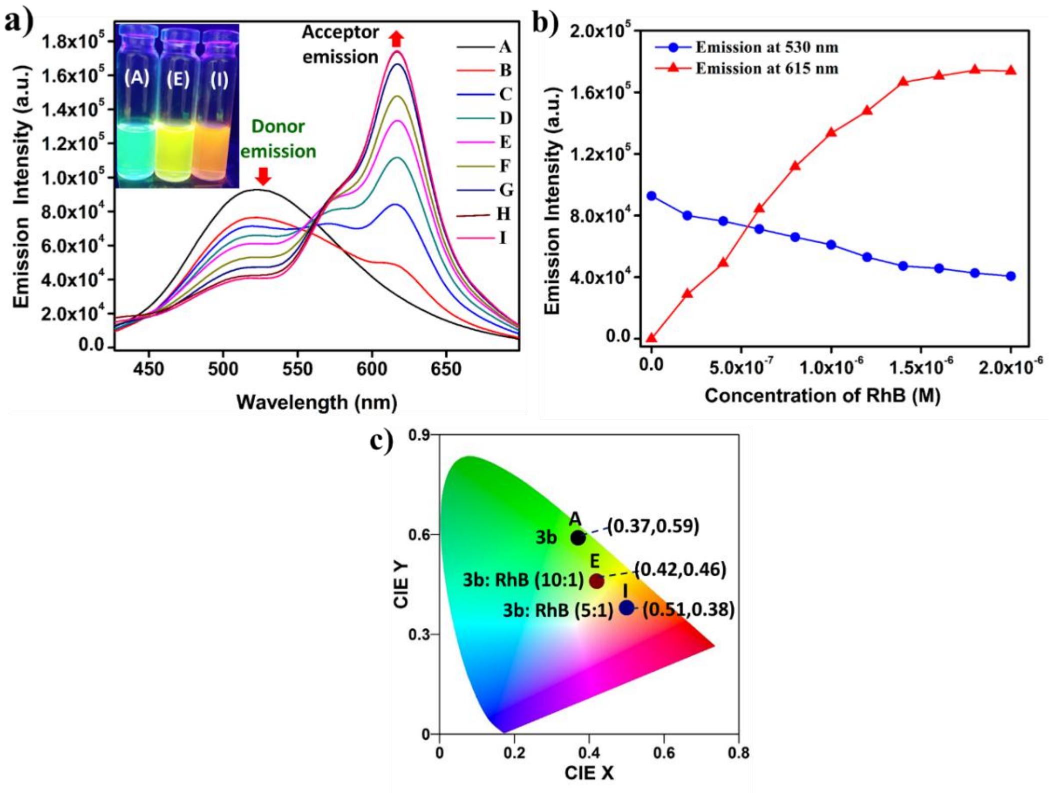

Fig. S44 a) Fluorescence spectra of metallacage **3b** with gradual addition of **RhB** ($\lambda_{ex} = 345\,nm$) in 90% H_2O/MeCN mixture. (Inset) photograph of **3b** (left), (**3b**+**RhB**) (10:1 equiv.) (middle), (**3b**+**RhB**) (5:1 equiv.) (right) under UV irradiation at 365 nm, b)

Fluorescence intensity change in **3b** at 530 and 615nm, c) the 1931 CIE chromaticity coordinate changes of **3b** (10^{-5}M in 90% water-MeCN mixture) titrated by **RhB** (0 to 0.2 equiv.).

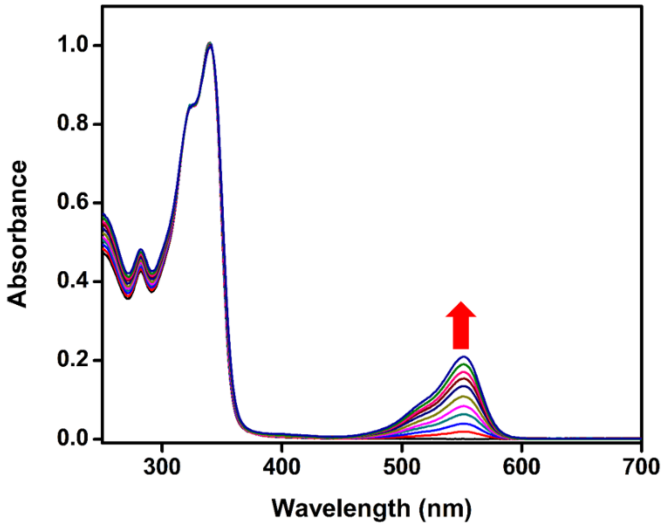

Fig. S45 UV-visible of **2b** (λ_{max} = 345 nm) with gradual addition of **RhB** in 90% water in MeCN {2 ml of 10^{-5} M cage solution (90% water/MeCN mixture) was titrated with 2×10^{-5} M solution of **RhB** (λ_{max} = 547 nm) in 90% water/MeCN mixture.

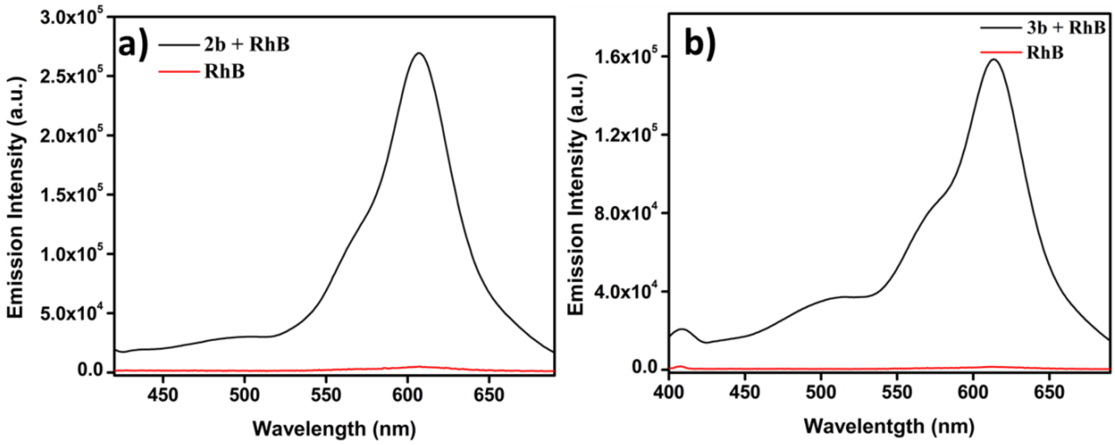

Fig. S46 a) Fluorescence emission spectra ($\lambda_{ex} = 345\ nm$) of **RhB** in presence of **2b** (**2b** :**RhB** :: 5:1) (black line) and in absence of **2b** (red line). **b)** Fluorescence emission spectra ($\lambda_{ex} = 340\ nm$) of **RhB** in presence of **2b** (**2b** : **RhB** :: 5:1). 90% water/MeCN medium was used for this study.

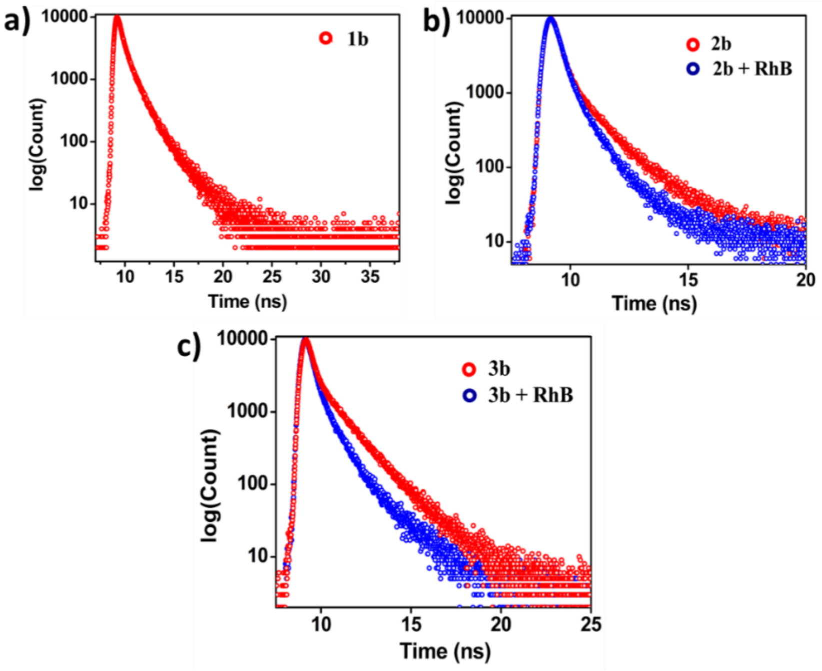

Fig. S47 Fluorescence decay profile for a) **1b** (red, λ_{ex} = 320 nm) {[**1b**] = 10^{-5} M} in 90% water/MeCN; b) **2b** (red, λ_{ex} = 340 nm) and (**2b**+**RhB**) (blue, λ_{ex} = 340 nm) {[**2b**] = 10^{-5} M, [RhB] = 2 × 10^{-6} M} in 90% water/MeCN; c) **3b** (red, λ_{ex} = 340 nm) and (**3b**+**RhB**) (blue, λ_{ex} = 340 nm) {[**3b**] = 10^{-5} M, [**RhB**] = 2 × 10^{-6} M} in 90% water/MeCN.

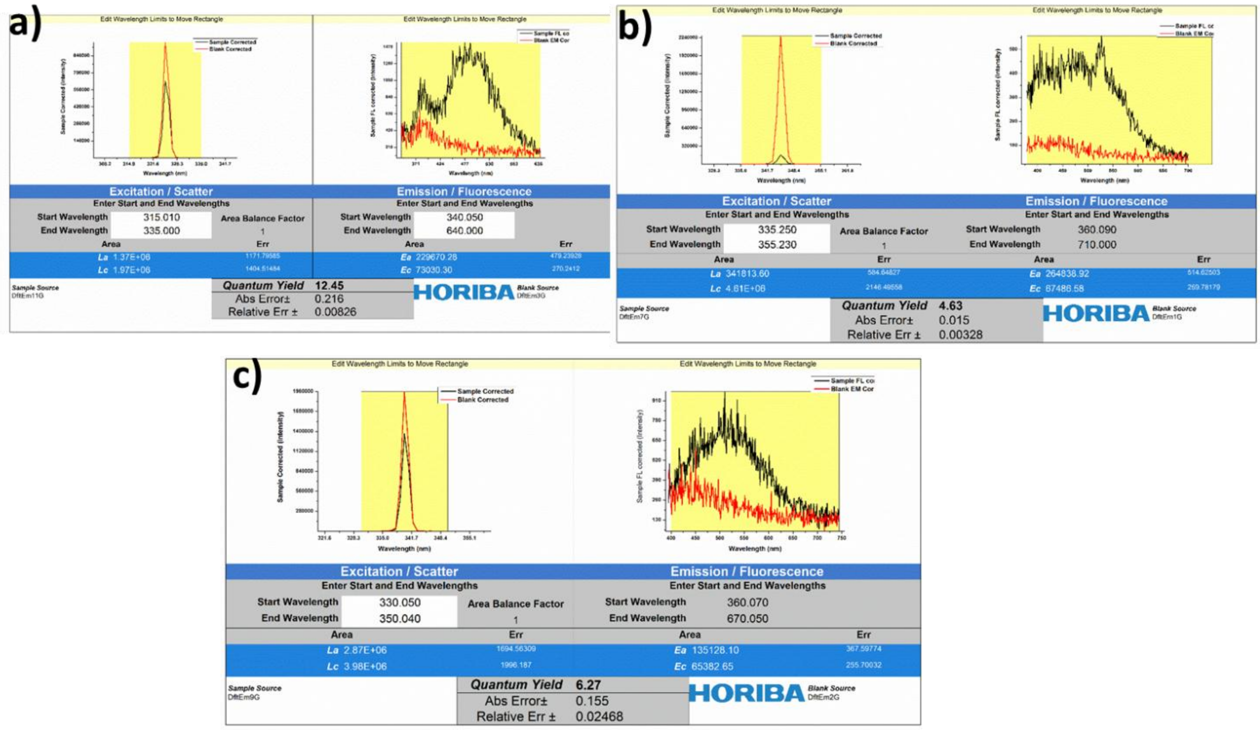

Fig. S48 Absolute fluorescence quantum yields of the cages in MeCN (a) **1b**; b) **2b**; and c) **3b**. [c = 10^{-5}M; excitation/emission slit width: 2/2].

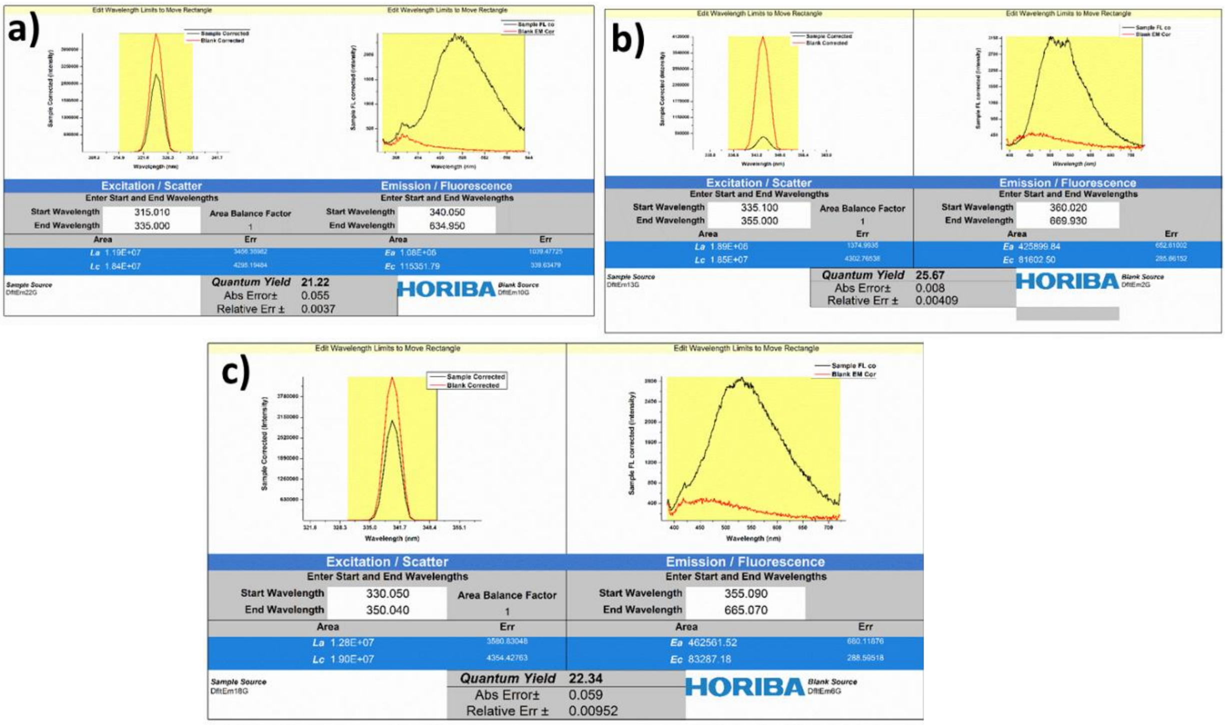

Fig. S49 Absolute fluorescence quantum yields in 90% water/MeCN for (a) **1b**; b) **2b**; and c) **3b**. [c = 10^{-5}M; excitation/emission slit width: 2/2].

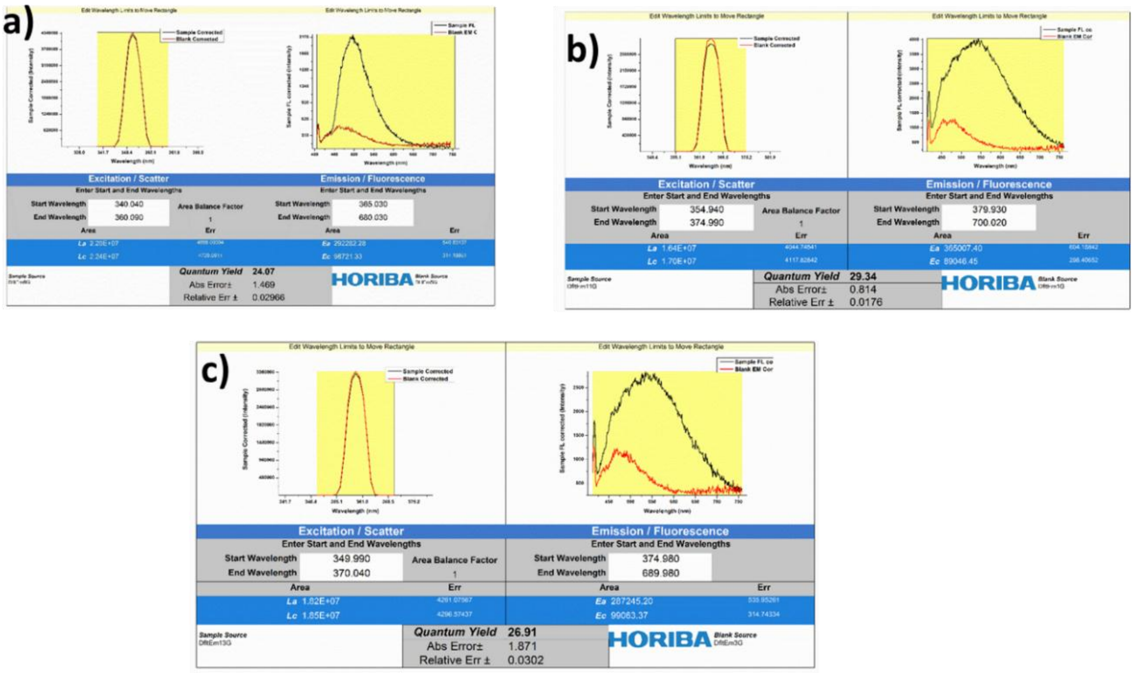

Fig. S50 Absolute fluorescence quantum yields in solid state for (a) **1b**; b) **2b**; and c) **3b.**

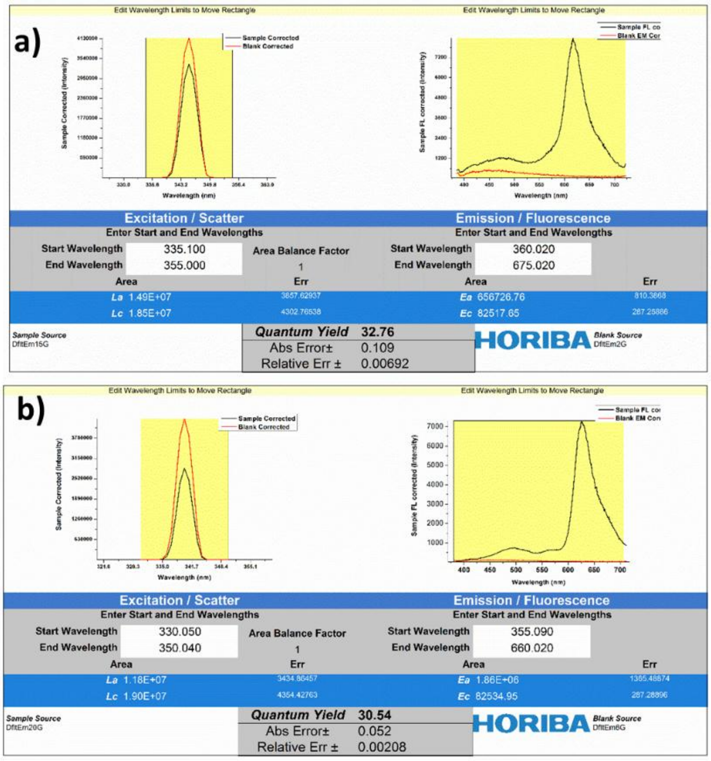

Fig. S51 Absolute fluorescence quantum yields for a) **2b** + **RhB** ([**2b**] = 10^{-5} M, [**RhB**] = 2 × 10^{-6} M) and b) **3b** + **RhB** ([**3b**] = 10^{-5} M, [**RhB**] = 2 × 10^{-6} M).

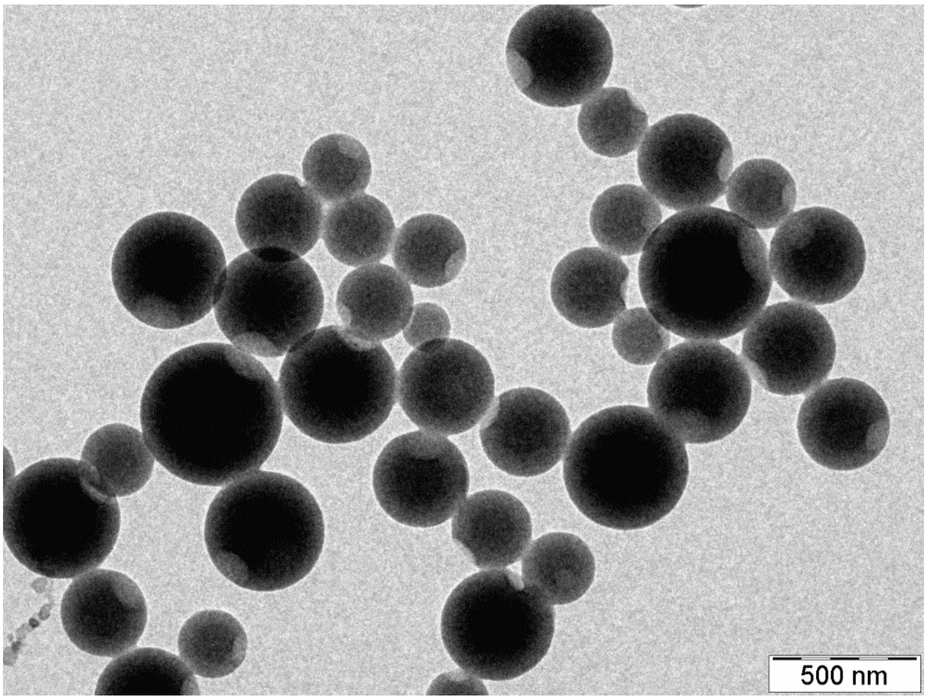

Fig. S52 TEM image showing the formation of aggregated spherical nano-aggregates of **1b** in 90% water-MeCN mixture.

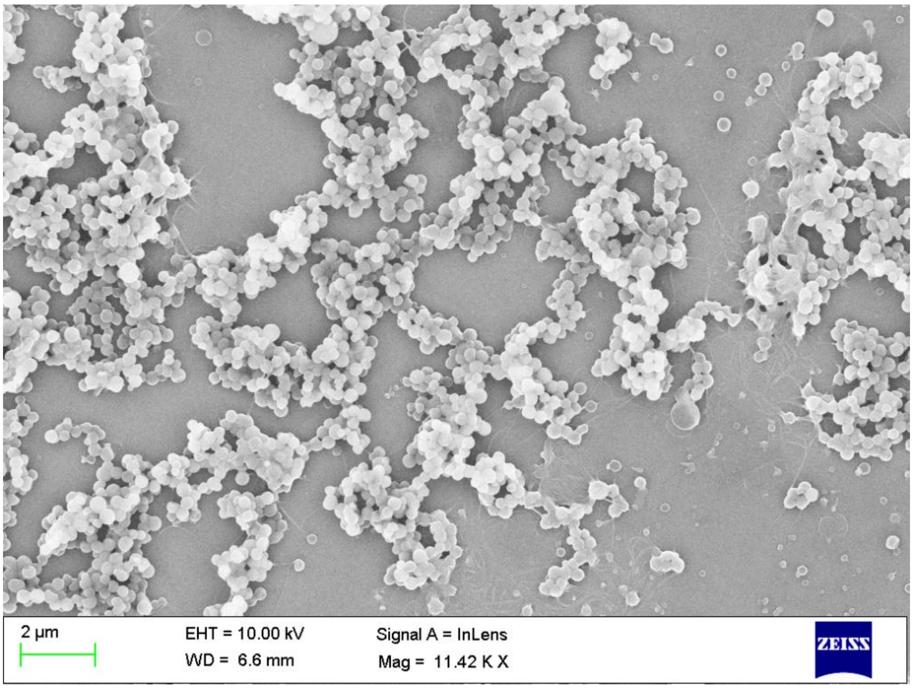

Fig. S53 SEM image showing the formation of spherical nano-aggregate of **1b** in 90% water-MeCN mixture.

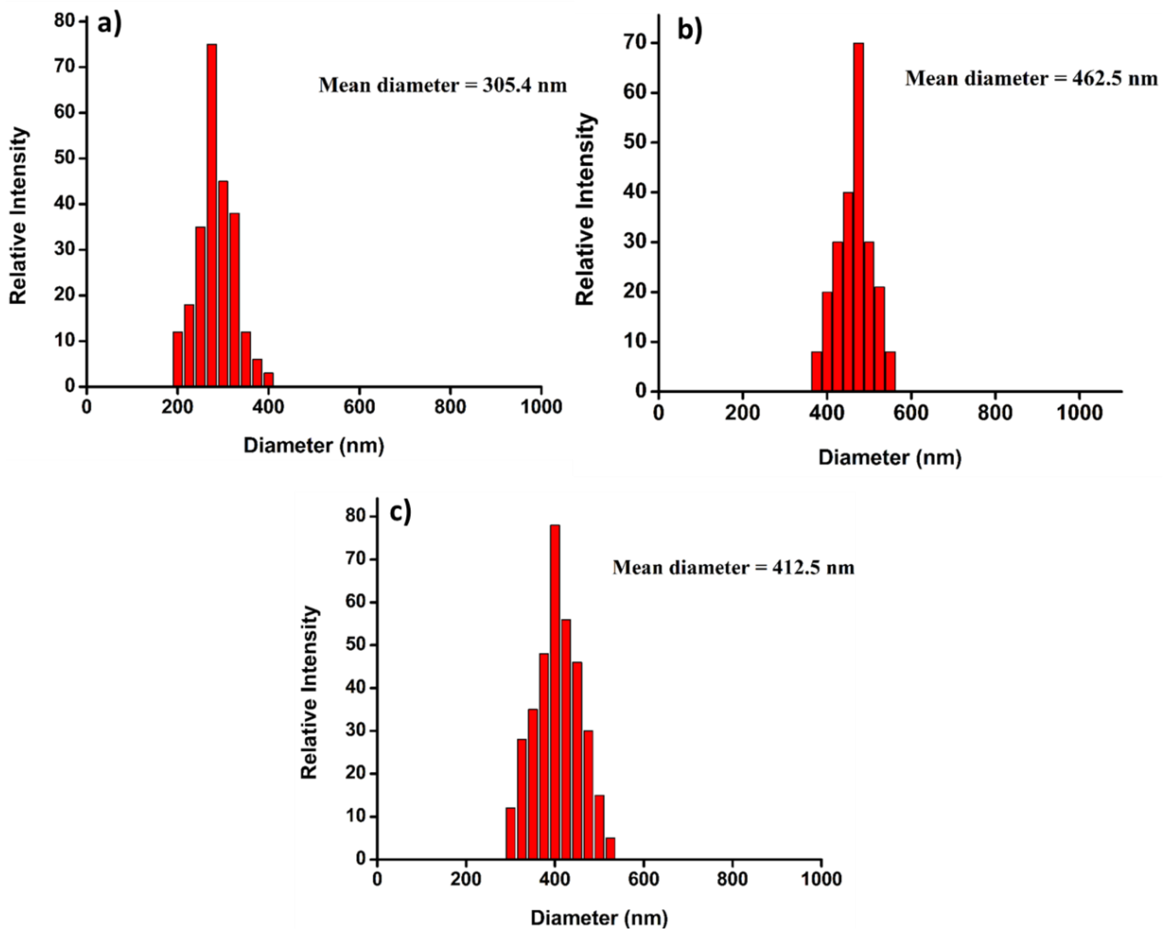

Fig. S54 DLS data profile in 90% H_2O/MeCN fraction: Size distributions patterns for a) **1b**; b) **2b** and c) **3b**.

Table S1 Photophysical data of the ligand and cages.

	Solution		Solid	
**Systems**	λ_{max}**(nm)**	$\lambda_{emission}$ **(nm)**	λ_{max} **(nm)**	$\lambda_{emission}$ **(nm)**
**L**	280 (ε_{max} = 3.02 × 10^4 molL^{-1} cm^{-1})	420	345	448
**1b**	290 (ε_{max} = 1.85 × 10^4 molL^{-1} cm^{-1}),<br>325 (ε_{max} = 3.90× 10^4 molL^{-1} cm^{-1}).	480	345	510
**2b**	284 (ε_{max} = 7.05 × 10^4 molL^{-1} cm^{-1}),<br>345 (ε_{max} = 5.28 × 10^4 molL^{-1} cm^{-1}).	540	365	555
**3b**	282 (ε_{max} = 4.92 × 10^4 molL^{-1} cm^{-1}),<br>340 (ε_{max} = 5.01 × 10^4 molL^{-1} cm^{-1}).	530	360	550

Table S2 Fluorescence emission maxima in different solvents.

	Metal complex ($\lambda_{emission}$ $in\ nm$)		
**Solvents**	**1b**	**2b**	**3b**
**Toluene**	460	495	515
**$CHCl_3$**	472	527	516
**DCM**	477	532	527
**THF**	474	531	526
**ACN**	480	538	530
**DMF**	479	538	531
**DMSO**	457	534	520

Table S3 Absolute fluorescence quantum yields of compounds under different conditions.

Quantum Yield (Φ){%}			
**Systems**	**0% water in ACN**	**90% water in ACN**	**Solid State**
**L**	3.46	15.05	23.22
**1b**	12.45	21.22	24.07
**2b**	4.63	25.67	29.34
**2b + RhB*** {[2b] = 10^{-5} M, [RhB] = 2 × 10^{-6} M}	-----	32.76	----
**3b**	6.27	22.34	26.91
**3b + RhB*** {[2b] = 10^{-5} M, [RhB] = 2 × 10^{-6} M}	------	30.54	------

*Solution of **2b+RhB** and **3b+RhB** were excited corresponding to λ_{max} for **2b** and **3b** respectively**.**

Table S4 Fluorescence lifetime parameters.

Systems	τ_1	B1	τ_2	B2	χ^2	τ_{av}
**1b**	1.09	58.37	4.23	41.63	1.09	2.36
**2b**	0.97	70.60	3.90	29.40	1.02	1.83

**2b+RhB** {[2b] = 10^{-5} M, [RhB] = 2 × 10^{-6} M}	0.74	78.96	2.98	21.04	0.98	1.20
**3b**	0.80	58.06	3.98	41.94	0.97	2.09
**3b+RhB** {[3b] = 10^{-5} M, [RhB] = 2 × 10^{-6} M}	0.70	68.71	3.21	31.29	1.05	1.47

- Bi-exponential fitting
- τ_1 and τ_2 represents life time
- B1 and B2 are real amplitude corresponding to $\boldsymbol{\tau_1}$ and $\boldsymbol{\tau_2}$ respectively.
- τ_{av} calculated via following Equation (S1):

$$\tau_{av} = \frac{(\tau_1 \times \mathrm{B1} + \tau_2 \times \mathrm{B2})}{(\mathrm{B1} + \mathrm{B2})} \qquad \text{Equation S1}$$

Energy transfer efficiency

Energy transfer efficiency is calculated by following Equation (S2):

$$\Phi_{ET} = 1 - \frac{I_{DA}}{I_D} \qquad \text{Equation S2}$$

Where

I_{DA} = Intensity of donor emission in presence of acceptor

I_D = Intensity of donor emission in absence of acceptor

Φ_{ET} = fractional energy transfer efficiency

Table S5 Energy transfer efficiency for **2b**+**RhB** and **3b**+**RhB**.

LHSs	λ_{ex}	I_{DA}	I_D	Φ_{ET}(%)
**2b**+**RhB**	340	30183	133588	77
**3b**+**RhB**	345	40704	92807	58

Antenna effect

The antenna effect calculated for certain concentration of donor and acceptor equal to the ratio of emission intensity at 610 (for 2b) {Equation S3} and 615 (for 3b) {Equation S4} upon excitation of the donor.

(a) Antenna effect for **2b**+**RhB:**

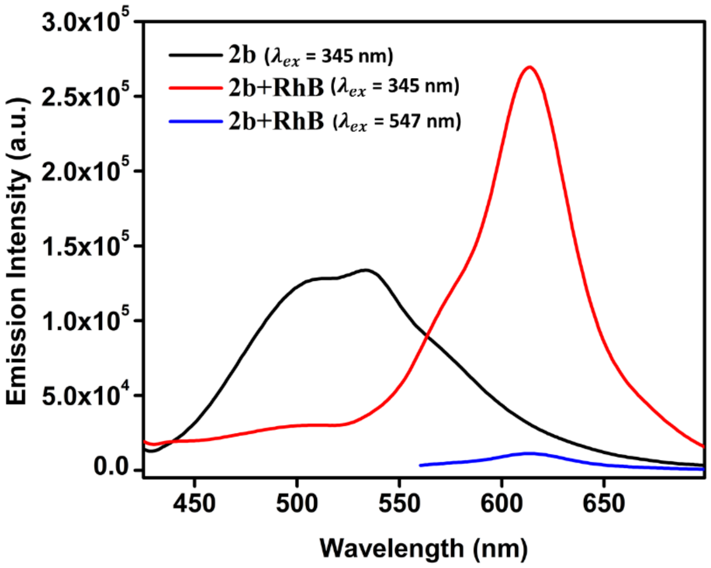

Fig. S55 Fluorescence emission intensity of **2b** ($\lambda_{ex} = 345\ nm$), **2b**+**RhB** {[**2b** = 10^{-5}M] and [**RhB**] = 2×10^{-6}M} ($\lambda_{ex} = 345\ nm$) and **2b**+**RhB** {[**2b** = 10^{-5}M] and [**RhB**] = 2×10^{-6}M} ($\lambda_{ex} = 547\ nm$).

$$\text{Antenna effect} = \frac{I^{610\ nm}_{A+D(\lambda_{ex}=345\ nm)} - I^{610\ nm}_{D(\lambda_{ex}=345\ nm)}}{I^{610\ nm}_{A+D(\lambda_{ex}=547\ nm)}} \qquad \text{Equation S3 (for } \mathbf{2b}\text{)}$$

$I^{610\ nm}_{D(\lambda_{ex}=345\ nm)}$= Emission intensity of donor at 610 nm upon excitation at 345 nm.

$I^{610\ nm}_{A+D(\lambda_{ex}=345\ nm)}$= Emission intensity of (donor+ acceptor) {[**2b** = 10^{-5}M] and [**RhB**] = 2×10^{-6} M} at 610 nm upon excitation at 345 nm.

$I^{610\ nm}_{A+D(\lambda_{ex}=547\ nm)}$= Emission intensity of (donor+ acceptor) {[**2b** = 10^{-5}M] and [**RhB**] = 2×10^{-6} M} at 610 nm upon excitation at 547 nm.

Table S6 Energy transfer efficiency for **2b**+**RhB**.

LHSs	$I^{610\ nm}_{A+D(\lambda_{ex}=345\ nm)}$	$I^{610\ nm}_{D(\lambda_{ex}=345\ nm)}$	$I^{610\ nm}_{A+D(\lambda_{ex}=547\ nm)}$	Antenna effect
**2b**+**RhB**	266740	33222	11120	21

(b) Antenna effect for **3b**+**RhB:**

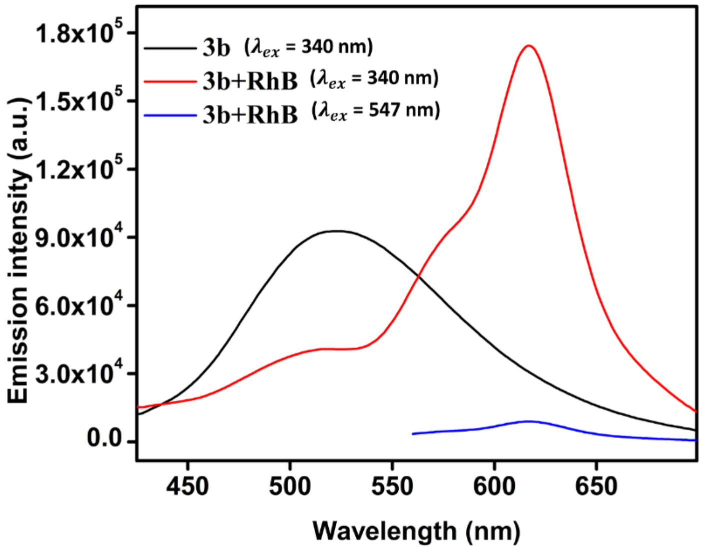

Fig. S56 Fluorescence emission intensity of **3b** ($\lambda_{ex} = 340\ nm$), **3b**+**RhB** {[**3b** = 10^{-5}M] and [**RhB**] = 2×10^{-6}M} ($\lambda_{ex} = 340\ nm$) and **3b**+**RhB** {[**3b** = 10^{-5}M] and [**RhB**] = 2×10^{-6}M} ($\lambda_{ex} = 547\ nm$).

$$\text{Antenna effect} = \frac{I^{615\ nm}_{A+D(\lambda_{ex}=340\ nm)} - I^{615\ nm}_{D(\lambda_{ex}=340\ nm)}}{I^{615\ nm}_{A+D(\lambda_{ex}=547\ nm)}}$$ Equation S4 (for **3b**)

$I^{615\ nm}_{D(\lambda_{ex}=340\ nm)}$= Emission intensity of donor (**3b**) at 615 nm upon excitation at 340 nm.

$I^{615\ nm}_{A+D(\lambda_{ex}=340\ nm)}$ = Emission intensity of (donor+ acceptor) {[**3b** = 10^{-5}M] and [**RhB**] = 2×10^{-6}M} at 615 nm upon excitation at 340 nm.

$I^{615\ nm}_{A+D(\lambda_{ex}=547\ nm)}$= Emission intensity of (donor+ acceptor) {[**3b** = 10^{-5}M] and [**RhB**] = 2×10^{-6}M} at 615 nm upon excitation at 547 nm.

Table S7 Energy transfer efficiency for **3b**+**RhB**.

LHSs	$I^{615\ nm}_{A+D(\lambda_{ex}=340\ nm)}$	$I^{615\ nm}_{D(\lambda_{ex}=340\ nm)}$	$I^{615\ nm}_{A+D(\lambda_{ex}=547\ nm)}$	Antenna effect
**3b**+**RhB**	174372	31709	8916	16

General procedure for the synthesis of 6a-6f.

In air, *N*,*N*-dimethylaniline (5 mg, 0.0412 mmol), *N*-aryl/alkyl maleimide derivatives (0.0412 mmol), catalyst cage **2b** (13.2 mg, 0.002 mmol, 5 mol%) and rhodamine B (0.2 mg, 0.0004 mmol,1 mol%) were successively added to a 0.5 mL acetonitrile (MeCN). To this solution 4.5 mL water was added to make the final solution as 90% water/ MeCN mixture.[a] Then this reaction mixture was transferred to a quartz glass vial containing a magnetic stir bar

and then placed in white light LED photoreactor and stirred for 12 h. The vial was irradiated with visible light (white light) and maintained at 25 °C (room temperature). After the completion of reaction, solvent was evaporated under reduced pressure. Then crude product was extracted with $CDCl_3$ (0.5 mL) and 1,3,5-trimethoxybenzene (6.9 mg, 0.0412 mmol) was added as the reference to calculate the conversions of the reaction based on the ^{1}H integrals. [a]For substrate **5f** reaction was performed in 70% water/MeCN mixture; [b]Crude products were purified by flash chromatography using 5-10% ethyl acetate/hexane mixture.

Synthesis of 6a:

^{1}H NMR)400 MHz, $CDCl_3$(δ: 7.47)1H, d, J = 8 Hz(, 7.35)3H, m(, 7.27)1H, d, J = 8 Hz(, 7.20)3H, m(, 6.84)1H, m), 6.69)1H, d, J = 8 Hz(, 4.09)1H, d, J = 12 Hz(, 3.55)1H, dd, J = 12, 2 Hz(, 3.48)1H, ddd, J = 12, 8, 4 Hz(, 3.07)1H, dd, J = 8, 4 Hz), 2.78 (s, 3H). ^{13}C NMR (100 MHz, $CDCl_3$) δ (ppm): 178.1, 176.2, 148.8, 132.5, 130.8, 129.4, 129.1, 128.9, 126.8, 120.2, 119.1, 113.1, 51.1, 43.9, 42.5 and 39.9. ESI MS (m/z): Calcd. For $[M+H]^+$ 293.12, found, 293.13 for $[M+H]^+$; Calcd. For $[M+Na]^+$ 315.10, found, 315.11 for $[M+Na]^+$.

Synthesis of 6b:

^{1}H NMR)400 MHz, $CDCl_3$(δ: 7.47)1H, d, J = 4 Hz(, 7.20)1H, dd, J = 4, 4 Hz(, 6.89)1H, dd, J = 4, 4 Hz(, 6.70)1H, d, J = 4 Hz), 3.97)1H, d, J = 12 Hz(, 3.47 - 3.56)3H, m(, 3.34)1H, ddd, J = 12, 8, 4 Hz(, 3.03)1H, dd, J = 8, 4 Hz), 2.79 (s, 3H), 1.13 (3H, t, J = 8 Hz). ^{13}C NMR (100 MHz, $CDCl_3$) δ (ppm): 178.9, 177.0, 148.9, 130.7, 129.0, 120.0, 119.3, 112.9, 51.5, 43.9, 42.4, 39.8, 34.7 and 13.4. ESI MS (m/z): ESI MS (m/z): Calcd. For $[M+H]^+$ 245.12, found, 245.13 for $[M+H]^+$; Calcd. For $[M+Na]^+$ 267.12, found, 267.12 for $[M+Na]^+$.

Synthesis of 6c:

^{1}H NMR)400 MHz, $CDCl_3$(δ: 7.46)1H, d, J = 4 Hz(, 7.20)1H, dd, J = 8, 8 Hz(, 6.88)1H, dd, J = 8, 8 Hz(, 6.70)1H, d, J = 8 Hz), 3.91)1H, d, J = 12 Hz(, 3.91)1H, d, J = 12 Hz(, 3.45 (1H, d, J = 12 Hz), 3.28)1H, ddd, J = 12, 8, 4 Hz), 3.17 (1H, s) 3.03)1H, dd, J = 12, 4 Hz), 2.79 (s, 3H), 2.04 - 2.10 (2H, m), 1.77 (2H, d, J = 12 Hz), 1.53 – 1.61 (4H, m), 1.21 – 1.26 (m, 2H). ^{13}C NMR (100 MHz, $CDCl_3$) δ (ppm): 179.2, 177.3, 148.9, 130.7, 128.9, 119.9, 119.6, 112.8, 52.6, 51.3, 43.5, 42.2, 39.8, 29.3, 26.2 and 25.2. ESI MS (m/z): Calcd. For $[M+H]^+$ 299.17, found, 299.18 for $[M+H]^+$; Calcd. For $[M+Na]^+$ 321.17, found, 321.16 for $[M+Na]^+$.

Synthesis of 6d:

^{1}H NMR)400 MHz, $CDCl_3$(δ: 7.45)1H, d, J = 8 Hz(, 7.15)3H, m(, 7.03 (2H, m), 6.82)1H, m(, 6.86)1H, d, J = 8 Hz), 4.06)1H, d, J = 8 Hz(, 3.52)1H, dd, J = 12, 4 Hz(, 3.44)1H, ddd, J = 12, 8, 4 Hz(, 3.03)1H, dd, J = 8, 4 Hz), 2.76 (s, 3H), 2.27 (s, 3H) . ^{13}C NMR (100 MHz, $CDCl_3$) δ (ppm): 178.3, 176.4, 149.0, 139.0, 130.8, 130.1, 129.9, 129.1, 126.7, 120.1, 119.2, 113.0, 51.2, 44.0, 42.6, 39.9 and 21.7. ESI MS (m/z): Calcd. For $[M+H]^+$ 307.14, found, 307.15 for $[M+H]^+$; Calcd. For $[M+Na]^+$ 329.12, found, 329.13 for $[M+Na]^+$.

Synthesis of 6e:

^{1}H NMR)400 MHz, $CDCl_3$(δ: 7.43 – 7.48)3H, m(, 7.18)1H, d, J = 8 Hz(, 7.10 – 7.15 (2H, m), 6.84)1H, t, J = 8 Hz(, 6.67)1H, d, J = 8 Hz), 4.08)1H, d, J = 12 Hz(, 3.53)1H, dd, J = 12, 4 Hz(, 3.46)1H, ddd, J = 12, 8, 4 Hz(, 3.04)1H, dd, J = 8, 4 Hz), 2.76 (s, 3H). ^{13}C NMR (100 MHz, $CDCl_3$) δ (ppm): 177.8, 175.9, 149.0, 132.6, 131.5, 130.8, 129.3, 128.3, 122.7, 120.2, 118.8, 113.1, 51.1, 44.1, 42.6 and 39.9. ESI MS (m/z): Calcd. For $[M+H]^+$ 371.04, found, 371.04 for $[M+H]^+$.

Synthesis of 6f:

^{1}H NMR)400 MHz, $CDCl_3$(δ: 7.82 – 7.97)5H, m(, 7.27 - 7.46)4H, m(, 7.14 -7.18)2H, m), 6.88 (1H, t, J = 8Hz), 6.66)1H, m), 4.07)1H, d, J = 8 Hz(, 3.52)1H, dd, J = 12, 4 Hz(, 3.45)1H, ddd, J = 12, 8, 4 Hz(, 3.04)1H, dd, J = 8, 4 Hz), 2.76 (s, 3H). ^{13}C NMR (100 MHz, CD_3CN) δ (ppm): 178.7, 176.8, 133.2, 132.8, 130.8, 129.5, 129.0, 128.9, 128.2, 127.3, 126.9, 126.6, 125.7, 124.1, 123.6, 123.3, 121.5, 119.7, 117.8, 114.1, 113.0, 51.4, 44.1, 42.7 and 39.4. ESI MS (m/z): Calcd. For $[M+Na]^+$ 439.09, found, 439.08 for $[M+Na]^+$.

Recyclability studies of catalyst 2a

In air, *N*,*N*-dimethylaniline (5.0 mg, 0.0412 mmol), *N*-phenyl maleimide **5a** (7.7 mg, 0.0412 mmol), catalyst cage **2b** (13.2 mg, 0.002 mmol, 5 mol%) and rhodamine B (0.2 mg, 0.0004 mmol,1 mol%) were successively added to 0.5 mL acetonitrile (MeCN). To this solution 4.5 mL water was added to make the final solution 90% water/ MeCN mixture*. Then this reaction mixture was transferred to a quartz glass vial containing a magnetic stir bar and then placed in white light LED photoreactor and magnetically stirred for 12 h. The vial was irradiated with visible light (white light) and maintained at 25 °C (room temperature). After the completion of reaction, solvent was evaporated under reduced pressure. Then crude product was extracted with $CDCl_3$ and ^{1}H NMR checked with internal standard 1,3,5-trimethoxy benzene. Then solid cage **2b** was washed three times with $CHCl_3$ to remove Rhodamine-B dye and added fresh 1 mol% RhB and used for next run.

Detection of H_2O_2: Formation of H_2O_2 as by-product during catalytic reaction was confirmed by adding the reaction mixture **B** to the KI/starch solution which turns the colorless solution of (KI/starch) to dark blue due to oxidation of potassium iodide by hydrogen peroxide (Table S8)[S6].

Table S8 Detection of H_2O_2 by KI/starch indicator

**Solution under visible light**	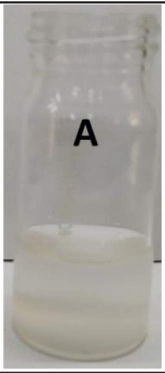	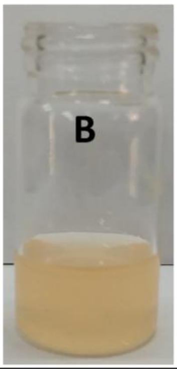	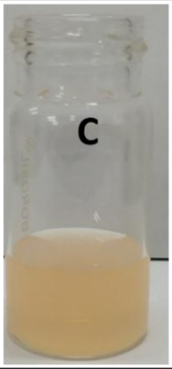	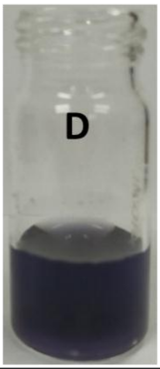	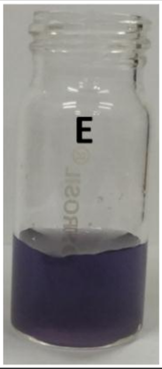
Solution	Starch +KI solution as an indicator in water[a]	Reaction mixture[b] at t = 0 h	Reaction mixture[b] at t = 12 h	Addition of 0.5 ml reaction mixture[b] at t = 12 h to solution **A**	Addition of 0.5 ml 30% H_2O_2 solution to solution **A**
Color	Colorless	Light orange	Light orange	Dark blue	Blue-violet

[a]Solution of Starch (0.04 mmol) +KI (0.04 mmol) Solution in 5 ml water; [b]Reaction mixture: N,N-Dimethylaniline **4** (0.04 mmol), N-alkyl/aryl maleimides **5** (0.04 mmol), catalyst **A** (5 mol%), **RhB** (1 mol%) in water (4.5 mL) + MeCN (0.5 ml) mixture.

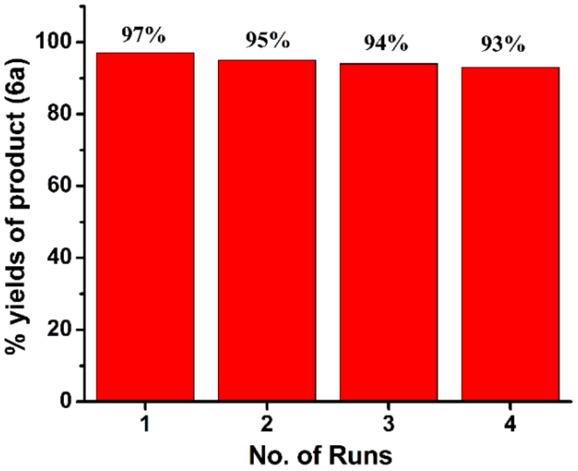

Fig. S57 Reusability studies of **2b** catalyst for the synthesis of **6a**.

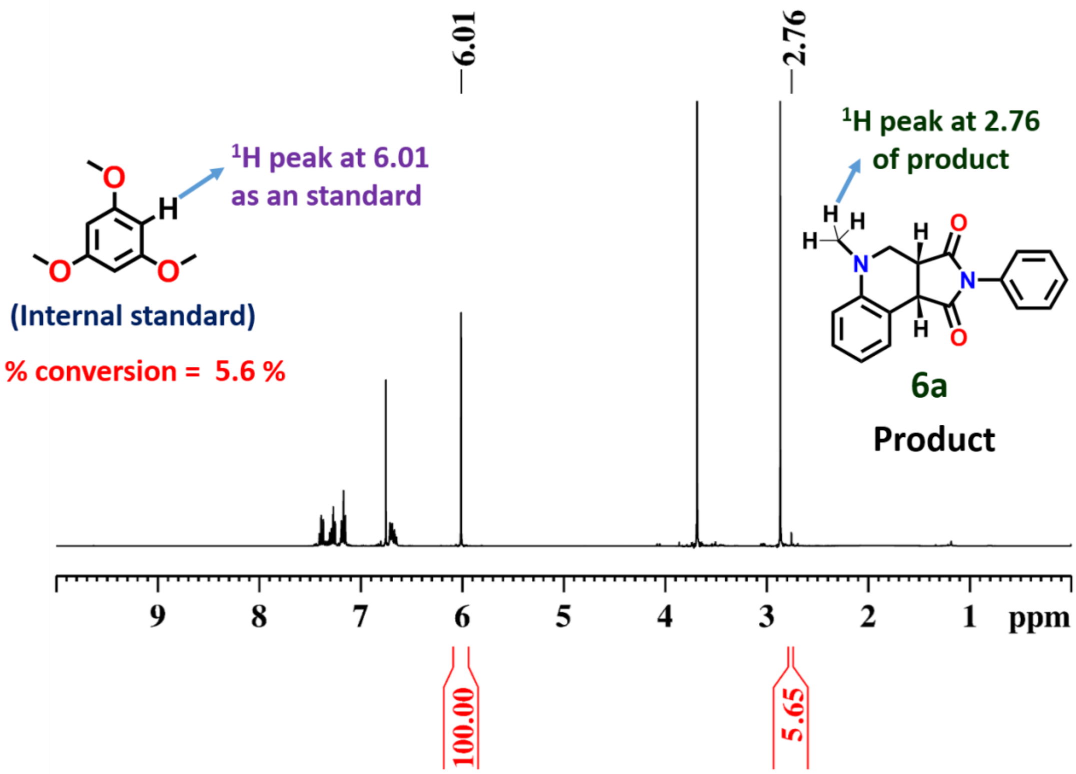

Fig. S58 ^{1}H NMR (400 MHz, $CDCl_3$) of the reaction of (**4** + **5a**) using {1 mol% **RhB**} as the photocatalyst for 12 h under visible light.

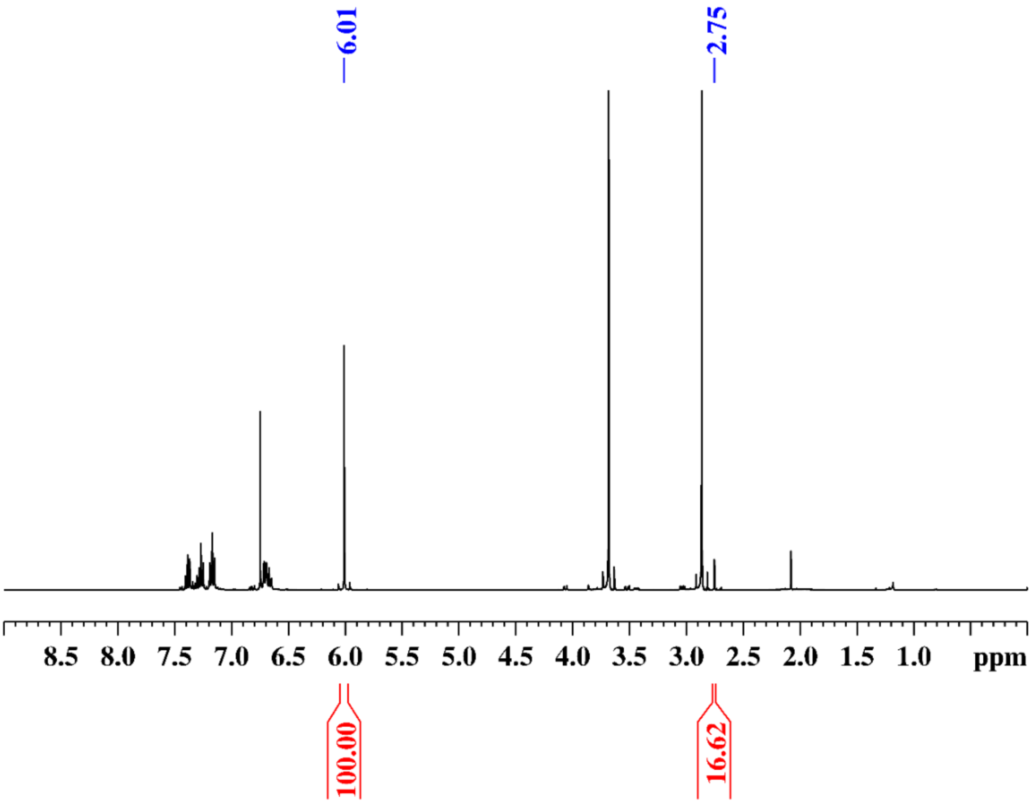

Fig. S59 ^{1}H NMR (400 MHz, $CDCl_3$) of the reaction of (**4** + **5a**) using {5 mol% **RhB**} as the photocatalyst at 12 h under visible light.

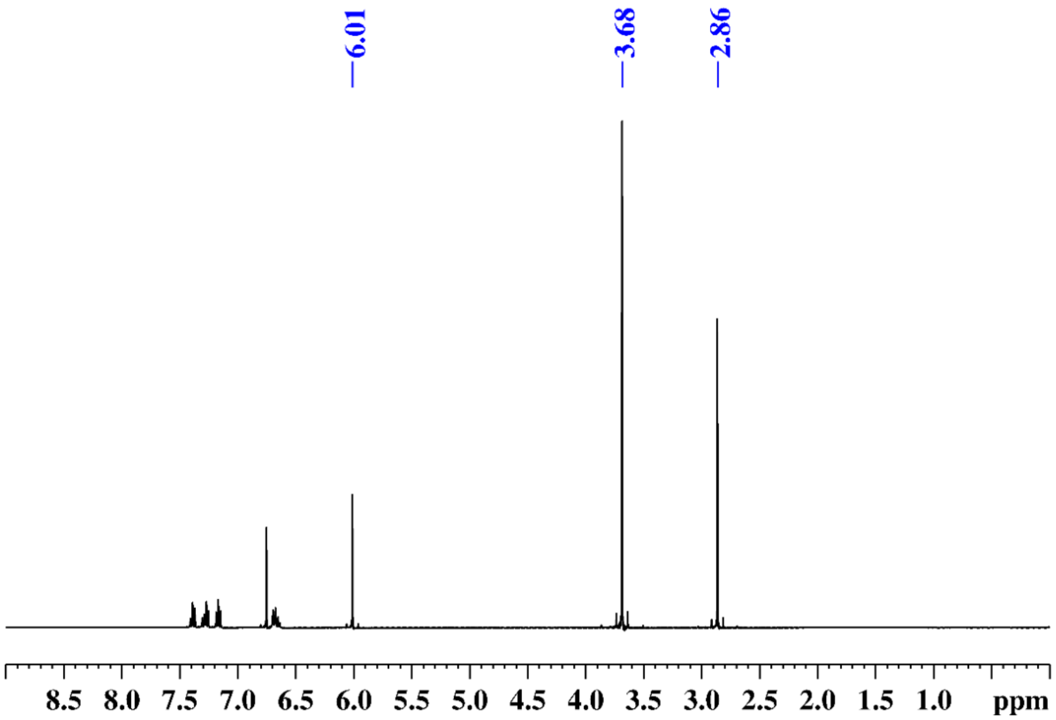

Fig. S60 ^{1}H NMR (400 MHz, $CDCl_3$) of the reaction of (**4** + **5a**) using {5 mol% cage **2b**} as the photocatalyst at 12 h under visible light.

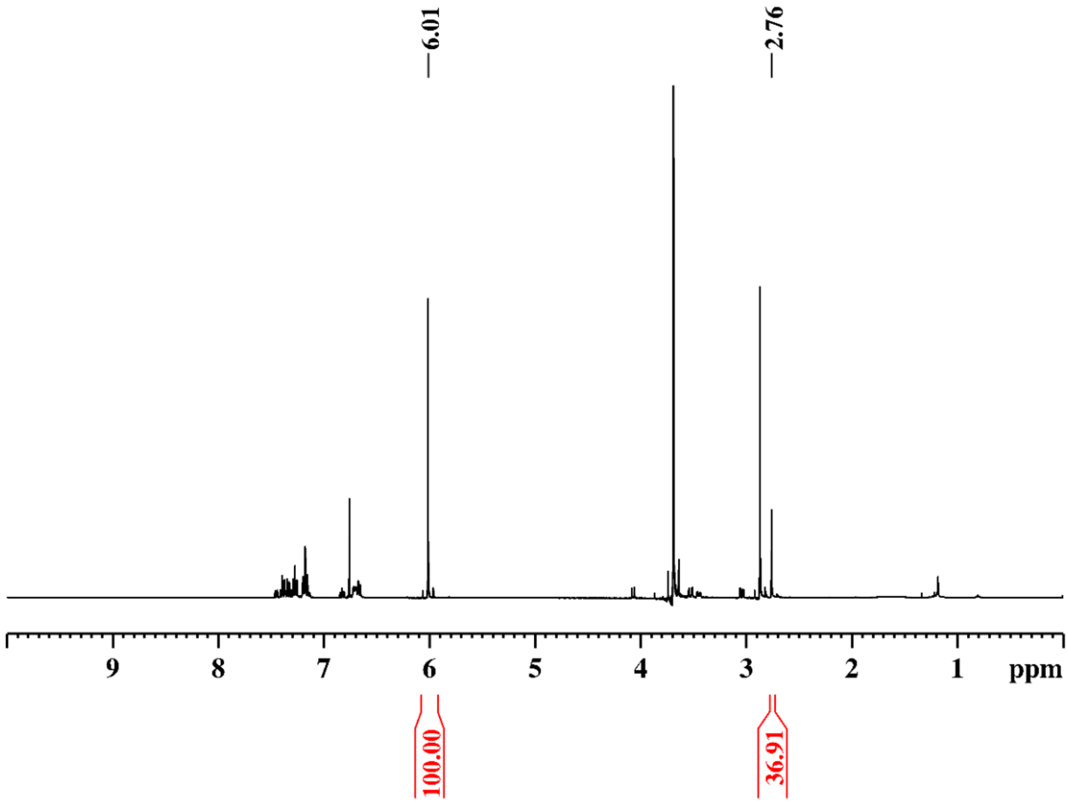

Fig. S61 ^{1}H NMR (400 MHz, $CDCl_3$) of the reaction of (**4** + **5a)** using {5 mol% cage **2b** + 1 mol% **RhB}** as the photocatalyst for 4 h under visible light.

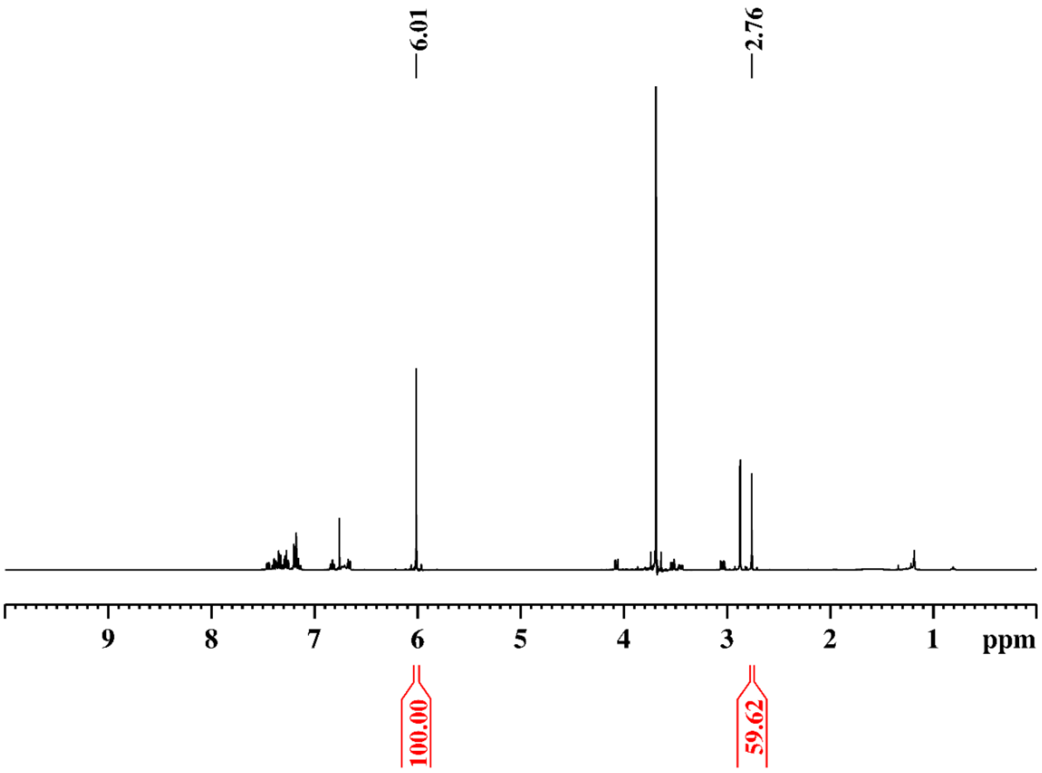

Fig. S62 ^{1}H NMR (400 MHz, $CDCl_3$) of the reaction of (**4** + **5a**) using {5 mol% cage **2b** + 1 mol% **RhB}** as the photocatalyst for 8 h under visible light.

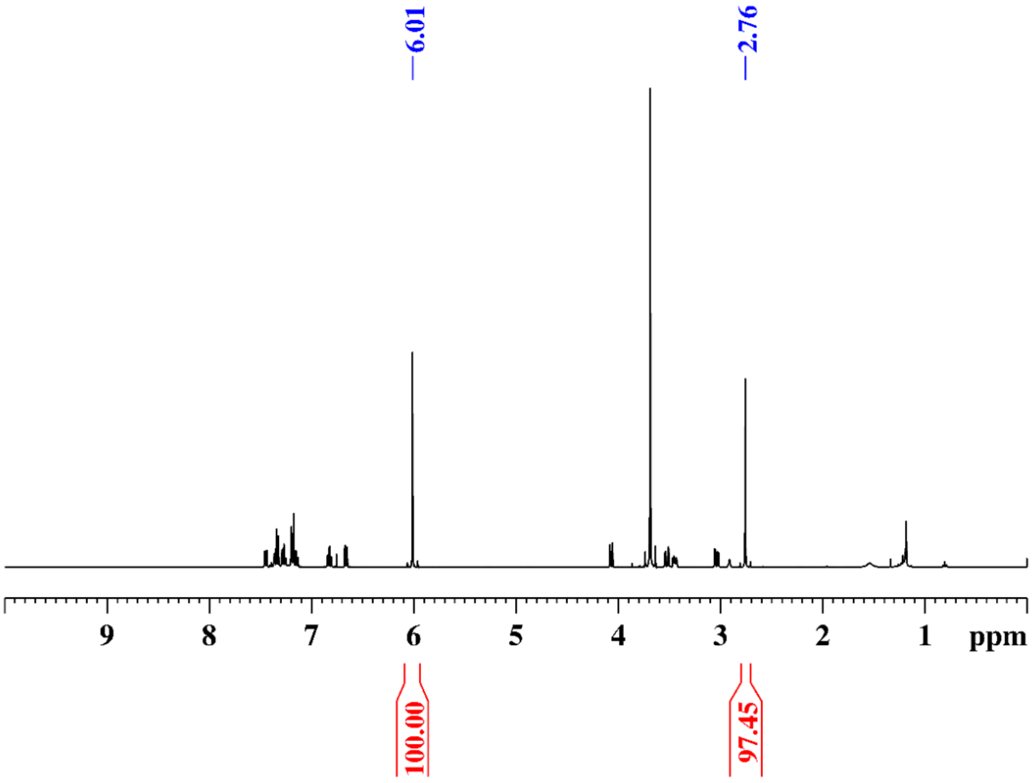

Fig. S63 ^{1}H NMR (400 MHz, $CDCl_3$) of the reaction of (**4** + **5a**) using {5 mol% cage **2b** + 1 mol% **RhB}** as the photocatalyst for 12 h under visible light.

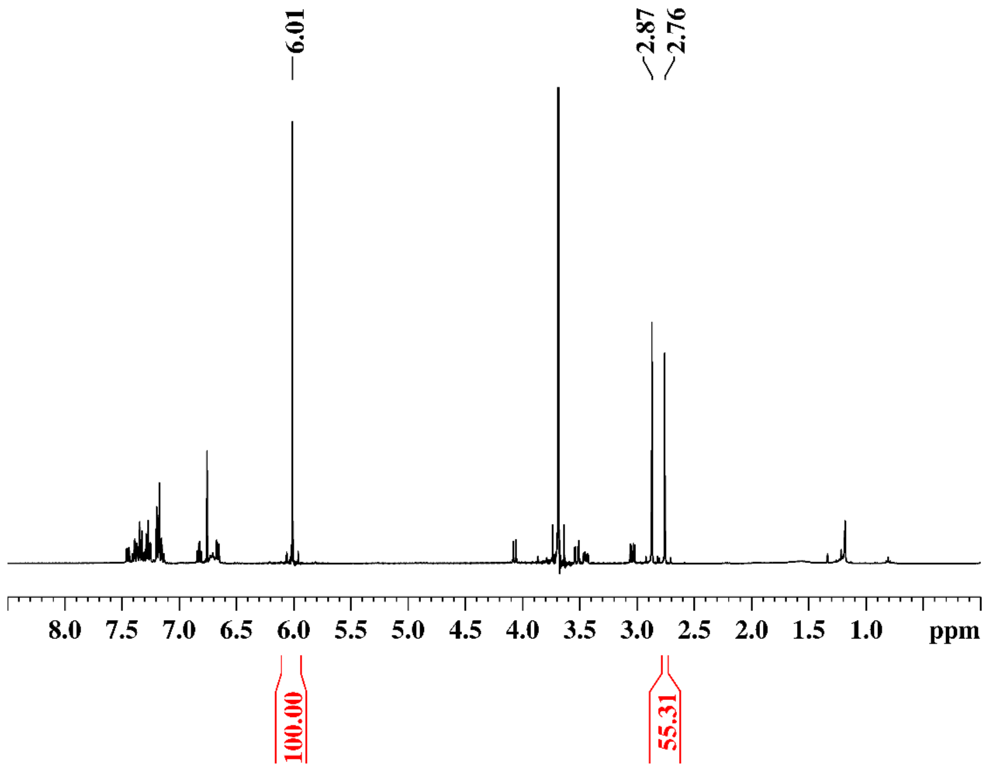

Fig. S64 ^{1}H NMR (400 MHz, $CDCl_3$) of the reaction of (**4** + **5a**) using {2.5 mol% cage **2b** + 0.5 mol% **RhB}** as the photocatalyst for 12 h under visible light.

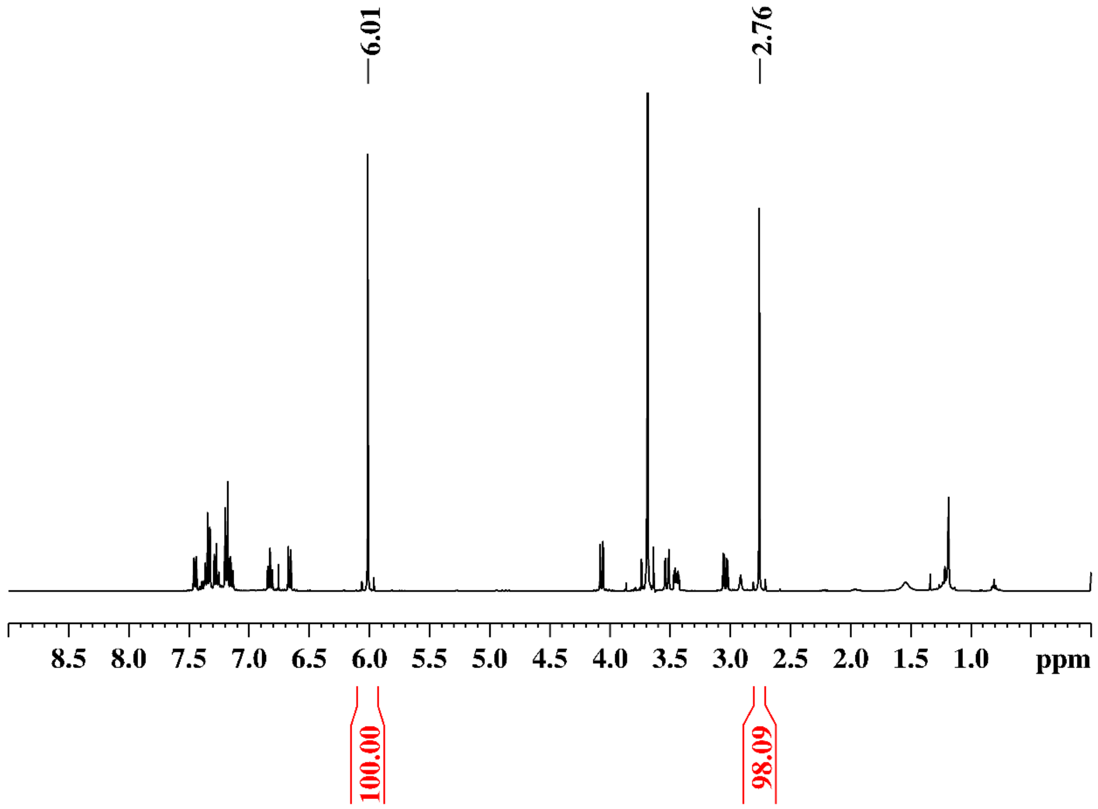

Fig. S65 ^{1}H NMR (400 MHz, $CDCl_3$) of the reaction of (**4** + **5a**) using {7.5 mol% cage **2b** + 1.5 mol% **RhB}** as the photocatalyst for 12 h under visible light.

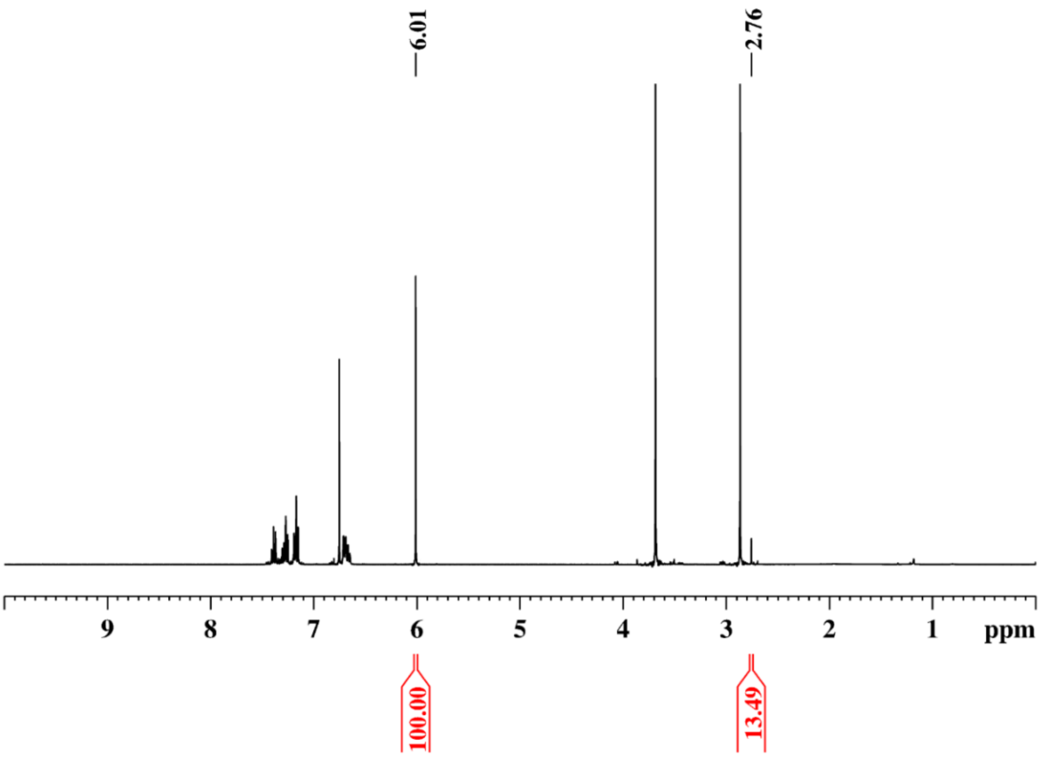

Fig. S66 ^{1}H NMR (400 MHz, $CDCl_3$) of the reaction of (**4** + **5a**) using {5 mol% cage **2b** + 1 mol% **RhB}** as the photocatalyst for12 h under dark condition.

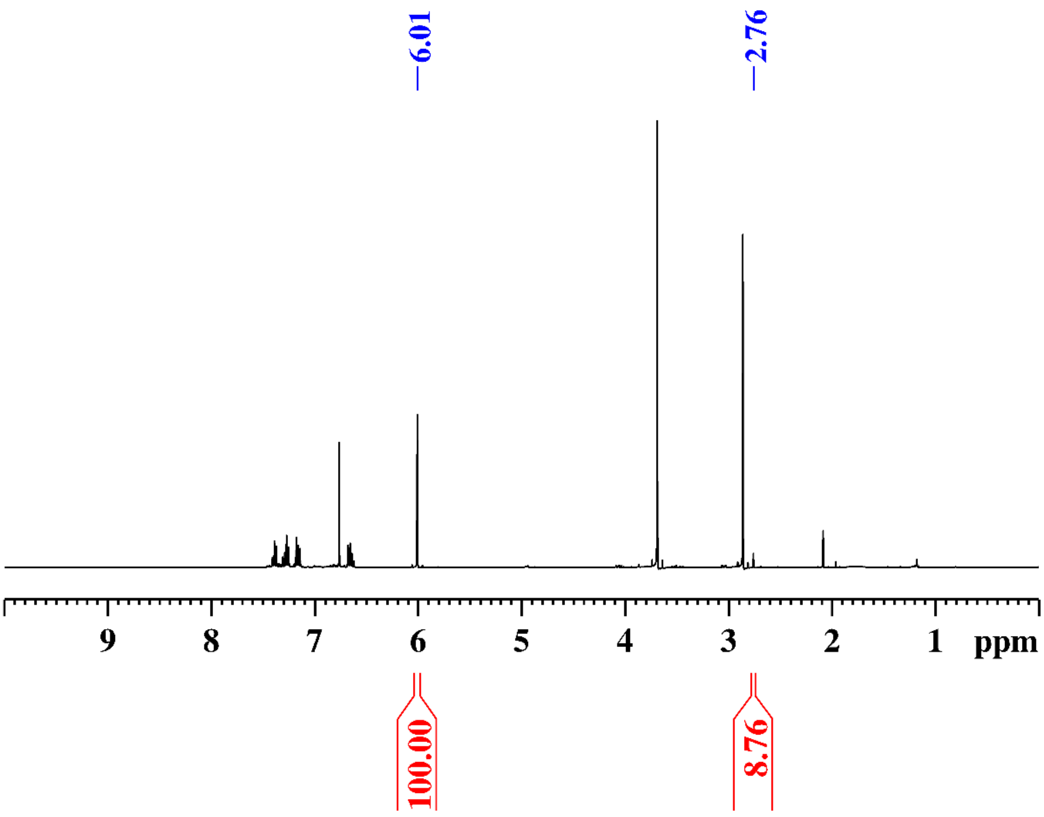

Fig. S67 ^{1}H NMR (400 MHz, $CDCl_3$) of the reaction of (**4** + **5a**) using {5 mol% ligand **L** + 1 mol% **RhB}** as the photocatalyst for12 h under visible light.

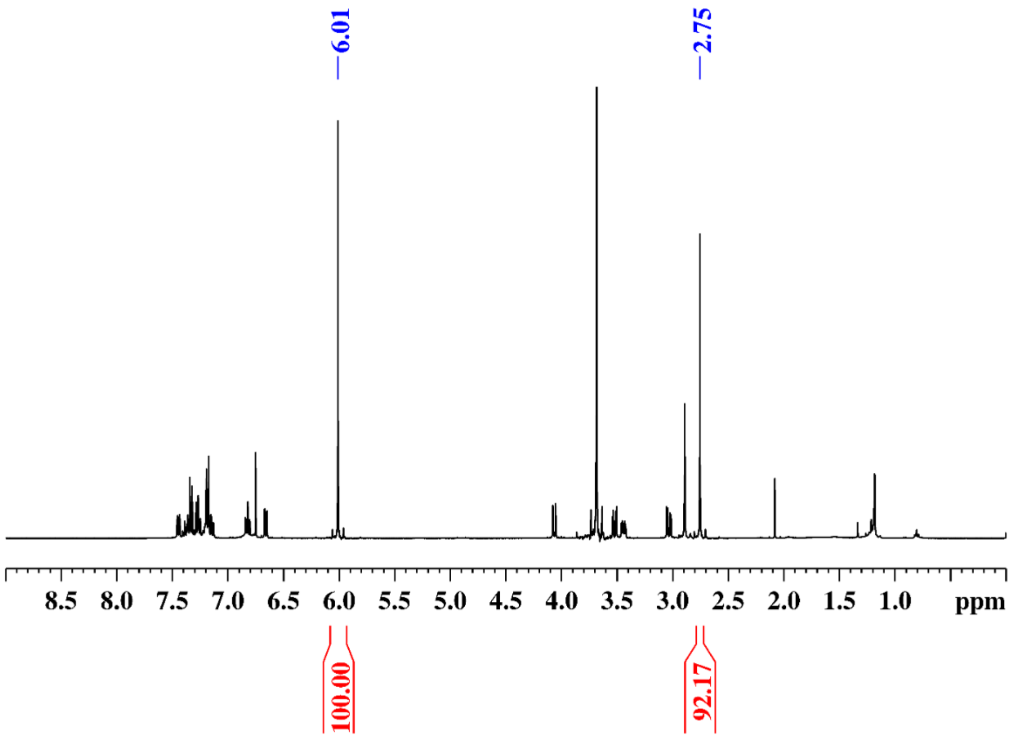

Fig. S68 ^{1}H NMR (400 MHz, $CDCl_3$) of the reaction of (**4** + **5a**) using {5 mol% cage **3b** + 1 mol% **RhB}** as the photocatalyst for 12 h under visible light.

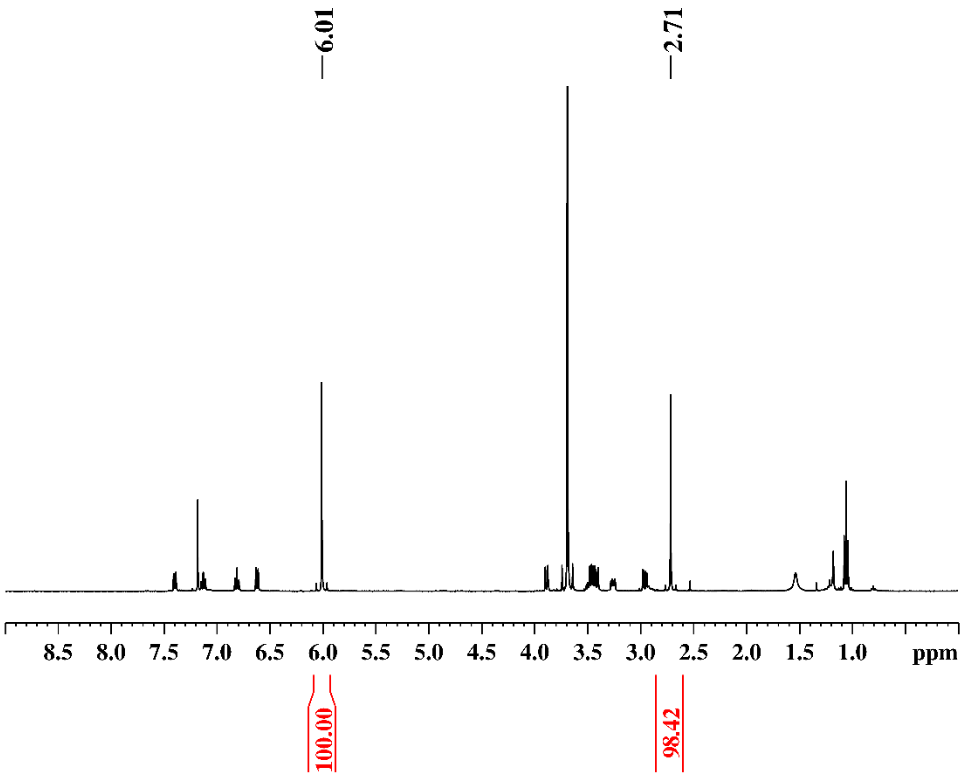

Fig. S69 ^{1}H NMR (400 MHz, $CDCl_3$) of the reaction of (**4** + **5b**) using {5 mol% cage **2b** + 1 mol% **RhB}** as the photocatalyst for 12 h under visible light.

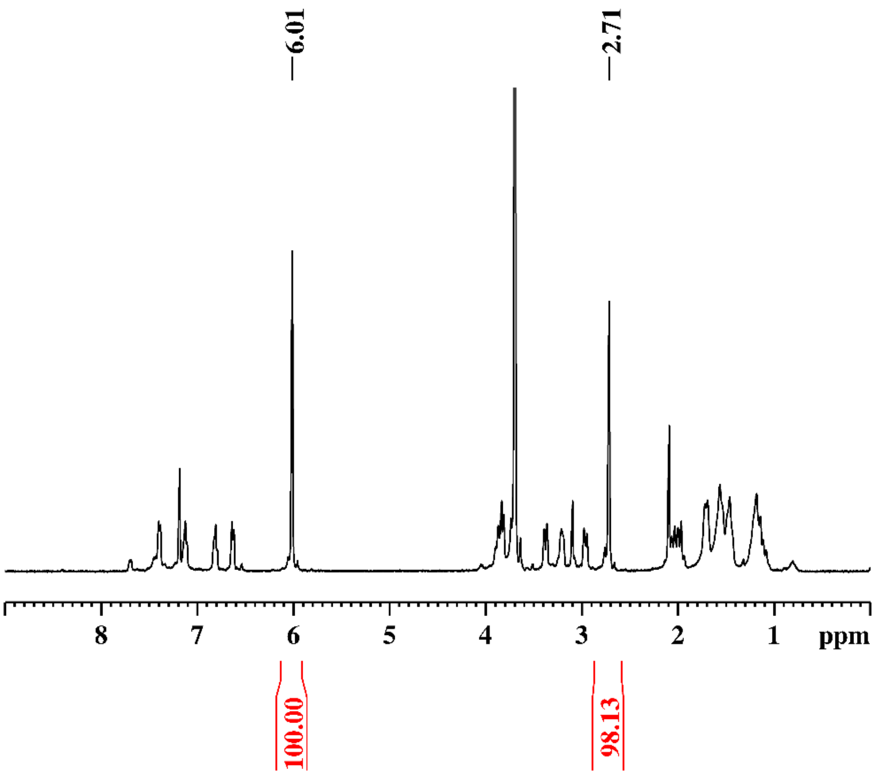

Fig. S70 ^{1}H NMR (400 MHz, $CDCl_3$) of the reaction of (**4** + **5c**) using {5 mol% cage **2b** + 1 mol% **RhB**} as the photocatalyst for 12 h under visible light.

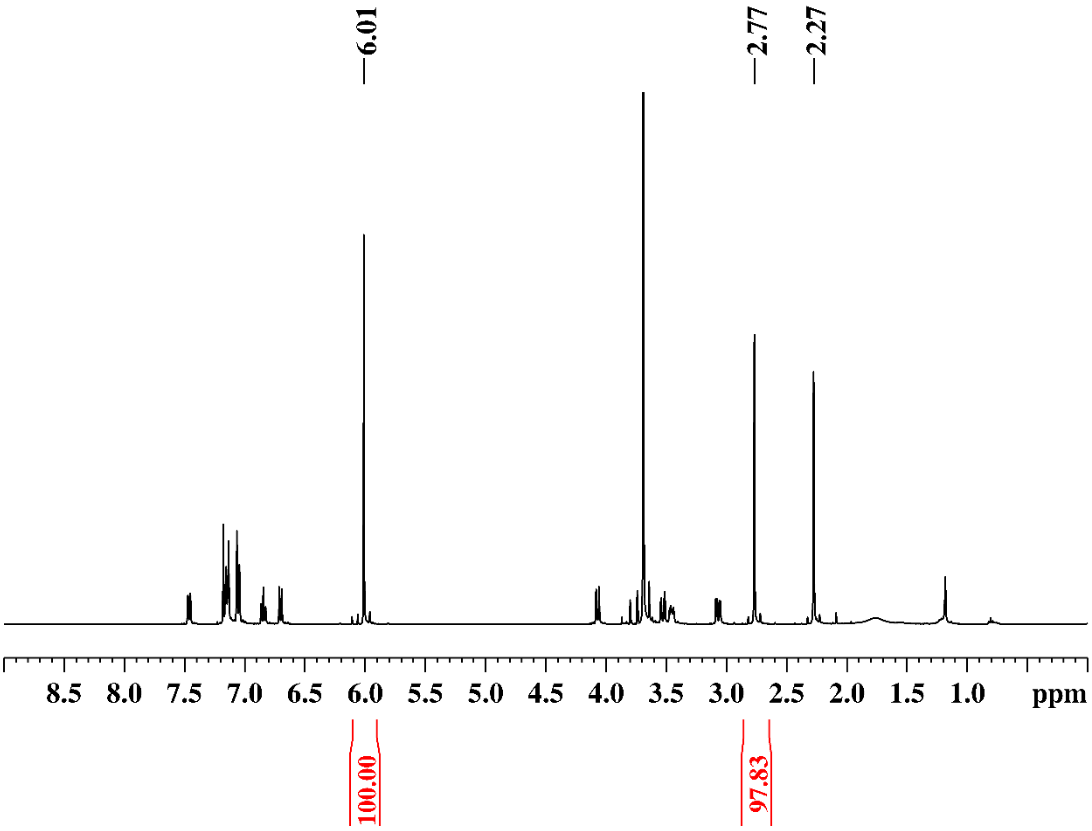

Fig. S71 ^{1}H NMR (400 MHz, $CDCl_3$) of the reaction of (**4** + **5d**) using {5 mol% cage **2b** + 1 mol% **RhB**} as the photocatalyst for 12 h under visible light.

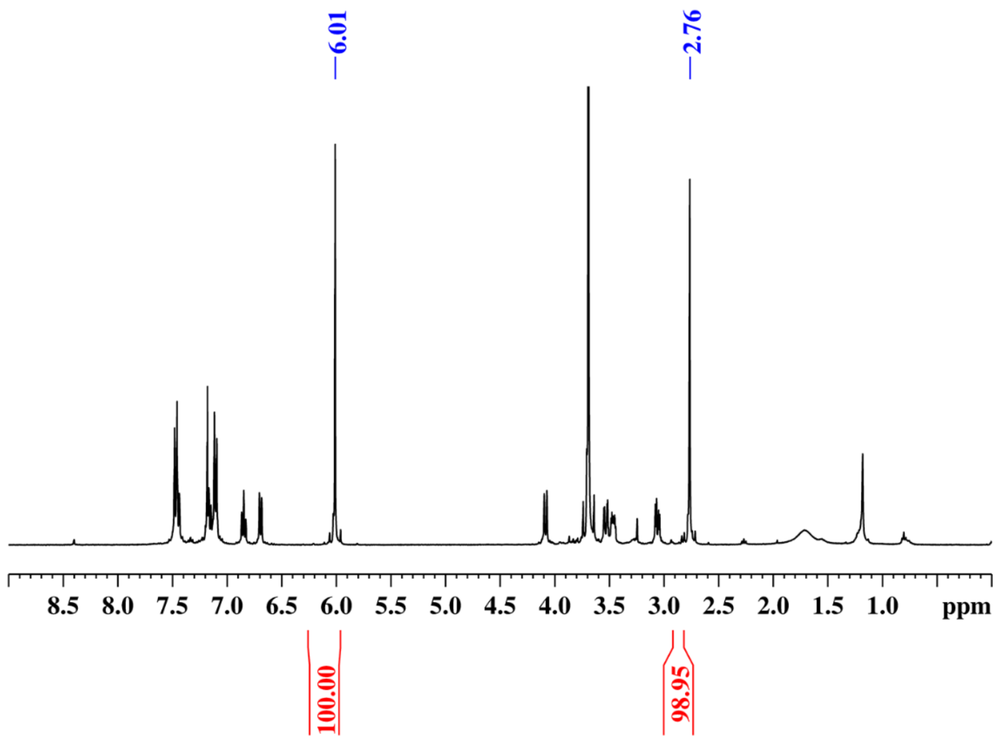

Fig. S72 ^{1}H NMR (400 MHz, $CDCl_3$) of the reaction of (**4** + **5e**) using {5 mol% cage **2b** + 1 mol% **RhB}** as the photocatalyst for 12 h under visible light.

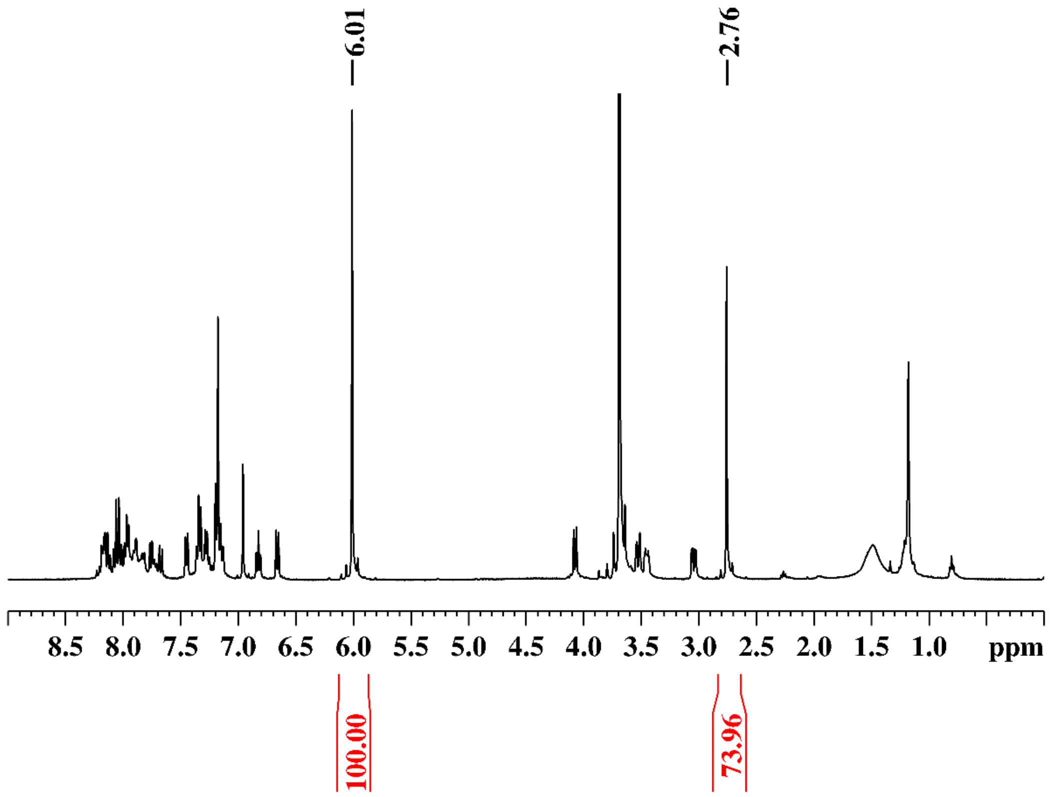

Fig. S73 ^{1}H NMR (400 MHz, $CDCl_3$) of the reaction of (**4** + **5f**) using {5 mol% cage **2b** + 1 mol% **RhB}** as the photocatalyst for 12 h under visible light in 70% water/MeCN mixture.

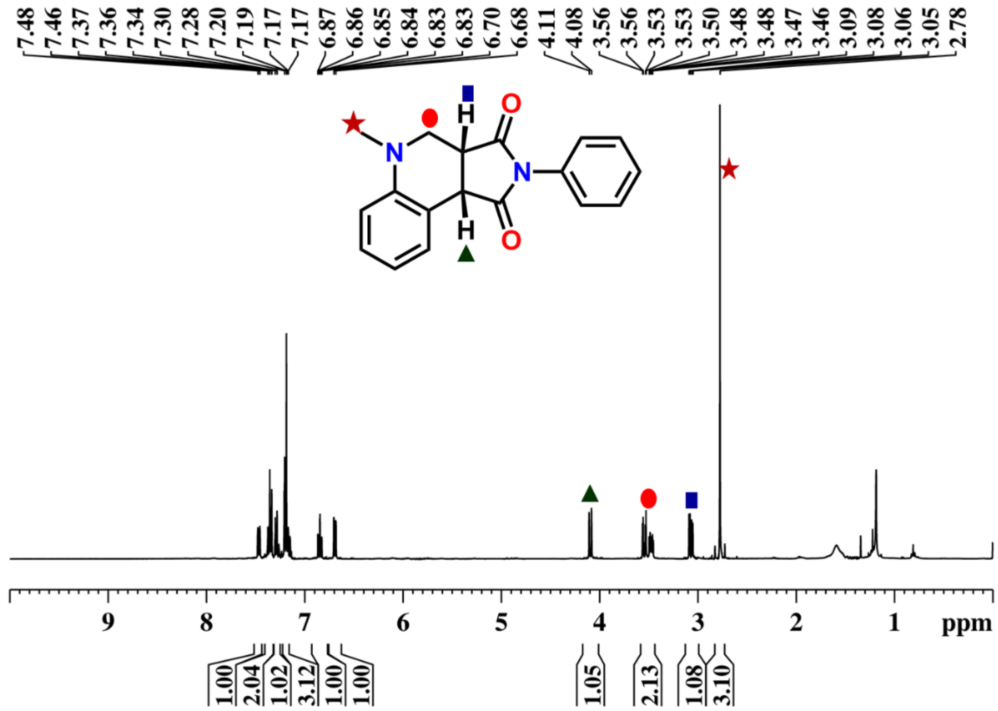

Fig. S74 ^{1}H NMR ($CDCl_3$, 400 MHz) of **6a**.

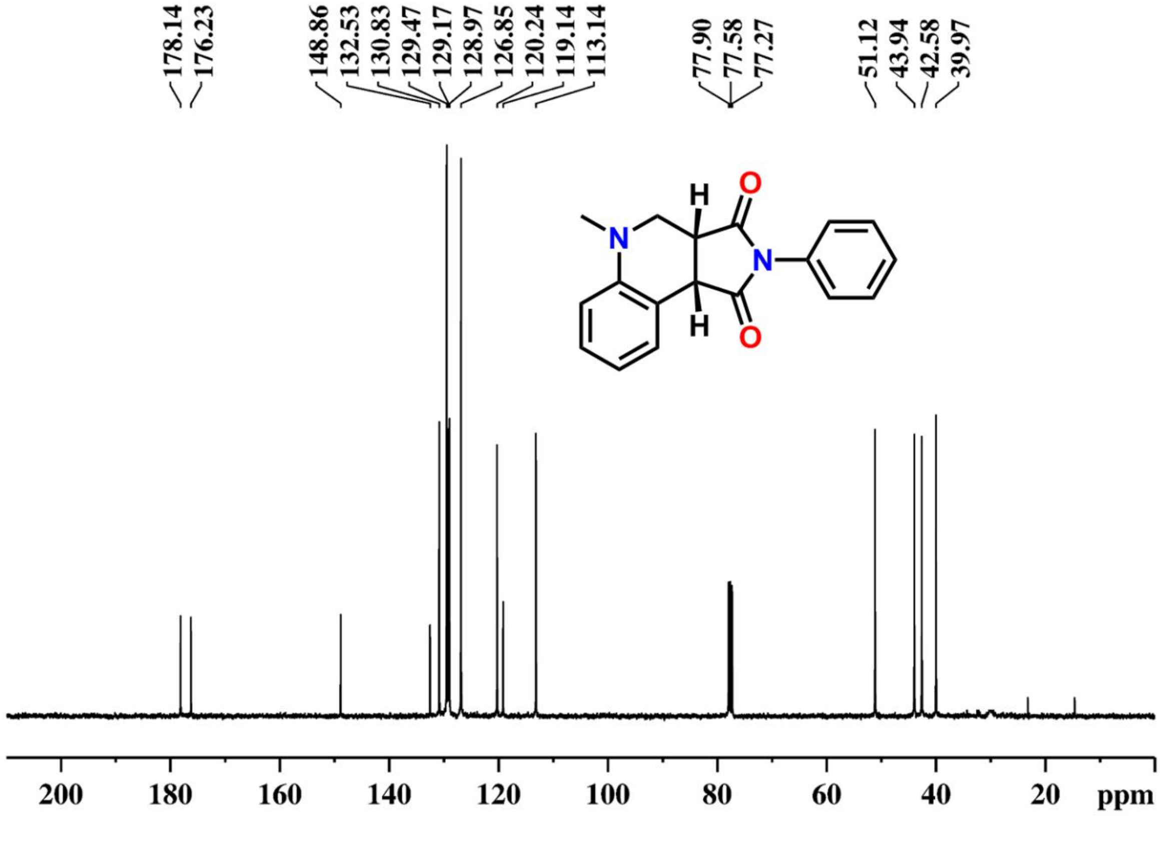

Fig. S75 ^{13}C NMR ($CDCl_3$, 100 MHz) of **6a**.

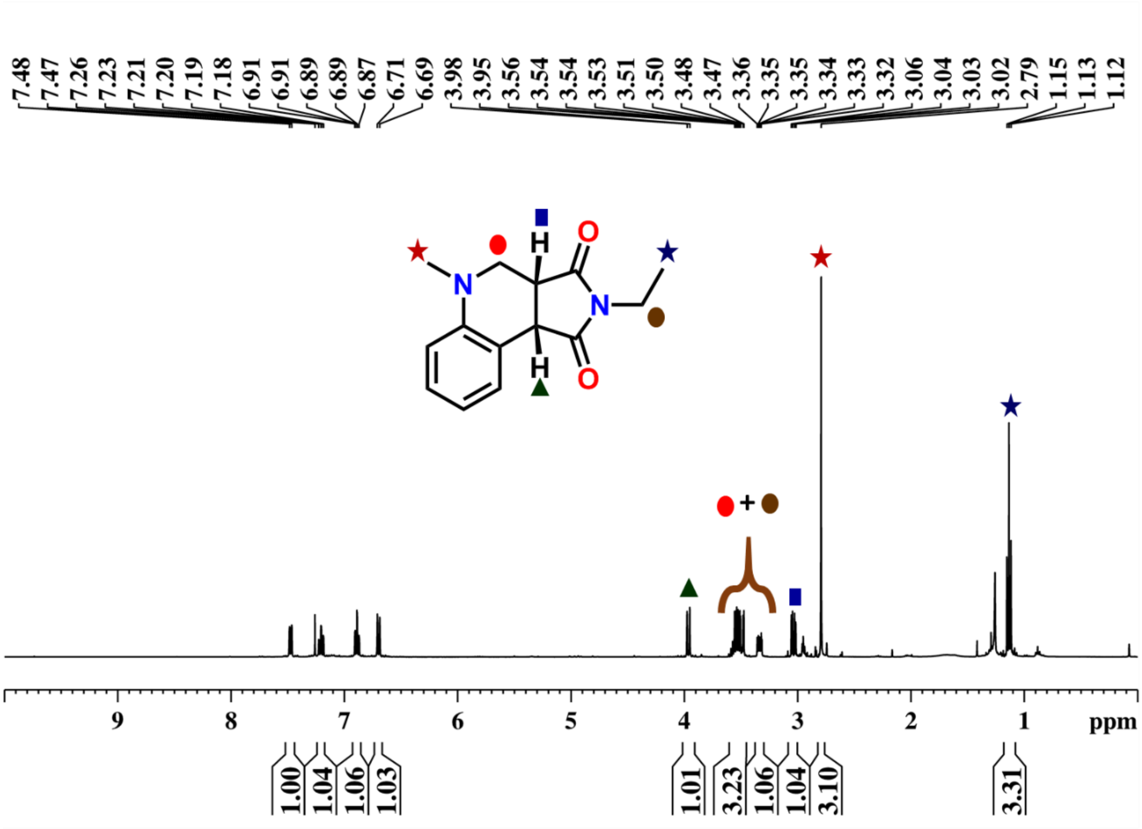

Fig. S76 ^{1}H NMR ($CDCl_3$, 400 MHz) of **6b**.

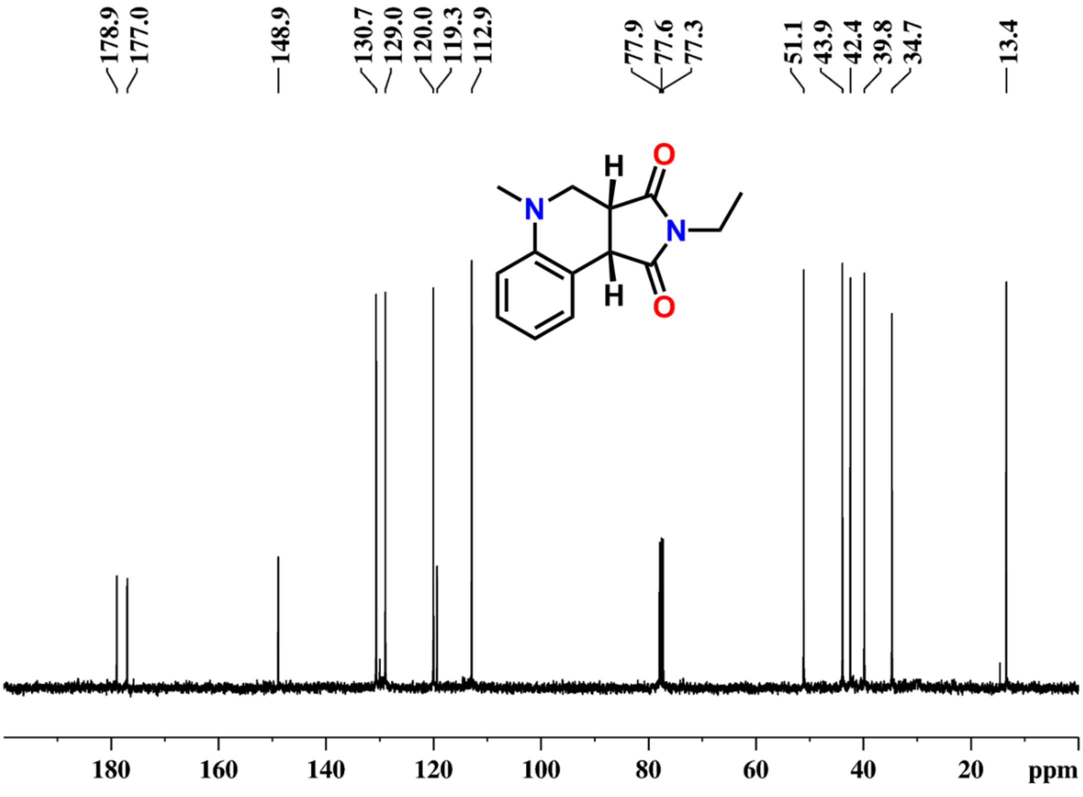

Fig. S77 ^{13}C NMR ($CDCl_3$, 100 MHz) of **6b**.

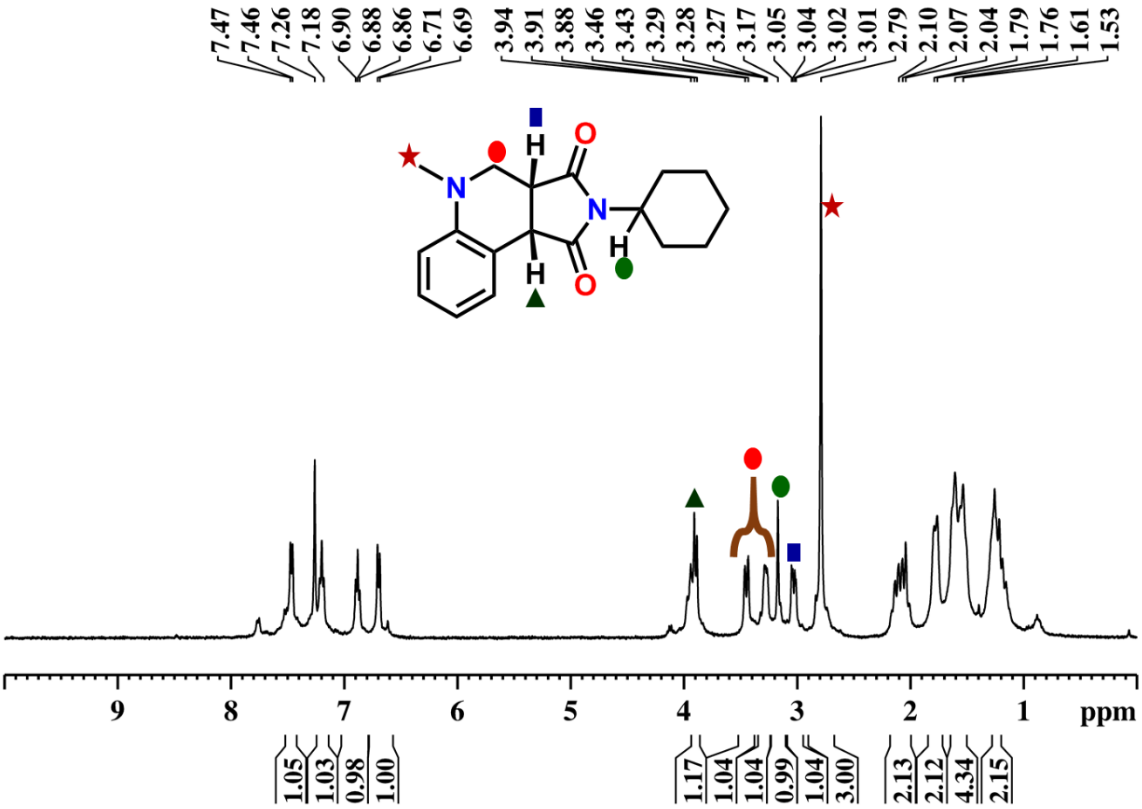

Fig. S78 ^{1}H NMR ($CDCl_3$, 400 MHz) of **6c**.

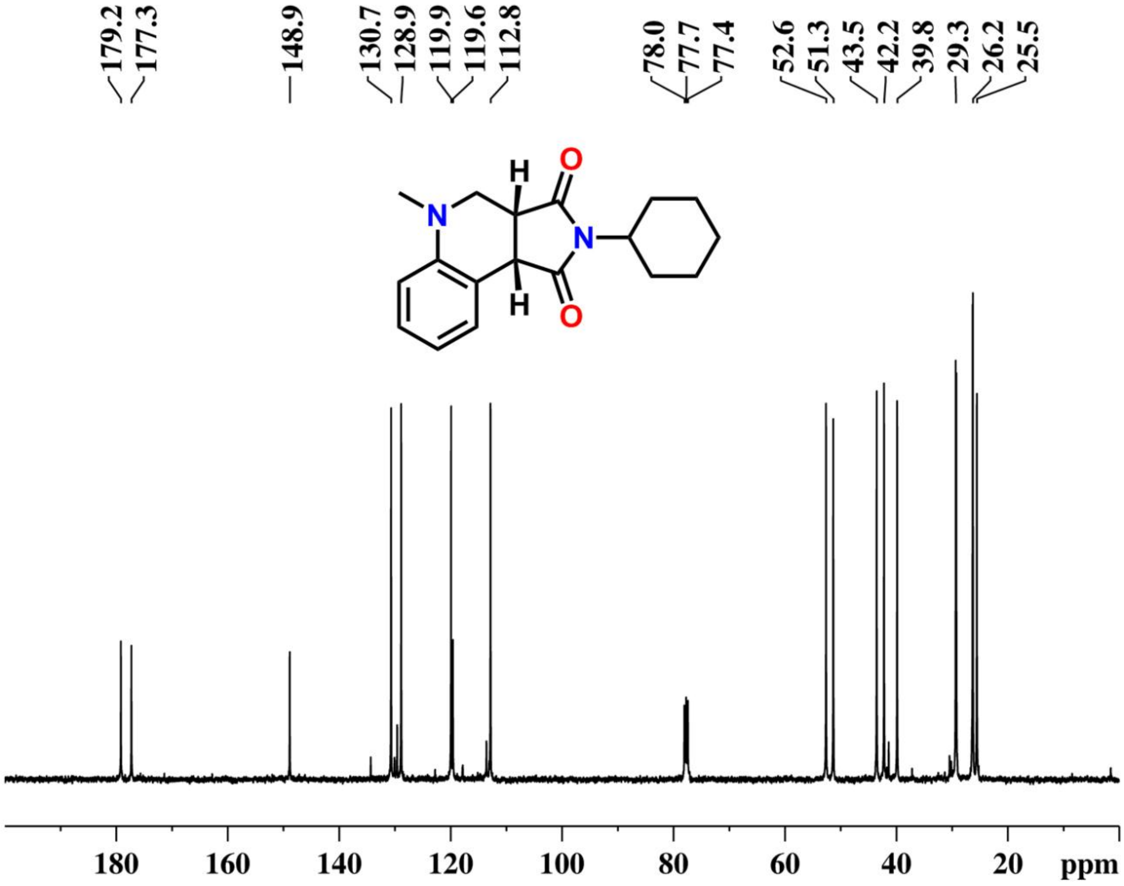

Fig. S79 ^{13}C NMR ($CDCl_3$, 100 MHz) of **6c**.

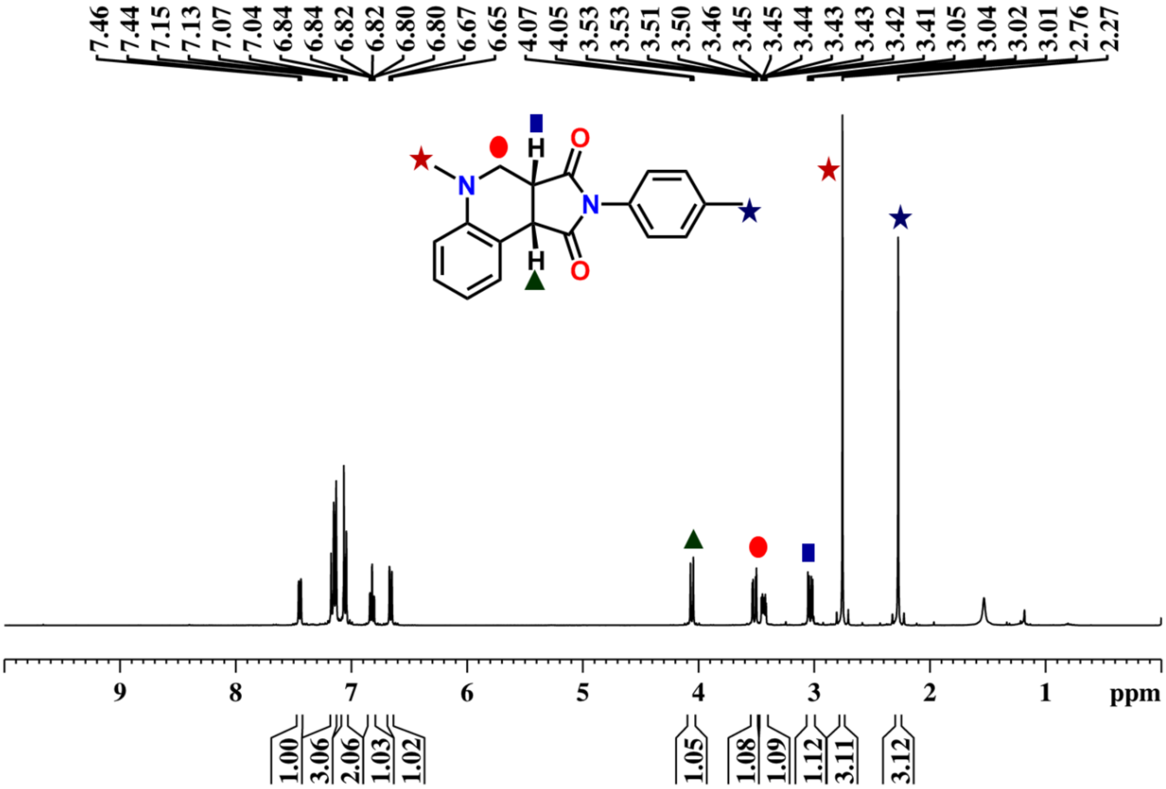

Fig. S80 ^{1}H NMR ($CDCl_3$, 400 MHz) of **6d**.

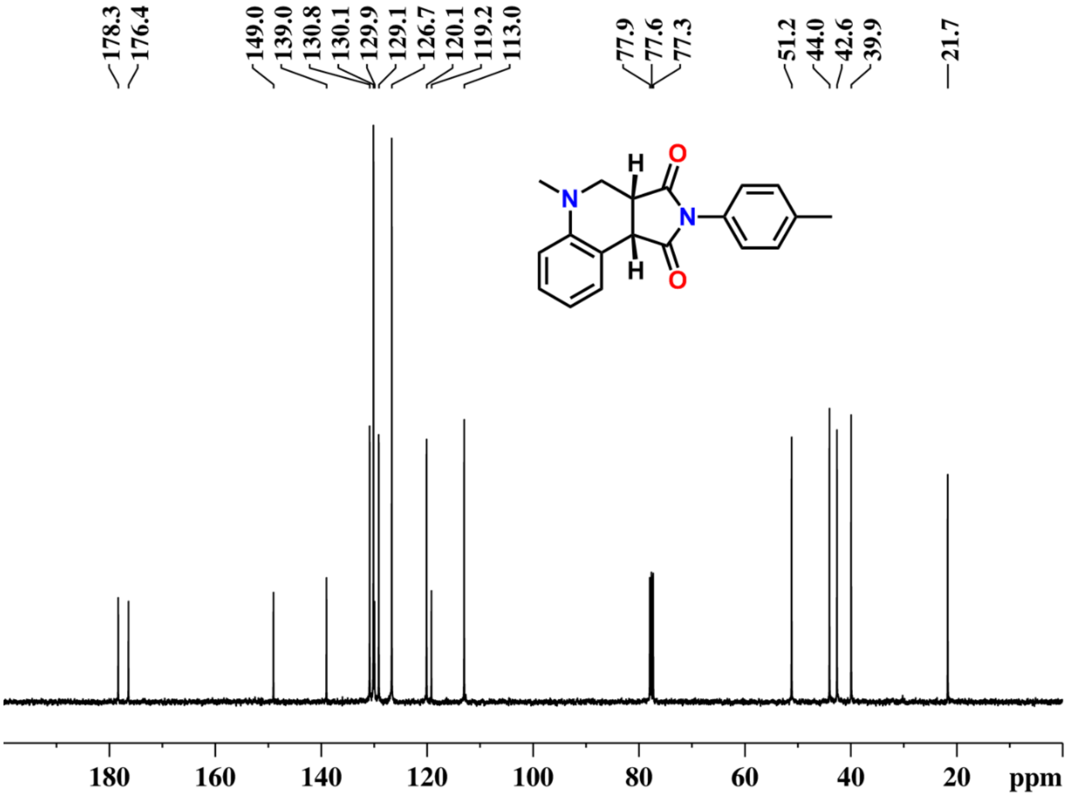

Fig. S81 ^{13}C NMR ($CDCl_3$, 100 MHz) of **6d**.

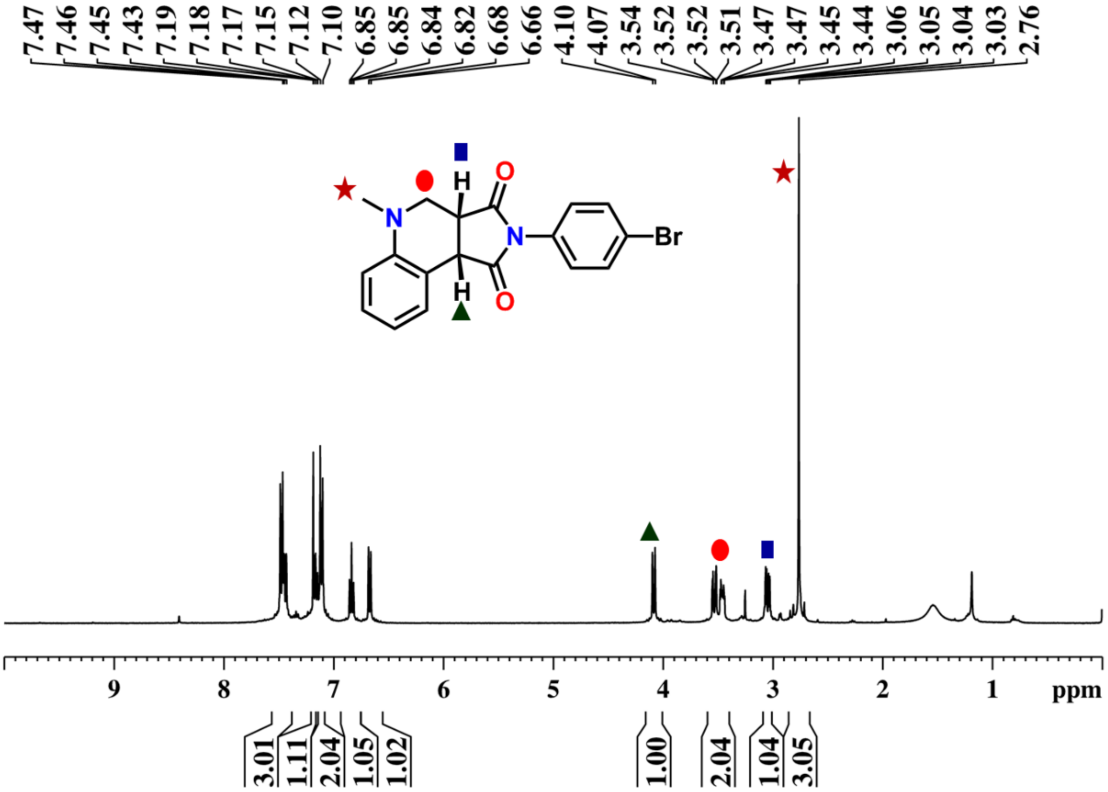

Fig. S82 ^{1}H NMR ($CDCl_3$, 400 MHz) of **6e**.

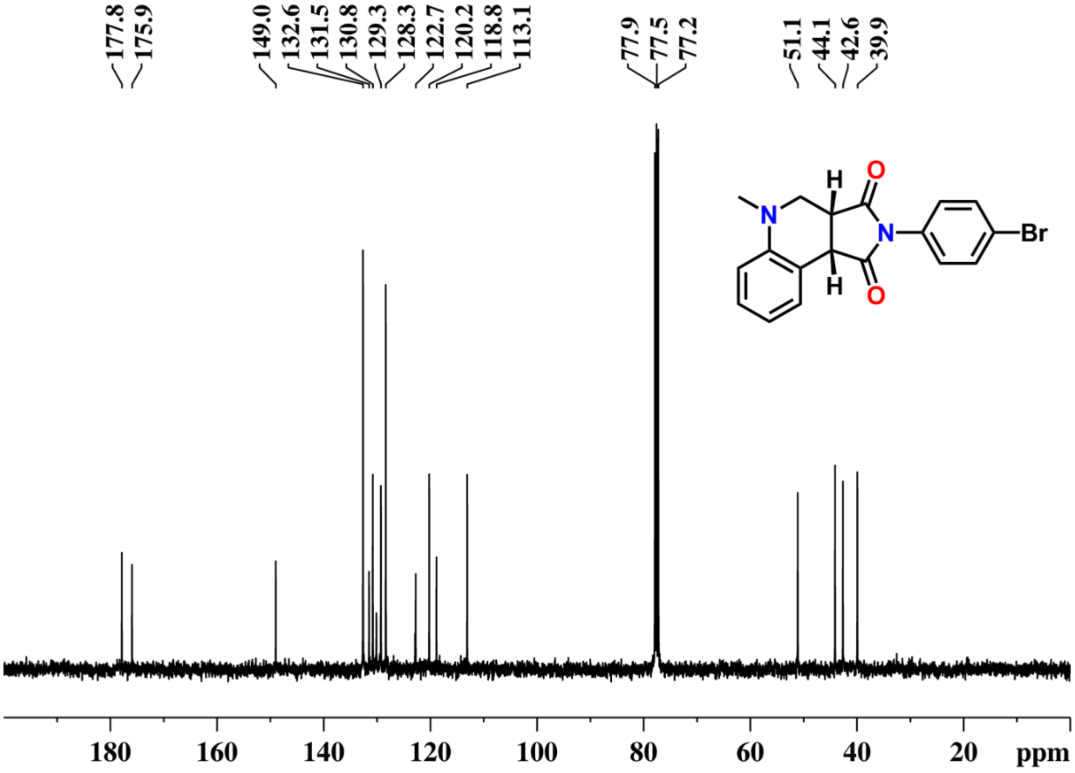

Fig. S83 ^{13}C NMR ($CDCl_3$, 100 MHz) of **6e**.

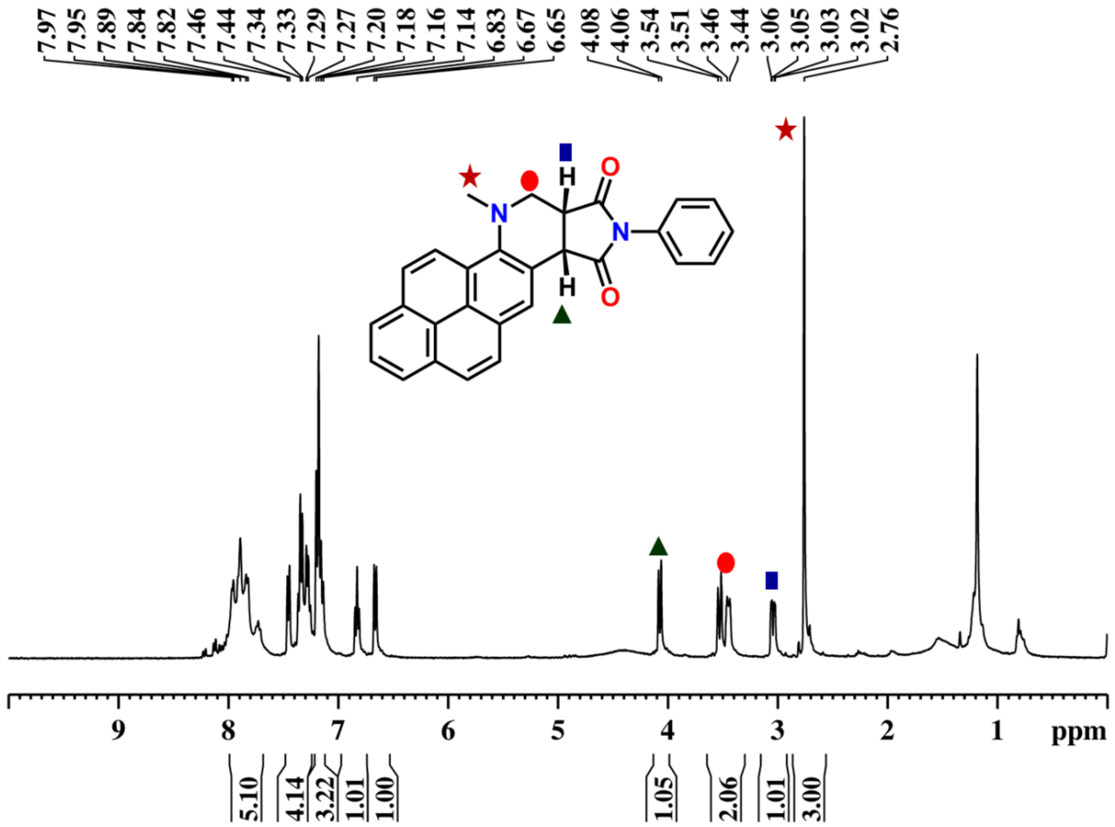

Fig. S84 ^{1}H NMR ($CDCl_3$, 400 MHz) of **6f**.

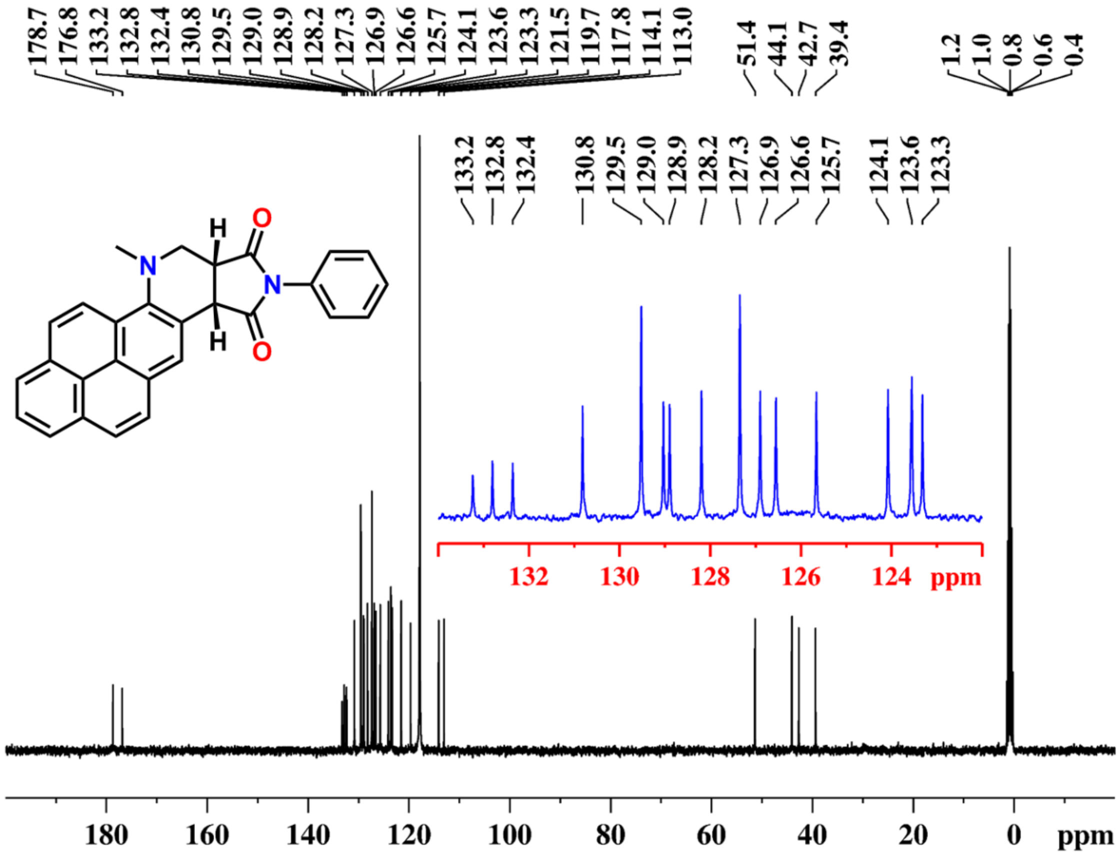

Fig. S85 ^{13}C NMR (CD_3CN, 100 MHz) of **6f**.

References

S1. (a) CIE, Commission internationale de l'Eclairage proceedings, 1931, *Cambridge University Press, Cambridge*, 1932; b) L. A. Jones, The Historical Background and Evolution of the Colorimetry Report *J. Opt. Soc. Am*., 1943, **33**, 534-543.

S2. (a) Y.-X. Zhu, Z.-W. Wei, M. Pan, H.-P. Wang, J.-Y. Zhang, H.-Y. Su, *Dalton transactions* 2016**,** **45,** 943-950; (b) S. Shanmugaraju and P. S. Mukherjee, *Chem. Eur. J.*, 2015, **21**, 6656-6666; (c) Z. Zhou, D.-G. Chen, M. L. Saha, H. Wang, X. Li, P.-T. Chou and P. J. Stang, *J. Am. Chem. Soc.*, 2019, **141**, 5535-5543.

S3. M. J. Frisch, G. W. Trucks, H. B. Schlegel, G. E. Scuseria, M. A. Robb, J. R. Cheeseman, G. Scalmani, V. Barone, B. Mennucci, G. A. Petersson, H. Nakatsuji, M. Caricato, X. Li, H. P. Hratchian, A. F. Izmaylov, J. Bloino, G. Zheng, J. L. Sonnenberg, M. Hada, M. Ehara, K. Toyota, R. Fukuda, J. Hasegawa, M. Ishida, T. Nakajima, Y. Honda, O. Kitao, H. Nakai, T. Vreven, J. A. Montgomery, J. E. Peralta, F. Ogliaro, M. Bearpark, J. J. Heyd, E. Brothers, K. N. Kudin, V. N. Staroverov, R. Kobayashi, J. Normand, K. Raghavachari, A. Rendell, J. C. Burant, S. S. Iyengar, J. Tomasi, M. Cossi, N. Rega, J. M. Millam, M. Klene, J. E. Knox, J. B. Cross, V. Bakken, C. Adamo, J. Jaramillo, R. Gomperts, R. E. Stratmann, O. Yazyev, A. J. Austin, R. Cammi, C. Pomelli, J. W. Ochterski, R. L. Martin, K. Morokuma, V. G. Zakrzewski, G. A. Voth, P. Salvador, J. J. Dannenberg, S. Dapprich, A. D. Daniels, Farkas, J. B. Foresman, J. V. Ortiz, J. Cioslowski and D. J. Fox, Wallingford CT, 2009.

S4. A. D. Becke, *Physical review A*, 1988, **38**, 3098.

S5. C. Lee, W. Yang and R. G. Parr, *Physical Review B,* 1988, **37**, 785-789.

S6. Z. Liang, S. Xu, W. Tian and R. Zhang, *Beilstein J. Org. Chem.*, 2015**, 11**, 425–430

Coordinate of L from Computational studies

C	0.03886000	0.68174300	-0.01291700
C	-0.03887500	-0.68177200	-0.01277400
C	1.33463500	1.41564900	0.11400800
C	2.25118000	1.11418300	1.13734200
C	1.64403500	2.47625600	-0.75246100
C	3.43708300	1.82864500	1.27344100
H	2.02424000	0.31404400	1.83473300
C	2.83695900	3.18528500	-0.62041000
H	0.94288400	2.74747300	-1.53644100
C	3.74979300	2.87032700	0.39072900
H	4.12030800	1.58726400	2.08368700
H	3.05585000	3.99695600	-1.31100000
C	-1.16464700	1.55853300	-0.14035700
C	-1.35533200	2.64268600	0.73387800
C	-2.10373900	1.37291600	-1.16766700
C	-2.45689000	3.48350300	0.60271100
H	-0.63072700	2.82749700	1.52184000
C	-3.20194500	2.22001500	-1.30409500
H	-1.96702600	0.55799500	-1.87159700
C	-3.39684800	3.28209400	-0.41538800

H	-2.58217100	4.31181400	1.29740200
H	-3.90419500	2.06018500	-2.11728700
C	1.16464000	-1.55858300	-0.13999400
C	2.10375900	-1.37317300	-1.16731600
C	1.35531100	-2.64255000	0.73447300
C	3.20197600	-2.22029200	-1.30353700
H	1.96706100	-0.55840100	-1.87142100
C	2.45687700	-3.48338900	0.60351100
H	0.63068700	-2.82720200	1.52245600
C	3.39686100	-3.28218500	-0.41460400
H	3.90424700	-2.06062300	-2.11674200
H	2.58214400	-4.31155300	1.29837900
C	-1.33464900	-1.41565600	0.11427300
C	-1.64401800	-2.47645900	-0.75196800
C	-2.25122800	-1.11396600	1.13751100
C	-2.83694000	-3.18546800	-0.61979200
H	-0.94284300	-2.74784700	-1.53586700
C	-3.43713000	-1.82840700	1.27373600
H	-2.02431700	-0.31366900	1.83472900
C	-3.74980600	-2.87029200	0.39125000
H	-3.05580300	-3.99729600	-1.31020600
H	-4.12038100	-1.58684800	2.08390700
C	4.60707800	-4.19950800	-0.50401000
H	4.33592100	-5.20500600	-0.16352100
H	5.39989100	-3.84997400	0.16819000
C	-5.05970600	-3.62858200	0.50666600
H	-5.89652900	-2.99955000	0.18050900
H	-5.04430000	-4.50217000	-0.15520200
C	-4.60705500	4.19940900	-0.50501600
H	-5.39989300	3.85001300	0.16722400
H	-4.33590000	5.20497100	-0.16471400
C	5.05969700	3.62863000	0.50601200
H	5.89652600	2.99950500	0.18004700
H	5.04433100	4.50205400	-0.15607200
N	5.37393900	4.06168900	1.86300500
C	6.43994400	3.66356500	2.62505800
C	4.65618000	4.95417200	2.63676100
H	7.17483900	2.96227900	2.24962600
C	5.33719800	5.04178000	3.82521800
H	3.75054100	5.41760000	2.27462100
H	5.09038500	5.64247400	4.69061000
N	-5.19140300	4.30752100	-1.83430500
C	-6.28179500	3.60925500	-2.32116500
C	-4.71921400	5.05696500	-2.87824000
C	-6.40762200	3.98818000	-3.63441900
H	-6.85080000	2.94002100	-1.69235300
H	-3.85554300	5.70080100	-2.77130300
H	-7.15165300	3.66831100	-4.35169900
N	-5.37400000	-4.06131300	1.86375100
C	-6.44004300	-3.66302100	2.62566200
C	-4.65626300	-4.95360200	2.63775200
H	-7.17493400	-2.96183800	2.25002800
C	-5.33732300	-5.04092100	3.82620700
H	-3.75060600	-5.41710900	2.27576000
H	-5.09053400	-5.64139700	4.69175600

N	5.19147300	-4.30787000	-1.83325800
C	6.28187900	-3.60969100	-2.32021300
C	4.71931400	-5.05750200	-2.87707200
C	6.40776100	-3.98887500	-3.63338700
H	6.85085700	-2.94033300	-1.69151000
H	3.85563600	-5.70131300	-2.77004600
H	7.15182200	-3.66914700	-4.35069900
N	-6.45156300	-4.23113500	3.81169300
N	-5.42792300	4.89428900	-3.97466200
N	5.42808600	-4.89506500	-3.97348700
N	6.45142900	4.23197600	3.81094700

Coordinate of 1 from Computational studies

N	-6.94663900	4.42964200	2.11077000
C	-6.23633200	3.42257300	2.62474600
C	-7.37873900	5.19910100	3.17512400
H	-5.76154500	2.64139400	2.05445500
C	-6.91537700	4.63095100	4.33406900
H	-7.99031000	6.07886500	3.04386000
H	-7.05488800	4.91783200	5.36621900
N	-7.02064000	4.50799100	-2.01600500
C	-6.23922600	3.58630500	-2.58580200
C	-7.62098200	5.21296900	-3.04310000
H	-5.62789100	2.87597000	-2.05541000
C	-7.18725100	4.69195800	-4.23470100
H	-8.30555100	6.02721500	-2.86349400
H	-7.43316600	4.95737000	-5.25267800
C	-5.74467200	2.70095600	-4.91267000
H	-6.40151200	1.82446200	-4.90648800
H	-5.85283500	3.17663700	-5.88918800
C	-4.19127900	2.08965600	4.71072700
C	-3.21622600	2.55496100	5.60362900
C	-3.79539300	1.16280900	3.73266100
C	-1.90156500	2.09498800	5.53455800
H	-3.48791300	3.25778800	6.38706200
C	-2.47659500	0.71003000	3.65514800
H	-4.53057700	0.75316800	3.04198800
C	-1.50488400	1.14425700	4.57442500
H	-1.18067000	2.45815700	6.26026800
H	-2.20654200	-0.01671000	2.89506500
C	-4.30064600	2.27560200	-4.70407200
C	-3.93807700	1.30256700	-3.75774200
C	-3.30156500	2.75607100	-5.56180100
C	-2.63031200	0.81883900	-3.68043400
H	-4.69248200	0.87962400	-3.09644800
C	-1.99903500	2.26184900	-5.49601400
H	-3.54637400	3.49604000	-6.31943400
C	-1.63664400	1.26283000	-4.57190400
H	-2.38795900	0.05656200	-2.94651200
H	-1.26144100	2.63576200	-6.19900100
N	4.65720800	6.74520200	2.09989900
C	4.06279500	5.75054000	2.76880200
C	5.10214300	7.65302500	3.04180500

H	3.61576800	4.87809800	2.32313700
C	4.75404000	7.19426400	4.28332700
H	5.62479100	8.55762900	2.77083900
H	4.92194000	7.61202700	5.26561600
N	4.61104200	6.74198700	-2.04447500
C	4.06974700	5.71285200	-2.70522300
C	5.01206100	7.66297300	-2.99349400
H	3.66635200	4.82250900	-2.25317400
C	4.68596700	7.17890800	-4.23108000
H	5.49818100	8.58956800	-2.72969800
H	4.83345000	7.59697900	-5.21640600
C	3.64565100	5.08681200	-5.16489200
H	3.17677700	5.76871100	-5.88349300
H	4.54998800	4.69791400	-5.64260100
C	3.58696200	5.17445900	5.23205100
H	4.46160200	4.84266300	5.79862200
H	3.03779100	5.87263500	5.87436100
C	2.70730100	3.97679100	4.91444200
C	2.87324000	2.82019900	5.69180100
C	1.63264900	4.00688700	4.01323900
C	1.99539200	1.74069600	5.58832700
H	3.67344900	2.77453000	6.42613500
C	0.77416300	2.91383700	3.88771700
H	1.43268300	4.89950200	3.42555200
C	0.92876000	1.75381200	4.67256000
H	2.13471200	0.88901400	6.24496000
H	-0.05566900	2.97629800	3.18979800
C	2.69719400	3.93503200	-4.86993600
C	2.80094800	2.79513400	-5.68301500
C	1.63368000	3.98954800	-3.95822700
C	1.87539700	1.75524100	-5.60157600
H	3.59245700	2.73314000	-6.42578900
C	0.72629900	2.93272300	-3.85536100
H	1.48218000	4.87130100	-3.34120900
C	0.82023300	1.78948900	-4.67257500
H	1.96952700	0.91684900	-6.28298500
H	-0.09205200	3.01219400	-3.14554300
N	-4.60860400	-6.78339600	2.08877100
C	-3.93307400	-5.82609600	2.73711500
C	-5.09395800	-7.65054800	3.05056400
H	-3.43123400	-4.99282700	2.27530400
C	-4.69258600	-7.20322900	4.27981000
H	-5.69004300	-8.51417900	2.80128900
H	-4.86994400	-7.59885100	5.26959500
N	-4.61371500	-6.80264400	-2.06125600
C	-4.19393800	-5.71306000	-2.71471800
C	-4.83106600	-7.78070000	-3.01427600
H	-3.94340400	-4.76851800	-2.26099400
C	-4.51949500	-7.26618900	-4.24336000
H	-5.19792800	-8.76379400	-2.76264700
H	-4.56809700	-7.70752000	-5.22838600
C	-3.79932400	-5.02843600	-5.15325400
H	-3.37539600	-5.65715900	-5.94400800
H	-4.75063400	-4.64313600	-5.53404100
C	-3.34932900	-5.28083100	5.19032700

H	-4.17241800	-4.99452400	5.85032600
H	-2.73476200	-6.00234000	5.74122800
C	-2.51595600	-4.05341100	4.86321600
C	-2.69492600	-2.90632700	5.65165700
C	-1.45041200	-4.05496400	3.94996300
C	-1.83346800	-1.81243100	5.55352300
H	-3.48809300	-2.88107000	6.39447000
C	-0.60902700	-2.94853700	3.83046200
H	-1.23852300	-4.93958100	3.35486100
C	-0.76996200	-1.80185600	4.63416600
H	-1.97911200	-0.97130800	6.22225400
H	0.21696700	-2.99177400	3.12654200
C	-2.85630400	-3.86809300	-4.86976500
C	-2.97569800	-2.73053300	-5.68440800
C	-1.79588900	-3.90797700	-3.95597700
C	-2.06373900	-1.67871900	-5.60286400
H	-3.76869300	-2.67893300	-6.42659300
C	-0.89993200	-2.84139400	-3.85627500
H	-1.63615100	-4.78509500	-3.33565400
C	-1.00652100	-1.70161700	-4.67535100
H	-2.16878000	-0.84036100	-6.28298300
H	-0.08024600	-2.91047300	-3.14683100
N	7.01571600	-4.41998600	1.99545000
C	6.25148300	-3.47302200	2.54617500
C	7.65413600	-5.07337000	3.03284000
H	5.61849900	-2.78926800	2.00563000
C	7.26024200	-4.49723600	4.21271600
H	8.33851800	-5.89130800	2.86697500
H	7.54165100	-4.71333100	5.23311500
N	6.80931700	-4.55652700	-2.12623800
C	6.11375600	-3.55509700	-2.67204300
C	7.21944100	-5.37068400	-3.16587400
H	5.65942800	-2.74345800	-2.12873300
C	6.75682900	-4.83661400	-4.34113800
H	7.81662100	-6.25531400	-3.00630700
H	6.88087500	-5.16416600	-5.36307400
C	5.52520500	-2.68857800	-4.97831700
H	6.20648400	-1.83184300	-4.94903900
H	5.62871000	-3.14571300	-5.96396900
C	5.81850000	-2.48752900	4.84322900
H	5.96737400	-2.91245400	5.83767100
H	6.45521800	-1.59921100	4.77293100
C	4.35938800	-2.10451200	4.65763700
C	3.38715100	-2.60179600	5.53660300
C	3.95483700	-1.15893300	3.70084600
C	2.06811000	-2.15270000	5.47709600
H	3.66502000	-3.32024600	6.30353300
C	2.63112800	-0.71870600	3.63165100
H	4.68783500	-0.72507900	3.02270000
C	1.66288500	-1.18247500	4.54014800
H	1.35065100	-2.53918300	6.19415800
H	2.35504600	0.02372100	2.88908700
C	4.09113300	-2.23083100	-4.76778200
C	3.74903300	-1.26669200	-3.80526600
C	3.08235500	-2.67846000	-5.63147000

C	2.44998400	-0.76256200	-3.71421300
H	4.51219200	-0.86754400	-3.13925900
C	1.78822800	-2.16558700	-5.55060900
H	3.31210600	-3.40956400	-6.40231200
C	1.44520100	-1.17891600	-4.60616700
H	2.22270200	-0.00845400	-2.96707100
H	1.04096900	-2.51696400	-6.25494800
C	-0.23329600	0.71746400	-4.59035300
C	0.04444900	-0.62652000	-4.59936700
C	0.24017300	-0.68832500	4.55890300
C	-0.08451900	0.64495900	4.57682100
Pt	7.05812900	-4.64491600	-0.06437500
Pt	-4.70455900	-6.91286300	0.01484300
Pt	4.69273000	6.86200800	0.02711700
Pt	-7.13613400	4.61943300	0.05303700
N	-4.11112400	-5.96072300	-4.04086100
N	6.05535200	-3.69211600	-4.01277800
N	-6.30946900	3.66839500	-3.93004400
N	4.08476600	5.94884700	-4.03644900
N	-3.95864100	-6.05056100	4.06828500
N	6.36855800	-3.49163100	3.88997800
N	4.09401100	5.99350100	4.09740200
N	-6.19059700	3.51172600	3.97021700
C	-5.64653400	2.48512500	4.90302700
H	-6.29340200	1.60596400	4.81694000
H	-5.79211700	2.89236500	5.90511800
P	-9.18574400	3.29132000	0.07966000
P	-5.56236700	6.46482000	0.08752900
P	2.77478200	8.40500500	0.07213700
P	6.93489800	5.94577300	0.04537100
P	9.15573700	-3.41404100	-0.22792600
P	5.33434300	-6.34151500	0.03902500
P	-2.94739800	-8.61501300	0.06391700
P	-6.94554100	-5.98887400	-0.01913800
C	2.06318200	8.59105200	1.78773700
H	2.03567900	7.58736700	2.22698500
H	2.81285800	9.15067400	2.35765900
C	3.48843700	10.05653700	-0.40004000
H	3.86191000	9.94045000	-1.42406900
H	4.37563500	10.19097900	0.23095800
C	1.28687800	8.13838500	-1.01618800
H	0.91722700	7.12636100	-0.80522200
H	0.52170400	8.83263200	-0.65366400
C	0.68509500	9.25611500	1.93849200
H	-0.11385900	8.63924500	1.51415000
H	0.46852000	9.38894300	3.00365600
H	0.63754600	10.24338100	1.47441300
C	2.57830000	11.29248000	-0.30435300
H	2.31054600	11.52517200	0.72947800
H	3.11452300	12.16027600	-0.70098400
H	1.65897000	11.18285000	-0.88820700
C	1.47635000	8.34876400	-2.52386800
H	2.19693400	7.65190100	-2.95580900
H	0.51901900	8.20203700	-3.03515900
H	1.80686200	9.36542900	-2.75446900

C	7.21642000	4.45776100	1.13852900
H	7.99951700	3.86236000	0.65577100
H	6.29682600	3.86085800	1.10195200
C	8.10986000	7.25597700	0.62623300
H	9.10799200	6.80417400	0.66970200
H	7.83607700	7.50362900	1.65729200
C	7.54757300	5.41665900	-1.62591000
H	6.93994700	4.54945500	-1.91636500
H	7.29520200	6.21792300	-2.32723500
C	9.04763800	5.09237600	-1.72115200
H	9.35519300	4.29876600	-1.03241800
H	9.28115800	4.75431400	-2.73577600
H	9.66932800	5.97021000	-1.52500200
C	7.62146600	4.74564100	2.59255600
H	6.89091800	5.36741400	3.11739000
H	7.71468200	3.80215200	3.14049900
H	8.59102300	5.24730200	2.65015900
C	8.12846000	8.52139800	-0.24438200
H	8.43087200	8.31039000	-1.27501200
H	7.14848200	9.01216400	-0.26424800
H	8.84936600	9.23616400	0.16342600
C	10.50501300	-4.60929100	-0.66205200
H	11.42357800	-4.02800500	-0.80683200
H	10.25344100	-5.04278200	-1.63628100
C	9.22581300	-2.04959600	-1.49874700
H	8.23698300	-1.57411000	-1.50632300
H	9.92895000	-1.30423600	-1.11080200
C	9.68166500	-2.58718100	1.35066300
H	8.96535600	-1.77207800	1.52132700
H	9.53025000	-3.30645300	2.16098900
C	9.64953400	-2.45336500	-2.91935400
H	9.00471600	-3.22436600	-3.35057500
H	9.61402700	-1.57650300	-3.57416000
H	10.67716400	-2.82522000	-2.94306300
C	10.72962900	-5.71799400	0.37699100
H	11.03245300	-5.31826900	1.34995000
H	9.83090700	-6.32980200	0.51626400
H	11.53164200	-6.38006400	0.03732100
C	11.12635200	-2.05992200	1.38202200
H	11.33392900	-1.33789600	0.58610100
H	11.30593900	-1.55374500	2.33581200
H	11.85806300	-2.86867600	1.30498800
C	4.90012900	-6.89153200	1.75728600
H	4.53237600	-6.01050300	2.29632300
H	5.84480000	-7.16538100	2.23866600
C	5.87637100	-7.86864900	-0.86047900
H	5.05497700	-8.59251200	-0.79971600
H	5.96842100	-7.59607600	-1.91798500
C	7.18221600	-8.48927600	-0.34235200
H	8.02111300	-7.78883500	-0.41964700
H	7.10134000	-8.81073800	0.70096000
H	7.43135100	-9.37540200	-0.93372700
C	3.90009600	-8.05239400	1.86531700
H	3.73834000	-8.29496000	2.92049900
H	2.92667300	-7.80098100	1.42979100

H	4.26820400	-8.96109800	1.38056500
C	3.73392700	-5.89676800	-0.78725200
H	3.08798900	-6.78260300	-0.76967900
H	3.97170000	-5.70513000	-1.83984400
C	3.01763600	-4.68861100	-0.17238500
H	3.64842600	-3.79299500	-0.20571600
H	2.09855200	-4.46872700	-0.72682300
H	2.74105000	-4.86650300	0.87253000
C	-1.78535300	-8.73898500	-1.37909400
H	-1.09847300	-9.56701200	-1.16864300
H	-2.39525300	-9.05587100	-2.23216000
C	-3.90672100	-10.21202800	0.02807000
H	-4.50173700	-10.22928500	0.94968900
H	-4.62687900	-10.12880800	-0.79444600
C	-1.92157300	-8.76784100	1.60383100
H	-1.47262000	-9.76735700	1.58368300
H	-2.62790300	-8.76573400	2.44058800
C	-1.00755200	-7.46773800	-1.74222000
H	-0.29266900	-7.18450200	-0.96460400
H	-0.44554900	-7.63312000	-2.66744800
H	-1.68287400	-6.62109200	-1.90098000
C	-0.83091700	-7.71169500	1.82078600
H	-0.40764200	-7.82263200	2.82467500
H	-0.01020500	-7.82905300	1.10721100
H	-1.22037400	-6.69145000	1.72513200
C	-3.09464500	-11.51144100	-0.10950100
H	-2.54256800	-11.55642100	-1.05274100
H	-2.39014600	-11.65500000	0.71507600
H	-3.78275600	-12.36233900	-0.09511500
C	-8.13377800	-7.39720400	-0.25002200
H	-9.13870100	-6.95631300	-0.28494400
H	-7.94199100	-7.82112500	-1.24170000
C	-7.49625900	-5.16835400	1.54857800
H	-8.58921800	-5.08729000	1.49149600
H	-7.27610900	-5.84892400	2.37641300
C	-7.40533100	-4.75552800	-1.34263500
H	-8.24319400	-4.17790300	-0.93207000
H	-6.56424300	-4.05979600	-1.44783600
C	-7.81084400	-5.33226200	-2.70825500
H	-8.01966800	-4.51133200	-3.40198100
H	-7.03162700	-5.95782900	-3.15193800
H	-8.72288200	-5.93061400	-2.64008000
C	-8.07245800	-8.49887100	0.81685700
H	-7.09660400	-8.99765700	0.82170200
H	-8.28135100	-8.11830800	1.82152900
H	-8.82840200	-9.25877500	0.59740900
C	-6.87205600	-3.79082600	1.80563500
H	-5.77831500	-3.84358200	1.84550200
H	-7.14932400	-3.06877700	1.03023300
H	-7.22639000	-3.39777000	2.76402300
C	-9.66616300	2.49920400	-1.52899000
H	-10.73992200	2.28514900	-1.45249600
H	-9.55466000	3.24999900	-2.31672300
C	-9.33901900	1.89766100	1.31022400
H	-8.39112500	1.34596800	1.30034100

H	-10.09918000	1.22227700	0.89794600
C	-10.61012800	4.42011100	0.46204900
H	-11.48702700	3.77460300	0.60249800
H	-10.40656600	4.88925000	1.43081400
C	-8.90463800	1.21797900	-1.89357200
H	-7.82454000	1.38994200	-1.96852100
H	-9.07185500	0.42216800	-1.16095900
H	-9.25615900	0.84711200	-2.86174800
C	-9.73400700	2.28158000	2.74439600
H	-9.77058500	1.37964200	3.36406200
H	-9.03158300	2.98084100	3.20524200
H	-10.72913500	2.73183500	2.78336400
C	-10.90748800	5.48868200	-0.59963200
H	-10.06222600	6.17130500	-0.73813400
H	-11.16687100	5.05060300	-1.56818500
H	-11.76432900	6.08855900	-0.27868700
C	-4.56299100	6.74041400	-1.45080400
H	-4.17416300	7.76470300	-1.40525700
H	-5.25821100	6.71214400	-2.29547000
C	-4.33913000	6.42577900	1.48627200
H	-3.77150600	5.49244700	1.38652400
H	-4.92690800	6.33303600	2.40595800
C	-6.50704500	8.04581400	0.32396700
H	-5.77208200	8.85695700	0.38754800
H	-6.99121900	7.98373100	1.30582600
C	-3.41905800	5.74082300	-1.66469400
H	-2.91699700	5.94944700	-2.61515000
H	-2.66873300	5.80724700	-0.86937400
H	-3.78028800	4.70777700	-1.70199800
C	-3.39200200	7.63135400	1.58878600
H	-2.71094900	7.49006900	2.43499500
H	-2.78445600	7.75730100	0.68615900
H	-3.93208500	8.56534300	1.76560200
C	-7.54257800	8.35930900	-0.76488000
H	-7.08321400	8.47169400	-1.75213300
H	-8.31209000	7.58285800	-0.82115100
H	-8.04347300	9.30369000	-0.53232700

Coordinate of 2 from Computational studies

N	7.83955500	7.56676000	4.22611400
C	7.85048500	6.39233600	4.84980200
C	9.16080200	7.92412600	4.04450700
H	6.97518700	5.84144000	5.15835600
C	9.96332100	6.94343800	4.56736600
H	9.43931400	8.85551200	3.57565100
H	11.03748700	6.86397900	4.63953300
N	-7.21908400	9.08184300	-0.80689200
C	-7.71984800	7.86824900	-0.59346000
C	-8.21693500	9.81662100	-1.41705200
H	-7.19574500	7.05092800	-0.12238000
C	-9.32507800	9.02395800	-1.56223500
H	-8.07544600	10.85023500	-1.69431800
H	-10.30281700	9.23295300	-1.96975100

C	-9.92931300	6.65669100	-0.83151100
H	-9.96321300	6.45675400	0.24386100
H	-10.91501500	7.03308900	-1.11619000
C	9.59305000	3.47499600	4.98393200
C	10.82865700	2.98495600	4.54169300
C	8.44767100	2.70753200	4.72891600
C	10.91857000	1.75803100	3.88478900
H	11.73754900	3.54978200	4.73436700
C	8.53642800	1.47647400	4.07950800
H	7.47375800	3.05840100	5.06500000
C	9.77854800	0.96307300	3.66785000
H	11.89206500	1.39782400	3.56595600
H	7.63654200	0.89219800	3.91389700
C	-9.62917000	5.37180900	-1.58387000
C	-9.09814900	4.26110000	-0.91388000
C	-9.99157600	5.22038500	-2.93014200
C	-8.97926200	3.02392800	-1.54909400
H	-8.80697700	4.35004900	0.13091100
C	-9.86550500	3.98836500	-3.56855800
H	-10.41179200	6.06033500	-3.47813000
C	-9.39809200	2.85184700	-2.87898100
H	-8.59191600	2.17348900	-0.99665800
H	-10.17974200	3.89352900	-4.60349600
N	11.42193500	0.87045200	-4.84726500
C	11.46698900	0.32907800	-3.63082800
C	12.67350700	1.40131800	-5.08814100
H	10.65098600	-0.17461400	-3.13766900
C	13.46665500	1.18218600	-3.99454800
H	12.91213100	1.89646200	-6.01699800
H	14.49913300	1.43153800	-3.80020900
N	-3.78670800	2.67955900	-9.62667100
C	-4.66043000	1.75574100	-9.23892100
C	-4.52489700	3.80822700	-9.93159200
H	-4.41646700	0.74535800	-8.94759600
C	-5.85164500	3.54806200	-9.71438300
H	-4.05867100	4.70936400	-10.29874000
H	-6.73211300	4.15727600	-9.85259700
C	-7.15127700	1.48239500	-8.98665100
H	-7.90741300	1.80885000	-9.70694900
H	-6.93256800	0.43576700	-9.22698000
C	13.22125000	-0.05705300	-1.81389500
H	13.64164200	-1.04361900	-2.03647300
H	14.06272500	0.58750700	-1.53617300
C	12.25808800	-0.16737700	-0.64392900
C	12.47114000	-1.19616700	0.28545600
C	11.25365400	0.76915500	-0.36989800
C	11.71876900	-1.28172200	1.45495400
H	13.26017200	-1.92409900	0.11233600
C	10.48607300	0.67033600	0.79141000
H	11.07797100	1.59744900	-1.05009300
C	10.69673900	-0.35590000	1.73148200
H	11.93926000	-2.07013300	2.16630000
H	9.72133100	1.41769700	0.98198200
C	-7.70086800	1.58241700	-7.56841600
C	-8.99875400	1.10223900	-7.34035900

C	-6.97383700	2.07147200	-6.47952200
C	-9.53727000	1.07379500	-6.05687400
H	-9.59922500	0.74493400	-8.17395400
C	-7.52367700	2.06330300	-5.19445300
H	-5.97471800	2.47278900	-6.62718700
C	-8.80255400	1.53616300	-4.95026600
H	-10.54330700	0.69402800	-5.91135600
H	-6.94379000	2.46124400	-4.36588600
N	3.77991000	-2.60665100	9.62940200
C	4.69022700	-1.70645000	9.27268000
C	4.46751000	-3.78112000	9.87180000
H	4.48960700	-0.67396200	9.02886100
C	5.80330200	-3.57038600	9.65388500
H	3.96174500	-4.67768600	10.19614800
H	6.65701600	-4.22331000	9.75573000
N	-11.45024300	-0.94793500	4.82037800
C	-11.49219900	-0.38920700	3.61177700
C	-12.70176700	-1.48394500	5.04966400
H	-10.67544600	0.12278700	3.12850500
C	-13.49183800	-1.25001700	3.95678800
H	-12.94249900	-1.99264700	5.97063100
H	-14.52332000	-1.49799900	3.75554800
C	-13.23812900	0.02001100	1.79353100
H	-14.08027600	-0.61865900	1.50455800
H	-13.65630400	1.00529800	2.02571300
C	7.19030300	-1.53480300	9.01047600
H	7.02227900	-0.49071900	9.29683500
H	7.93327700	-1.92676200	9.71197100
C	7.72847500	-1.60051200	7.58558100
C	9.00975100	-1.07936600	7.35177700
C	7.01414700	-2.11515300	6.50066300
C	9.54534200	-1.04242600	6.06748500
H	9.60107900	-0.70004900	8.18225300
C	7.56019600	-2.09372200	5.21385800
H	6.03074600	-2.55065200	6.65351500
C	8.82332400	-1.53281100	4.96406400
H	10.54006400	-0.63517600	5.91913900
H	6.99044400	-2.51241500	4.38845300
C	-12.26806600	0.14010700	0.63029300
C	-12.46759800	1.18255100	-0.28671300
C	-11.26862700	-0.80009800	0.35054000
C	-11.70739400	1.27739900	-1.45052900
H	-13.25215700	1.91418300	-0.10890700
C	-10.49334500	-0.69224700	-0.80462100
H	-11.10318500	-1.63814400	1.02128600
C	-10.69114400	0.34705100	-1.73312100
H	-11.91776000	2.07634000	-2.15315800
H	-9.73304600	-1.44281100	-1.00012800
N	7.28733300	-9.07309500	0.78579300
C	7.78453400	-7.84665800	0.65356100
C	8.29870800	-9.85245300	1.31280500
H	7.24853200	-6.99464700	0.26480100
C	9.41227400	-9.07380400	1.48754400
H	8.15912900	-10.90202900	1.52216500
H	10.39926400	-9.31328700	1.85396800

N	-7.80044700	-7.54123500	-4.25589700
C	-7.82336000	-6.36749900	-4.88064300
C	-9.11808100	-7.90959700	-4.06985100
H	-6.95380500	-5.80924300	-5.19216900
C	-9.93050800	-6.93605900	-4.59080500
H	-9.38724300	-8.84268700	-3.59895200
H	-11.00553900	-6.86560900	-4.65942900
C	-9.50552700	-4.73932100	-5.82845200
H	-8.79345900	-4.61328100	-6.64954800
H	-10.47601300	-4.95885000	-6.27948000
C	10.01571400	-6.67228000	0.89305900
H	10.98882300	-7.05063200	1.21536700
H	10.08954400	-6.47502600	-0.18106900
C	9.69318600	-5.38562900	1.63170400
C	10.05460000	-5.21709200	2.97618400
C	9.14554300	-4.28852400	0.95232000
C	9.91358700	-3.98100700	3.60330500
H	10.48722800	-6.04645700	3.53059600
C	9.00994900	-3.04750500	1.57691900
H	8.85627200	-4.39044300	-0.09185400
C	9.42980200	-2.85737700	2.90405300
H	10.22933700	-3.87215500	4.63638400
H	8.61084000	-2.20731500	1.01717800
C	-9.58445900	-3.46501800	-5.00425300
C	-8.43841400	-2.70204300	-4.73896700
C	-10.82132900	-2.97304600	-4.56793000
C	-8.52747500	-1.47423400	-4.08371400
H	-7.46336100	-3.05403000	-5.07057500
C	-10.91163700	-1.74887000	-3.90576700
H	-11.73064600	-3.53434700	-4.76870100
C	-9.77051700	-0.95912800	-3.67674400
H	-7.62672000	-0.89407300	-3.90890200
H	-11.88602100	-1.38740500	-3.59106300
C	-9.38093000	1.51883800	-3.56447700
C	-9.90196200	0.38034800	-3.00818000
C	9.40035900	-1.51818900	3.57779900
C	9.91263900	-0.38130300	3.01007200
N	-12.71334100	-0.56042400	3.04622400
N	-9.09672700	-5.96153600	-5.10199900
N	-8.99706400	7.78802000	-1.03870900
N	-5.92698700	2.24097600	-9.27329200
N	5.93397300	-2.24918500	9.27177100
N	9.07328000	-7.80083200	1.07015300
N	12.69021700	0.50687700	-3.07155500
N	9.11969000	5.97550400	5.07504900
C	9.51562600	4.75130700	5.80523200
H	8.79705200	4.63044600	6.62136600
H	10.48421300	4.96546100	6.26297000
P	9.29549800	3.30624900	-5.69821900
P	10.07050200	-1.18307100	-7.08088000
P	5.91885400	-10.44152100	-1.95570100
P	4.45388500	-9.29753000	2.42645100
P	6.25974400	7.90146700	1.31644300
P	5.72170900	9.68472500	5.70242100
P	1.40858900	-2.94550700	7.36284000

P	1.52618700	-1.80305600	11.99768600
Pt	9.68306400	0.97848100	-6.12139600
Pt	5.28800300	-9.62204600	0.20440000
Pt	6.02885000	8.56079200	3.60611700
Pt	1.63951300	-2.31471600	9.66052900
C	7.99414500	1.07439100	-7.13161200
C	6.88527800	1.17217400	-7.64638300
C	3.43644300	-9.91113400	-0.39580800
C	2.28074200	-9.94729800	-0.80391200
C	4.27443300	9.24884400	3.03569800
C	3.14149500	9.51964400	2.65333700
C	-0.30732700	-2.03139300	9.54424400
C	-1.50237100	-1.86873900	9.32217200
C	5.55835700	1.30215900	-8.16641500
C	5.19814000	2.36596300	-9.02156800
C	4.54649900	0.39232100	-7.79016000
C	3.88426700	2.53802500	-9.44290600
H	5.96115300	3.06637400	-9.34746700
C	3.23405600	0.56380100	-8.21029000
H	4.80305100	-0.44602800	-7.14920200
C	2.86572500	1.65415800	-9.02813300
H	3.63212900	3.37236700	-10.09039400
H	2.47108300	-0.14486400	-7.90050900
C	0.93698700	-9.87367800	-1.29053100
C	0.45877400	-8.65735600	-1.82548500
C	0.04928000	-10.96896300	-1.25008100
C	-0.84496600	-8.53931600	-2.28353400
H	1.13017700	-7.80470300	-1.87423900
C	-1.25899600	-10.85068400	-1.71035900
H	0.39410400	-11.91834800	-0.85189900
C	-1.73957300	-9.63117600	-2.23079900
H	-1.19226700	-7.59470700	-2.69255800
H	-1.92314800	-11.70844700	-1.66586400
C	1.79354300	9.70033400	2.20879800
C	0.89431400	8.61279800	2.27154700
C	1.31586500	10.91936700	1.68463600
C	-0.41134000	8.73473600	1.82046700
H	1.23959500	7.66862700	2.68326400
C	0.00559200	11.04144200	1.23090600
H	1.98354500	11.77395200	1.63257900
C	-0.88714200	9.95070400	1.28236700
H	-1.08638700	7.88556300	1.87747500
H	-0.33731000	11.99068400	0.83074800
C	-2.88038300	-1.67991700	8.98394500
C	-3.86170700	-2.63948900	9.31147600
C	-3.29137900	-0.54212100	8.25602800
C	-5.17906300	-2.48996900	8.89270800
H	-3.57737100	-3.51275200	9.89082000
C	-4.60741500	-0.39294600	7.83893900
H	-2.55940700	0.22370500	8.01544000
C	-5.58081200	-1.37396300	8.12743400
H	-5.91277500	-3.24713300	9.15238600
H	-4.89657300	0.48515700	7.26906800
C	-3.08664800	-9.45541100	-2.68003800
C	-4.22056300	-9.19124900	-3.06415100

C	1.49129800	1.87907700	-9.36102600
C	0.29901000	2.08752600	-9.55744000
C	-2.23432600	10.02747700	0.80647700
C	-3.39684500	9.99195500	0.41872100
C	-6.91024100	-1.25645500	7.61123200
C	-8.02174000	-1.16321600	7.10130400
Pt	-1.64362400	2.41686800	-9.64846800
Pt	-5.98030500	-8.51748200	-3.63589700
Pt	-9.71256400	-1.06549800	6.09479900
Pt	-5.24273700	9.67981200	-0.19136900
P	-6.21433400	-7.85013400	-1.34865100
P	-5.66777200	-9.64811700	-5.72729800
P	-1.52613100	1.59838200	-11.88700600
P	-1.34331100	3.24450500	-7.41941500
P	-4.31120000	9.57205900	-2.38651600
P	-5.99374100	10.32720300	1.98635600
P	-10.11184400	1.08341600	7.07771800
P	-9.31834800	-3.38787700	5.65084900
C	-9.62393900	1.10582700	8.86379500
H	-8.55524300	0.87778300	8.90463200
H	-9.76475000	2.13420600	9.22171700
C	-11.89154200	1.63704700	7.12879000
H	-12.49440700	0.75115200	7.35418800
H	-11.97978000	2.30191900	7.99728300
C	-9.20572400	2.52044300	6.32716300
H	-9.53246600	3.42381300	6.85886500
H	-9.55859800	2.61500000	5.29357200
C	-7.67641000	2.40301000	6.35616200
H	-7.33668700	1.49482400	5.84928800
H	-7.22761800	3.26999600	5.85822500
H	-7.29376400	2.37609100	7.38087100
C	-10.40445300	0.11522500	9.73916600
H	-11.47913200	0.32379700	9.74590800
H	-10.25165600	-0.91419000	9.39963300
H	-10.05364700	0.18220800	10.77363400
C	-12.43764600	2.34793400	5.88391900
H	-12.36684600	1.72410200	4.98796600
H	-13.49369000	2.59137800	6.03871900
H	-11.91278500	3.28840300	5.68867800
C	-9.45728200	-4.32479000	7.24341900
H	-9.20224000	-5.37354900	7.05032100
H	-8.68609900	-3.90906100	7.90069200
C	-10.47336600	-4.28198700	4.50201100
H	-10.34185200	-5.35801600	4.66981900
H	-11.49359200	-4.04116000	4.81582400
C	-7.61769700	-3.74841400	4.99991500
H	-7.53996700	-3.22802100	4.03748700
H	-6.93325400	-3.23356600	5.68083100
C	-10.83648500	-4.22890900	7.91266300
H	-10.82930800	-4.78136000	8.85732400
H	-11.09958300	-3.18952600	8.13638100
H	-11.62770900	-4.66163700	7.29104800
C	-10.28555500	-3.94443700	3.01797900
H	-11.06287600	-4.43157700	2.41960100
H	-10.34980300	-2.86522100	2.84571100

H	-9.31481200	-4.28658200	2.64336900
C	-7.23947600	-5.22832400	4.85045500
H	-7.91742700	-5.76776300	4.17988800
H	-6.22850500	-5.31249700	4.43523900
H	-7.23871600	-5.74836000	5.81293200
C	-7.71100400	9.75098400	2.41206500
H	-7.71469100	8.66037100	2.29623900
H	-8.36856100	10.14559800	1.62866000
C	-4.91934200	9.77781000	3.38719000
H	-3.92250800	10.17676800	3.17979000
H	-5.28309100	10.25539200	4.30474100
C	-6.08763300	12.17575000	2.11952300
H	-6.78708100	12.50590500	1.34181200
H	-6.55515300	12.40799100	3.08366000
C	-4.75335400	12.92296000	1.98918800
H	-4.06229200	12.66416600	2.79732700
H	-4.25662300	12.70682800	1.03952400
H	-4.93635100	14.00062200	2.04524400
C	-4.85038000	8.25417200	3.54706500
H	-5.82937900	7.82125400	3.78255300
H	-4.47467200	7.78425200	2.63144400
H	-4.17079700	7.99381600	4.36532400
C	-8.24259400	10.14004000	3.80018400
H	-8.29000600	11.22420700	3.93536300
H	-9.26053400	9.75602600	3.92675900
H	-7.63134200	9.72401000	4.60712500
C	-3.64763700	11.24568200	-2.82424400
H	-2.85653500	11.45050900	-2.09460500
H	-3.18059600	11.18616000	-3.81451900
C	-5.44030400	9.14955900	-3.80206800
H	-4.94118900	9.46265800	-4.72754000
H	-6.32890800	9.78088200	-3.69974300
C	-2.87203300	8.41007900	-2.53581700
H	-3.26277800	7.40538100	-2.33232500
H	-2.21627300	8.65907500	-1.69532400
C	-5.85107300	7.67440900	-3.89152000
H	-6.32801200	7.32829200	-2.96920400
H	-6.56556600	7.53417900	-4.70985100
H	-4.99034300	7.02589000	-4.08924500
C	-2.10375700	8.43744600	-3.86377900
H	-1.64272100	9.41228300	-4.04786500
H	-1.29604200	7.69628700	-3.84100300
H	-2.74543300	8.20285600	-4.72060300
C	-4.69952800	12.36425500	-2.79637900
H	-5.48988300	12.20684700	-3.53830900
H	-5.16379100	12.44694000	-1.80780300
H	-4.22673200	13.32420300	-3.02572300
C	-2.80581700	3.90686100	-6.47147500
H	-3.62388600	3.19025700	-6.60558100
H	-2.54164700	3.88820900	-5.40621100
C	-0.08011200	4.60114900	-7.38164000
H	-0.49087500	5.42155800	-7.98179500
H	0.77138200	4.21015300	-7.94662200
C	-0.67839100	1.91899800	-6.30357000
H	0.30374200	1.65558500	-6.70902000

H	-0.52594900	2.34383100	-5.30388200
C	-3.26626000	5.31511800	-6.87386900
H	-3.51679400	5.37650600	-7.93584400
H	-4.16378700	5.59173500	-6.30918900
H	-2.49929500	6.06712200	-6.66521100
C	0.36259500	5.10604100	-6.00235200
H	1.08691900	5.91844100	-6.12580400
H	-0.47251900	5.49978700	-5.41209300
H	0.84985700	4.31870600	-5.41814500
C	-1.56961900	0.67229100	-6.23099400
H	-1.11766200	-0.07921700	-5.57422300
H	-2.56567600	0.90031900	-5.83353300
H	-1.68977800	0.22391700	-7.22275400
C	-0.27696300	2.50822600	-12.91067800
H	0.69553600	2.12096000	-12.58745600
H	-0.41912000	2.19499100	-13.95282100
C	-0.91140600	-0.14926600	-11.91224200
H	-0.77473000	-0.43812800	-12.96204900
H	0.07861600	-0.12741300	-11.44484700
C	-0.28936200	4.03788300	-12.78537100
H	-1.23550400	4.47736400	-13.11295900
H	-0.10695200	4.34392300	-11.75035300
H	0.50321900	4.46211100	-13.41010700
C	-3.09554800	1.52357300	-12.88077000
H	-3.85854800	1.06842900	-12.23917400
H	-2.90572400	0.81347900	-13.69618800
C	-1.81902600	-1.15089000	-11.18823900
H	-1.94606800	-0.87222300	-10.13509700
H	-2.80922700	-1.21904600	-11.65179600
H	-1.37455300	-2.15094000	-11.22176300
C	-3.60549400	2.85136000	-13.45747600
H	-4.56121900	2.68794600	-13.96567900
H	-3.76476200	3.60413200	-12.67954800
H	-2.91380900	3.26402500	-14.19686700
C	-4.88768700	-11.30899500	-5.48415300
H	-4.68576800	-11.71859400	-6.48240200
H	-3.92736200	-11.13675700	-4.99018800
C	-7.21706200	-10.04574000	-6.68306900
H	-7.96629500	-10.37977000	-5.95745900
H	-6.97438400	-10.91822400	-7.30291700
C	-7.69978200	-6.83289600	-0.87793500
H	-7.82922100	-6.91660700	0.20843600
H	-8.57167900	-7.31085100	-1.33574400
C	-6.36241400	-9.37608000	-0.30731300
H	-6.41719300	-9.07821300	0.74666500
H	-5.42395800	-9.92337600	-0.44940600
C	-4.76039900	-6.91782800	-0.66949400
H	-4.69690700	-5.98505500	-1.24302900
H	-3.88319900	-7.50850200	-0.94945100
C	-4.77167000	-6.62945700	0.83769800
H	-3.88517500	-6.04519600	1.10987700
H	-5.65242200	-6.05501100	1.14661100
H	-4.74739300	-7.55117500	1.42672200
C	-7.55744100	-10.26833400	-0.67380100
H	-7.50186000	-10.59426500	-1.71778100

H	-7.56118200	-11.16371100	-0.04439700
H	-8.51535500	-9.75895700	-0.52116400
C	-7.62995100	-5.35828200	-1.29506400
H	-7.45643500	-5.25040500	-2.36999000
H	-8.57434300	-4.85482200	-1.06062700
H	-6.83043800	-4.82449500	-0.77021900
C	-4.57901400	-8.78319400	-6.95703100
H	-4.59452900	-9.38124600	-7.87766600
H	-5.06196700	-7.82771500	-7.19422200
C	-3.13516300	-8.54962000	-6.49394200
H	-2.58772900	-7.99248300	-7.26193400
H	-3.09942200	-7.98312300	-5.55857400
H	-2.60542100	-9.49279600	-6.33158100
C	-5.73678900	-12.28514500	-4.65829600
H	-5.92478300	-11.89119900	-3.65409900
H	-6.69937700	-12.50171900	-5.13240400
H	-5.20608300	-13.23634400	-4.55187100
C	-7.79016900	-8.93270300	-7.56995000
H	-8.04485200	-8.03725700	-6.99533900
H	-7.09363000	-8.64520600	-8.36349700
H	-8.70460000	-9.28908900	-8.05480200
C	0.25357800	-4.38026500	7.17365000
H	0.14227100	-4.61130200	6.10794700
H	-0.71523500	-4.02372600	7.53773200
C	2.99271600	-3.46134400	6.53417200
H	3.65190200	-2.58602200	6.58213300
H	3.44381900	-4.21269300	7.19190100
C	0.70387000	-1.55350700	6.34566900
H	1.54720400	-0.88976000	6.11299600
H	0.05802900	-1.00904400	7.04051300
C	-0.08453800	-1.90432300	5.07569300
H	-0.42848900	-0.98182800	4.59406000
H	0.51028600	-2.45789500	4.34294300
H	-0.97253300	-2.49881900	5.31085500
C	0.68564500	-5.62561000	7.96036900
H	0.75947700	-5.40786700	9.03053500
H	-0.05213500	-6.42436700	7.83265800
H	1.65315200	-6.01500500	7.62293500
C	2.91949600	-3.99223900	5.09612300
H	2.57888800	-3.22566800	4.39381900
H	3.91356000	-4.32021800	4.76942600
H	2.24679600	-4.85204100	5.01140000
C	1.25357200	-0.01419500	12.40916100
H	1.35781800	0.07333300	13.49868300
H	2.08723600	0.54281900	11.96614300
C	0.13329200	-2.71296600	12.80793000
H	0.07195700	-2.35640400	13.84421900
H	-0.78315400	-2.40652000	12.29524000
C	3.00928500	-2.27838600	13.01734400
H	3.32282200	-3.27632500	12.69286800
H	2.65096500	-2.38344700	14.04937200
C	-0.08555200	0.57862800	11.95355200
H	-0.12418600	1.63876600	12.22490900
H	-0.21961000	0.49035100	10.87212600
H	-0.93287000	0.08474500	12.43817500

C	0.27633600	-4.24054700	12.76742800
H	0.32587600	-4.60490900	11.73528800
H	1.16747800	-4.58726100	13.30051900
H	-0.59020700	-4.70604100	13.24722100
C	4.19553500	-1.30542400	12.98071400
H	5.01855900	-1.70832800	13.57949500
H	4.56596500	-1.14729000	11.96273900
H	3.93471800	-0.33058300	13.40302300
C	4.63119000	8.81786100	6.92923500
H	4.64818600	9.41238500	7.85212000
H	5.11184000	7.86031500	7.16276100
C	7.27310700	10.07335800	6.65869200
H	8.02271700	10.40858700	5.93399700
H	7.03349300	10.94376400	7.28264900
C	4.94686900	11.34924100	5.46762700
H	4.74774200	11.75493900	6.46802000
H	3.98523200	11.18261400	4.97434400
C	3.18675700	8.58950200	6.46535100
H	2.63774500	8.03153900	7.23160700
H	2.65952800	9.53453200	6.30563900
H	3.14971900	8.02577700	5.52833200
C	7.84375800	8.95488300	7.54023200
H	8.09656400	8.06172400	6.96131700
H	8.75896100	9.30699100	8.02675800
H	7.14658700	8.66510100	8.33240600
C	5.79806500	12.32630600	4.64498400
H	6.76216700	12.53726300	5.11854000
H	5.98303500	11.93653600	3.63860900
H	5.27046800	13.27982000	4.54397500
C	6.40996400	9.43024500	0.27975700
H	6.46272200	9.13551300	-0.77520500
H	5.47294400	9.97918300	0.42489200
C	7.74240300	6.88245000	0.84112900
H	7.87119200	6.96936700	-0.24504400
H	8.61569700	7.35713600	1.29965600
C	4.80272100	6.97436100	0.63674100
H	4.73851500	6.03974200	1.20720600
H	3.92725500	7.56590500	0.92036100
C	7.66974200	5.40670800	1.25372400
H	8.61308100	4.90210200	1.01755200
H	7.49588900	5.29594500	2.32832200
H	6.86908200	4.87622800	0.72724200
C	7.60749600	10.31868600	0.64743400
H	7.61209300	11.21611700	0.02097800
H	7.55427600	10.64125700	1.69257700
H	8.56410400	9.80787200	0.49152800
C	4.81056600	6.69109600	-0.87142700
H	5.68961800	6.11598600	-1.18391300
H	3.92237400	6.10956600	-1.14395800
H	4.78705700	7.61483400	-1.45728400
C	10.45513800	4.20868300	-4.56039600
H	10.32549600	5.28323600	-4.73876200
H	11.47419800	3.96253900	-4.87391500
C	7.59717400	3.67459700	-5.04574700
H	7.52134700	3.16218300	-4.07888700

H	6.91024300	3.15526900	-5.72070300
C	9.43178200	4.22959200	-7.29901400
H	9.18113500	5.28083300	-7.11367000
H	8.65671400	3.81137000	-7.95006700
C	10.80829300	4.12317700	-7.97207600
H	11.06706500	3.08092400	-8.18728900
H	10.79982100	4.66724200	-8.92157900
H	11.60306600	4.55869900	-7.35698500
C	10.26996000	3.88694000	-3.07254200
H	10.33504400	2.80971100	-2.88864600
H	11.04822700	4.38071900	-2.48086600
H	9.29980300	4.23278100	-2.69987100
C	7.22141400	5.15617300	-4.90753100
H	6.21180500	5.24512700	-4.49003200
H	7.90199600	5.70027300	-4.24343800
H	7.21844900	5.66804300	-5.87437300
C	9.14950200	-2.60471900	-6.31911000
H	9.47190300	-3.51622400	-6.83948300
H	9.49684100	-2.69080500	-5.28290500
C	9.58861700	-1.21948600	-8.86842100
H	9.72127600	-2.25271800	-9.21529700
H	8.52208700	-0.98248800	-8.91503500
C	11.84529700	-1.75261600	-7.11978700
H	12.45647000	-0.87517300	-7.35564400
H	11.92979600	-2.43005400	-7.97890300
C	7.62121600	-2.47641400	-6.35649900
H	7.16407800	-3.33465400	-5.85107600
H	7.28585800	-1.56035700	-5.86107500
H	7.24296500	-2.45772200	-7.38296300
C	12.38201100	-2.45096300	-5.86378300
H	13.43630200	-2.70566400	-6.01235500
H	12.31430100	-1.81411100	-4.97677000
H	11.84845600	-3.38403600	-5.65684700
C	10.38076400	-0.24510900	-9.75147800
H	10.23687600	0.78910700	-9.42279900
H	11.45344100	-0.46379800	-9.75322200
H	10.03213300	-0.31920000	-10.78620000
C	5.64458700	-8.38735600	3.53053100
H	5.86092400	-7.43691600	3.02665500
H	6.57588700	-8.96444600	3.49713300
C	2.84063300	-8.37327700	2.51029900
H	2.32487900	-8.61359700	1.57610600
H	3.10374100	-7.30819500	2.45441500
C	4.20822500	-10.93199300	3.27214100
H	5.21184900	-11.35689900	3.39712500
H	3.82323500	-10.73732600	4.28008400
C	1.91796100	-8.64670000	3.70761000
H	1.01636900	-8.02906200	3.62066700
H	1.59094700	-9.69002200	3.73419800
H	2.38640400	-8.41917400	4.66929900
C	5.23820500	-8.13331700	4.98879700
H	6.06783500	-7.65796600	5.52445800
H	4.37272800	-7.46686500	5.05961600
H	4.99933000	-9.05959600	5.51988500
C	3.30311800	-11.93085700	2.53762200

H	2.29100000	-11.54052300	2.39281700
H	3.70140900	-12.18248200	1.55154700
H	3.22736400	-12.85246100	3.12339500
C	7.72717600	-10.46051600	-2.38339400
H	7.85701100	-11.17983000	-3.20118100
H	8.25867000	-10.87220200	-1.51906500
C	5.07490500	-9.58005600	-3.36438100
H	5.40709300	-8.53499700	-3.33283000
H	4.01189200	-9.57261300	-3.10297000
C	5.41092700	-12.21924600	-2.07361900
H	4.31915400	-12.22799400	-1.98398800
H	5.66372100	-12.57850900	-3.07809500
C	6.04500600	-13.12613100	-1.00928700
H	7.13623200	-13.16119500	-1.09524400
H	5.78841100	-12.79165100	0.00204200
H	5.67784600	-14.15018700	-1.12813500
C	8.32427700	-9.10398100	-2.77933300
H	7.87648700	-8.71711000	-3.70015300
H	8.18289600	-8.35375700	-1.99388900
H	9.39957500	-9.20945400	-2.95730600
C	5.29633900	-10.18038800	-4.76061100
H	4.89857100	-11.19662300	-4.83542300
H	4.77649500	-9.57292700	-5.50929400
H	6.35438000	-10.21088400	-5.04129800

Coordinate of 3 from Computational studies

N	6.78212000	-2.39727700	-8.60925900
C	5.50105900	-2.09794100	-8.79969300
C	7.50662600	-1.52850900	-9.40211700
H	4.66576500	-2.60568600	-8.34176200
C	6.63894600	-0.70568700	-10.07101900
H	8.58433300	-1.56677500	-9.44984100
H	6.81314800	0.07721300	-10.79342000
N	5.83255700	-8.81957100	4.02139000
C	4.53669500	-8.61634000	4.23799600
C	6.37821100	-9.18603100	5.23704700
H	3.81337800	-8.33661800	3.48736500
C	5.38786400	-9.20091500	6.18339600
H	7.42490600	-9.42784300	5.34075900
H	5.40779500	-9.45666500	7.23201300
C	2.86165200	-8.83543400	6.11961100
H	2.20151700	-9.28560200	5.37274800
H	2.89822000	-9.52452200	6.96737900
C	3.48632400	0.64620300	-9.50294800
C	3.80715400	1.95505100	-9.88837100
C	2.50486700	0.46960000	-8.51818600
C	3.14628700	3.04784400	-9.32940100
H	4.55549100	2.12802500	-10.65818500
C	1.83053000	1.56036900	-7.96952200
H	2.23640600	-0.53507700	-8.19748700
C	2.11357000	2.87140100	-8.38941800
H	3.40327000	4.04943200	-9.66112900
H	1.05350400	1.39190100	-7.23058100

C	2.29712200	-7.49579300	6.56222200
C	1.39871600	-6.79695000	5.74501900
C	2.56088100	-6.98535300	7.84107300
C	0.74686700	-5.65219600	6.20441200
H	1.17468700	-7.16921400	4.74719900
C	1.92048100	-5.83461900	8.29750800
H	3.24602300	-7.50745600	8.50476600
C	0.96718300	-5.16747800	7.50460700
H	0.03417400	-5.14841000	5.55921000
H	2.12849800	-5.47473500	9.30070000
N	7.30168800	8.24148400	-3.62096500
C	6.00885900	8.27472300	-3.92695200
C	7.98601500	8.31644100	-4.81943700
H	5.19564300	8.26014200	-3.21683200
C	7.08384100	8.39091500	-5.84747700
H	9.06458000	8.33215200	-4.85624800
H	7.22119000	8.48053500	-6.91453600
N	6.40496800	1.56593200	8.90561500
C	5.13135600	1.52407100	9.28153400
C	6.99686100	0.42317800	9.41095900
H	4.38895500	2.27779900	9.06632100
C	6.05817600	-0.30466800	10.09244900
H	8.04558000	0.21708700	9.26233700
H	6.12866800	-1.23454300	10.63614000
C	3.59554700	0.05492400	10.63183800
H	3.80635600	-0.23690700	11.66553300
H	3.01537200	0.98294000	10.68664500
C	4.54513100	8.57617500	-5.95557700
H	3.89618100	9.09096800	-5.23752200
H	4.72982300	9.28875200	-6.76434100
C	3.82816500	7.34703300	-6.49827000
C	2.89652200	7.53610100	-7.52897100
C	3.97438800	6.06337800	-5.96296900
C	2.09652900	6.48615600	-7.97536400
H	2.78479700	8.51681900	-7.98574500
C	3.19224100	5.00489900	-6.43064500
H	4.70473900	5.87897900	-5.18022600
C	2.21228300	5.20136400	-7.41850700
H	1.37591500	6.66523700	-8.76708300
H	3.33112400	4.01353500	-6.00742400
C	2.78095300	-1.02982300	9.93728000
C	1.72857700	-1.62099100	10.65124300
C	2.99462000	-1.43176400	8.61539400
C	0.89272900	-2.56024000	10.05132300
H	1.55779500	-1.34823400	11.69022600
C	2.17186100	-2.39255100	8.02259300
H	3.81613100	-1.00871100	8.04347800
C	1.08886400	-2.95701000	8.71686100
H	0.08587600	-2.99987000	10.62899700
H	2.36902200	-2.70382400	7.00020400
N	-6.17084100	-1.57547700	-8.80452800
C	-4.87879200	-1.53744200	-9.10986500
C	-6.72512900	-0.41681200	-9.31646000
H	-4.15584100	-2.30390000	-8.87357900
C	-5.74472300	0.31563000	-9.93203500

H	-7.77839700	-0.20158000	-9.21909800
H	-5.77818800	1.25635100	-10.46032100
N	-7.19223700	-7.96605300	3.74526500
C	-5.89120600	-8.03895300	4.00678700
C	-7.83522500	-7.99589200	4.96848700
H	-5.10376000	-8.06448500	3.26854000
C	-6.89985700	-8.08371500	5.96557000
H	-8.91160600	-7.97314300	5.04497100
H	-7.00227400	-8.14965400	7.03824900
C	-4.36388500	-8.34575500	5.98621600
H	-4.52118900	-9.09393500	6.76818400
H	-3.73329200	-8.82299700	5.22708900
C	-3.26456900	-0.07473300	-10.37071300
H	-2.67184600	-0.99552600	-10.32569100
H	-3.41606600	0.14262000	-11.43266200
C	-2.50437200	1.06596800	-9.70721800
C	-1.51928400	1.72667400	-10.45439000
C	-2.68581300	1.43843300	-8.37096800
C	-0.70876200	2.69948200	-9.87214900
H	-1.37725400	1.47853400	-11.50372400
C	-1.89282100	2.43410000	-7.79649500
H	-3.45636200	0.95911200	-7.77281500
C	-0.86761600	3.06251800	-8.52391700
H	0.05039700	3.18920900	-10.47370300
H	-2.06172800	2.71862800	-6.76118500
C	-3.64318000	-7.13455100	6.56139300
C	-2.76527400	-7.33629300	7.63506400
C	-3.72898400	-5.85322600	6.00552300
C	-1.95682100	-6.30240300	8.10582600
H	-2.70032800	-8.31434800	8.10620600
C	-2.94131200	-4.81090900	6.49709000
H	-4.41529400	-5.65981700	5.18578000
C	-2.01250400	-5.02219300	7.53091600
H	-1.27683900	-6.49159000	8.93047200
H	-3.03215100	-3.82132600	6.05682000
N	-5.82363700	8.84554500	-4.03385500
C	-4.52564900	8.59456400	-4.16827100
C	-6.25063800	9.35876300	-5.24379200
H	-3.88408000	8.19453700	-3.39817200
C	-5.18518700	9.41579800	-6.10299100
H	-7.27450200	9.66056500	-5.40312700
H	-5.10610900	9.77619900	-7.11759600
N	-6.70583300	2.38932600	8.53287800
C	-5.40904200	2.16398700	8.71944200
C	-7.37527900	1.55407300	9.40660100
H	-4.60510500	2.66883400	8.20565000
C	-6.45874700	0.82667700	10.11941600
H	-8.45262600	1.54658100	9.47243800
H	-6.58351800	0.09745300	10.90544500
C	-3.90593500	0.77602800	10.20293300
H	-3.23145200	1.63321700	10.12736800
H	-4.05816900	0.58383800	11.26862800
C	-2.69278900	8.91502400	-5.88026600
H	-2.64850300	9.63775600	-6.69948300
H	-2.08171100	9.31915700	-5.06828800

C	-2.12541700	7.58013500	-6.33384400
C	-2.42989400	7.04789800	-7.59510200
C	-1.17986900	6.91082800	-5.54616600
C	-1.77920600	5.90883700	-8.06531800
H	-3.15563100	7.54424100	-8.23501700
C	-0.51772700	5.77769500	-6.02039200
H	-0.92705100	7.29871500	-4.56133700
C	-0.77548100	5.27521500	-7.30648800
H	-2.01784500	5.53336000	-9.05592300
H	0.23201800	5.29860900	-5.39887900
C	-3.27919800	-0.43708900	9.53550300
C	-2.24526600	-0.28264400	8.60245700
C	-3.64207700	-1.73853300	9.90975000
C	-1.56464200	-1.38800200	8.09089400
H	-1.94239000	0.71563900	8.29290100
C	-2.97542700	-2.84497300	9.38675500
H	-4.43143900	-1.89552000	10.64102200
C	-1.89542300	-2.69201900	8.49612800
H	-0.74917600	-1.23648000	7.39077800
H	-3.26779300	-3.84036600	9.70753100
C	0.22013400	-3.99173300	8.06301700
C	-1.14388800	-3.89912500	8.02018400
C	-0.01357800	4.12064900	-7.88708400
C	1.35326300	4.06102300	-7.88309300
N	-5.66623300	-8.11000300	5.34364300
N	-5.21306200	1.22055200	9.67298200
N	4.22218600	-8.83590000	5.53815400
N	4.87494900	0.40296800	10.00231800
N	-4.57390100	-0.40437600	-9.79203300
N	-4.09480000	8.92539000	-5.41041400
N	5.82862400	8.36306800	-5.26989200
N	5.36741900	-1.07479700	-9.67935800
C	4.09316500	-0.55019300	-10.21706600
H	3.38771800	-1.38566500	-10.20758100
H	4.28173800	-0.30521600	-11.26572100
P	8.90913100	5.84678600	-1.89973800
P	7.81347800	10.41306900	-1.13996300
P	-6.75942900	10.70569200	-1.39316600
P	-7.70505600	6.34527200	-3.06472300
P	8.16152200	-2.39618100	-5.58200600
P	7.35122100	-5.67567900	-8.93871200
P	-6.65639500	-1.98874800	-5.55688700
P	-7.94854400	-4.33636800	-9.50426300
Pt	8.09462100	8.07782200	-1.60898500
Pt	-6.99693300	8.48719200	-2.25926500
Pt	7.53068100	-3.93789600	-7.29364500
Pt	-7.17648200	-3.07631400	-7.61708700
C	8.68176500	7.83930900	0.25538700
C	9.01676800	7.63098000	1.41556000
C	-7.98987000	8.11624400	-0.60083800
C	-8.57028900	7.86521900	0.44871700
C	8.13913400	-5.30773700	-6.01502800
C	8.51403700	-6.11585200	-5.17370200
C	-8.07125300	-4.38759500	-6.45022300
C	-8.61457900	-5.14705300	-5.65587500

C	10.99611800	7.94440000	4.57389200
C	9.45335700	7.42358900	2.76617400
C	10.35760800	7.02426400	5.40162600
H	11.84629800	8.50661200	4.94838100
C	8.81169400	6.49781200	3.61079800
C	9.25394800	6.28358900	4.93049900
H	10.71187500	6.86651800	6.41521400
H	7.95743300	5.94145800	3.23635700
C	-9.27850800	7.58589600	1.66393600
C	-11.25925900	7.84746500	3.05058000
C	-8.71055900	6.77571200	2.66516500
C	-10.69231600	7.04353900	4.03702300
H	-12.24993100	8.26600900	3.20130100
C	-9.40568400	6.49514000	3.85658200
H	-7.71477300	6.36795400	2.51923100
H	-11.23850200	6.83268000	4.95098500
C	8.99145600	-7.07306800	-4.21857200
C	10.59918500	-8.78583500	-3.58733300
C	8.35828300	-7.23826700	-2.97222500
C	9.96908200	-8.94275300	-2.35495700
H	11.47048800	-9.38816200	-3.82676100
C	8.83684400	-8.16752600	-2.02942400
H	7.48007300	-6.64451500	-2.73655300
H	10.34675300	-9.66180700	-1.63512100
C	-11.14304600	-7.56513300	-4.32940500
C	-9.29628700	-6.04233300	-4.76644100
C	-10.67673100	-7.78183000	-3.03483500
H	-12.04295200	-8.06932700	-4.66869700
C	-8.83016400	-6.26892600	-3.45710500
C	-9.50987200	-7.13409900	-2.57883600
H	-11.21188000	-8.44816700	-2.36584400
H	-7.92509300	-5.77282200	-3.11902000
C	-8.82685900	5.66321500	4.87102300
C	-8.35392500	4.95872300	5.75485600
C	8.62072600	5.31920500	5.78372800
C	8.12863200	4.48792700	6.53856400
C	8.19944100	-8.32796000	-0.75476200
C	7.68334000	-8.49342000	0.34401200
C	-9.04384800	-7.35324300	-1.24013500
C	-8.68055800	-7.56427000	-0.08912900
Pt	7.32735100	3.09529000	7.68345500
Pt	-7.58353600	3.75652700	7.11272300
Pt	-8.04220200	-7.80101700	1.75831600
Pt	6.83204300	-8.68272700	2.10980600
P	-8.02225800	2.04103300	5.50803600
P	-7.56747300	5.62934500	8.61080200
P	8.11129600	4.40326300	9.52130700
P	6.68843500	2.09726200	5.60858300
P	7.89124600	-6.60081800	2.60368600
P	6.22854200	-10.91701900	1.49681200
P	-7.86820900	-10.15230600	1.31696800
P	-8.73271700	-5.52710100	2.02800000
C	-9.19276300	-10.75420700	0.17171100
H	-9.07238800	-10.21491500	-0.77133500
H	-8.98275900	-11.81493900	-0.01836000

C	-8.08132500	-11.26595400	2.79778500
H	-8.86085400	-10.81788700	3.42324900
H	-8.49532300	-12.20504900	2.41020800
C	-6.28077300	-10.69981200	0.52717600
H	-6.32060600	-11.79305500	0.43576500
H	-5.47445000	-10.47109100	1.23430100
C	-5.99143300	-10.05738000	-0.83643000
H	-5.97951300	-8.96471600	-0.77196000
H	-5.01717000	-10.39583400	-1.20512100
H	-6.73999400	-10.33837300	-1.58270800
C	-10.62049600	-10.57137500	0.70466600
H	-10.78557700	-11.10602700	1.64565100
H	-10.84939700	-9.51238400	0.86041600
H	-11.33710300	-10.96791500	-0.02156200
C	-6.83251100	-11.57042800	3.63473200
H	-6.38322300	-10.66518000	4.05398800
H	-7.10640600	-12.22297500	4.47001200
H	-6.06948000	-12.09451000	3.05125700
C	-10.58016800	-5.45047600	1.90053000
H	-10.89418100	-4.40472900	2.00333800
H	-10.82105900	-5.77127700	0.88113800
C	-8.35669800	-4.67315300	3.63733200
H	-8.99010300	-3.77937600	3.69994400
H	-8.68087500	-5.34259300	4.44028600
C	-8.09592400	-4.37689900	0.71741100
H	-7.00259300	-4.38115800	0.80452500
H	-8.32901500	-4.87360400	-0.22938300
C	-11.31475800	-6.33334900	2.92000000
H	-12.39584100	-6.25512200	2.76877800
H	-11.03635000	-7.38618300	2.80347800
H	-11.10850900	-6.03138500	3.95301000
C	-6.88161300	-4.29963400	3.82767600
H	-6.71784900	-3.89530700	4.83293200
H	-6.23257400	-5.17305500	3.70722300
H	-6.55846200	-3.54090000	3.10673700
C	-8.63935800	-2.94219700	0.73591700
H	-8.42181800	-2.42471300	1.67742200
H	-8.17872100	-2.36031600	-0.07113000
H	-9.72184600	-2.91442800	0.57909900
C	4.79086900	-11.61823500	2.44526100
H	3.96339200	-10.90725700	2.33224700
H	5.08148300	-11.59275500	3.50206000
C	5.81291600	-11.14756100	-0.28903500
H	6.68722600	-10.80793000	-0.85094200
H	5.69804300	-12.22200300	-0.47424100
C	7.62080200	-12.09207000	1.84749400
H	7.79417900	-12.04797600	2.92980200
H	7.25994900	-13.10349600	1.62631300
C	8.92536200	-11.81850300	1.08682900
H	8.79487700	-11.92598000	0.00552900
H	9.30366300	-10.81134300	1.28315500
H	9.68607400	-12.54053700	1.39966600
C	4.56846100	-10.37366000	-0.74154800
H	3.66441700	-10.70292900	-0.21651200
H	4.69660900	-9.29877900	-0.57482200

H	4.40155200	-10.53203100	-1.81195200
C	4.33517200	-13.03226800	2.05233200
H	5.12886300	-13.77283900	2.18507900
H	3.49746600	-13.34067000	2.68696300
H	3.99557100	-13.08112500	1.01325900
C	9.72779900	-6.84482200	2.62792800
H	9.99909100	-7.15063200	1.61156500
H	10.20577900	-5.87754300	2.82400600
C	7.51746700	-5.79480500	4.23758700
H	8.29086400	-5.03909500	4.42344700
H	7.64036000	-6.56360800	5.00728300
C	7.57963800	-5.28963200	1.32841700
H	6.49854600	-5.10395200	1.33198100
H	7.80320200	-5.77117500	0.37114800
C	6.12058700	-5.16922600	4.33872100
H	5.33620300	-5.90112300	4.12175100
H	5.94985300	-4.78856500	5.35200200
H	5.99993700	-4.33072500	3.64399900
C	8.35955200	-3.97752800	1.48433700
H	9.43971000	-4.13375200	1.40742400
H	8.07525000	-3.27911100	0.68832500
H	8.15848000	-3.48425400	2.44218500
C	10.20624400	-7.89447100	3.64150200
H	9.97249900	-7.60982200	4.67339100
H	9.75436700	-8.87162700	3.43994500
H	11.29257400	-8.00861900	3.57420000
C	5.78482700	0.46767500	5.60178300
H	4.95851200	0.55756500	6.31624900
H	5.33005300	0.34512400	4.61031900
C	8.15547800	1.84063500	4.50262000
H	8.82513300	1.15283400	5.03200000
H	8.66999100	2.80627000	4.48749100
C	5.55390100	3.22666200	4.67171800
H	6.13349400	4.13929700	4.49764700
H	5.32750600	2.77798400	3.69670300
C	6.65017800	-0.75345200	5.94312400
H	7.11704400	-0.65631300	6.92680500
H	6.03537500	-1.66091500	5.95626700
H	7.44527600	-0.90581900	5.20635000
C	7.87687700	1.33914200	3.08043200
H	8.82328000	1.22405300	2.54050100
H	7.37388900	0.36559300	3.07096800
H	7.25838000	2.04417000	2.51456900
C	4.26161400	3.56537900	5.42630300
H	3.65543100	4.26742900	4.84371700
H	3.64819400	2.67595800	5.61340800
H	4.48847900	4.03734700	6.38795100
C	9.91582400	4.81594700	9.41820500
H	9.98471700	5.63207600	8.69121700
H	10.20933000	5.22815200	10.39195500
C	7.28928400	6.06437000	9.51881400
H	7.76979300	6.66949000	10.29807400
H	7.53155400	6.51887900	8.55254600
C	10.84563500	3.66990500	8.99762400
H	10.80912500	2.82650900	9.69280100

H	10.58486900	3.30665700	7.99885200
H	11.87978500	4.02809400	8.97004900
C	7.80561700	3.76760400	11.24246500
H	6.77151500	3.40640800	11.27116100
H	7.85052900	4.64438600	11.90140000
C	5.77061400	6.02456300	9.72570900
H	5.28245400	5.43600300	8.93969600
H	5.49855800	5.59967300	10.69804900
H	5.36235800	7.03959600	9.68865100
C	8.76678000	2.68755200	11.75768300
H	8.45877300	2.37037800	12.75921500
H	8.77702100	1.80155700	11.11562400
H	9.79071500	3.06235700	11.83892600
C	-9.18027400	6.53601400	8.55476800
H	-9.07518300	7.41845400	9.19901000
H	-9.30993700	6.88507100	7.52678100
C	-7.36804700	5.23948600	10.42208400
H	-7.96489800	4.34517900	10.62939500
H	-7.84353900	6.06545700	10.96603600
C	-7.42749700	0.31541700	5.87493300
H	-7.98404600	-0.37540300	5.22879700
H	-7.72375500	0.09046900	6.90478300
C	-9.85479400	1.85216100	5.30545100
H	-10.04655900	1.10536400	4.52551200
H	-10.21039100	2.81933800	4.93411900
C	-7.37479500	2.42937700	3.81214100
H	-6.28427900	2.50055800	3.90374200
H	-7.72873000	3.44341200	3.60254000
C	-7.76523300	1.46366100	2.68583600
H	-7.29881100	1.78168300	1.74602300
H	-7.43665300	0.43683900	2.88455600
H	-8.84704400	1.44630800	2.52281300
C	-10.59236800	1.47737100	6.59893000
H	-10.43554300	2.23326400	7.37542300
H	-11.66847300	1.41355800	6.40981700
H	-10.27076400	0.50626800	6.99192200
C	-5.91749300	0.10662500	5.70322100
H	-5.34235500	0.81166400	6.31117300
H	-5.63610500	-0.90509900	6.01601700
H	-5.60618700	0.23042600	4.66041100
C	-6.27220800	6.92006500	8.28680000
H	-6.34030500	7.64438300	9.10932200
H	-5.29967900	6.42334200	8.38357300
C	-6.36736600	7.63909700	6.93531800
H	-5.54150000	8.35299700	6.84545900
H	-6.31526900	6.93643700	6.09938100
H	-7.30030000	8.20210200	6.84011600
C	-10.38863100	5.69115300	8.98149600
H	-10.51237800	4.82329200	8.32482700
H	-10.30377900	5.33643900	10.01384800
H	-11.30091000	6.29250500	8.91929600
C	-5.93151500	5.05396400	10.92849800
H	-5.40581700	4.25631300	10.39357300
H	-5.34482800	5.97232400	10.83179800
H	-5.94910700	4.79034100	11.99094500

C	-8.19460600	-1.50808800	-4.64509400
H	-7.91964900	-1.09354500	-3.66842500
H	-8.72931200	-2.44751100	-4.46913000
C	-5.65730200	-0.42691200	-5.72628100
H	-4.72117000	-0.72262600	-6.21612900
H	-6.19229600	0.19586300	-6.45213900
C	-5.69084500	-3.11110600	-4.42661900
H	-4.64281700	-3.03640400	-4.74522700
H	-6.02775100	-4.11835200	-4.68812300
C	-5.81768600	-2.90384500	-2.91030900
H	-5.19029800	-3.63628500	-2.38926800
H	-5.50255100	-1.90700700	-2.58651800
H	-6.84812100	-3.05898000	-2.57489000
C	-9.08970800	-0.53210900	-5.42089100
H	-9.39703400	-0.95957400	-6.38040200
H	-9.99724500	-0.31881500	-4.84691300
H	-8.58915200	0.42343200	-5.61596000
C	-5.35764900	0.37874500	-4.45453000
H	-4.73590400	-0.18446900	-3.75213300
H	-4.81618500	1.29710100	-4.71115600
H	-6.27416200	0.67414500	-3.93317300
C	-7.07629100	-5.95049700	-9.78449600
H	-7.44744800	-6.34095300	-10.74139900
H	-6.01555000	-5.71625100	-9.92955600
C	-9.73977300	-4.76869400	-9.33719400
H	-9.99749000	-5.41657700	-10.18476200
H	-9.83257600	-5.36297700	-8.42381300
C	-7.86256900	-3.48863400	-11.16021200
H	-8.21745500	-2.46176600	-11.02228600
H	-8.60069800	-3.99104900	-11.79855000
C	-7.23972900	-7.00428600	-8.68179100
H	-6.66655200	-7.89822600	-8.94856400
H	-6.88371400	-6.63780900	-7.71536000
H	-8.28371100	-7.30852700	-8.56243200
C	-10.67646100	-3.55485200	-9.27400600
H	-10.42959000	-2.91174800	-8.42209100
H	-10.63691100	-2.95304800	-10.18778300
H	-11.71080400	-3.89121700	-9.15246400
C	-6.49478100	-3.49261900	-11.85616200
H	-6.56160500	-2.93827300	-12.79786900
H	-5.71927500	-3.02182800	-11.24317100
H	-6.16612200	-4.50669600	-12.10062600
C	5.85548300	-6.76646300	-8.80027400
H	5.86623400	-7.43690800	-9.66965200
H	4.97819700	-6.11829000	-8.91117400
C	7.35894900	-5.11805900	-10.71786800
H	8.07629000	-4.29414200	-10.79362200
H	7.77797900	-5.95109400	-11.29619600
C	8.79392300	-6.83294700	-8.86307200
H	8.59699400	-7.63587200	-9.58531500
H	8.79290700	-7.27519600	-7.86313900
C	5.75715200	-7.57736200	-7.50148800
H	4.82113100	-8.14605600	-7.49505000
H	6.57759200	-8.29490600	-7.41055500
H	5.78023200	-6.93122800	-6.61931700

C	6.00880800	-4.70838200	-11.32061900
H	5.54292600	-3.88507300	-10.76976600
H	6.15581400	-4.37860200	-12.35416200
H	5.30384500	-5.54473500	-11.34412000
C	10.14880500	-6.16937800	-9.14565600
H	10.19689500	-5.73276900	-10.14855700
H	10.36316500	-5.38353800	-8.41384000
H	10.94615300	-6.91630800	-9.08008800
C	9.99870600	-2.47892800	-5.35508600
H	10.27992200	-1.81690700	-4.52734100
H	10.21059600	-3.50858400	-5.04774100
C	7.82425500	-0.58618500	-5.85224200
H	8.46153200	-0.02229700	-5.15932800
H	8.16996400	-0.34526300	-6.86263400
C	7.43414700	-2.78232400	-3.91854600
H	6.34627200	-2.69228800	-4.02583000
H	7.63848400	-3.84614700	-3.76353400
C	6.35641700	-0.17083400	-5.68745200
H	6.23506200	0.89196700	-5.92473000
H	5.70083900	-0.73590400	-6.35713800
H	6.00279100	-0.32456300	-4.66218800
C	10.79840000	-2.12816500	-6.61815600
H	11.86934000	-2.24677100	-6.42640000
H	10.53298900	-2.79005400	-7.44933100
H	10.63640300	-1.09209300	-6.93545900
C	7.93376900	-1.94541200	-2.73349200
H	7.74739100	-0.87413200	-2.87096600
H	7.41520400	-2.25361700	-1.81776700
H	9.00582700	-2.08182800	-2.56251600
C	8.63273200	5.00476100	-3.53476300
H	9.30972600	4.14296000	-3.58698900
H	8.95399900	5.70116200	-4.31557100
C	8.29788200	4.63624600	-0.63196600
H	7.20920100	4.58057600	-0.75372600
H	8.47355300	5.12435200	0.33166500
C	10.75223500	5.87404300	-1.71073200
H	11.12831100	4.84739400	-1.79400500
H	10.93726600	6.21446800	-0.68605500
C	11.47048500	6.79062600	-2.71184100
H	11.11705100	7.82386900	-2.62627000
H	12.54648400	6.78836000	-2.51220600
H	11.33177800	6.46014900	-3.74725400
C	7.18500900	4.56504200	-3.78463800
H	6.49072600	5.40484100	-3.67720500
H	7.07854900	4.16737500	-4.80008900
H	6.87267200	3.78143200	-3.08588200
C	8.92292900	3.23538100	-0.66480900
H	8.47239000	2.60892600	0.11367300
H	8.76565000	2.72988400	-1.62449900
H	9.99972600	3.26591900	-0.47354600
C	6.19388900	10.89024300	-0.36996700
H	6.19088300	11.98297700	-0.26512200
H	5.40835200	10.63998600	-1.09301500
C	9.09555200	11.05187100	0.03360300
H	8.84187600	12.10130300	0.23279300

H	8.98158900	10.49687000	0.96825100
C	8.00747700	11.55236700	-2.60446200
H	8.80622700	11.13622700	-3.22773200
H	8.38835800	12.49895900	-2.20151900
C	5.90676900	10.22070800	0.98105100
H	4.91608400	10.52110600	1.33883200
H	5.93338000	9.12921500	0.90303300
H	6.63429300	10.51731200	1.74199900
C	6.75790300	11.82875700	-3.45011500
H	7.01982500	12.49743000	-4.27644100
H	6.33895100	10.91489600	-3.88213600
H	5.97440900	12.32401400	-2.86859900
C	10.53720100	10.93062000	-0.47901000
H	10.80788700	9.88335000	-0.64613800
H	10.69615500	11.48460400	-1.40980400
H	11.22745000	11.34309700	0.26369000
C	-6.66280000	5.70341400	-4.46903900
H	-5.63043400	5.68193200	-4.09794400
H	-6.69173500	6.48462300	-5.23713500
C	-7.70372900	5.01603600	-1.76235500
H	-7.90435300	5.54020400	-0.82353100
H	-6.66688400	4.65776100	-1.70352300
C	-9.41647500	6.42705400	-3.78148400
H	-9.32923900	7.03139300	-4.69309800
H	-9.69860900	5.41705300	-4.10138800
C	-8.68116500	3.84335800	-1.92991000
H	-8.56131400	3.14613500	-1.09226700
H	-9.72104800	4.18231400	-1.91945400
H	-8.52110600	3.28161000	-2.85505400
C	-7.02923300	4.34803400	-5.08913900
H	-6.37303300	4.14471300	-5.94370900
H	-6.90688500	3.52761400	-4.37492900
H	-8.05937900	4.32823400	-5.45810300
C	-10.49440900	7.01848500	-2.86236400
H	-10.60851400	6.44530500	-1.93741400
H	-10.25800500	8.04773300	-2.58193800
H	-11.45814400	7.01584000	-3.38153500
C	-5.68388600	11.88828200	-2.34044800
H	-5.96421300	12.90243500	-2.03055400
H	-5.95751000	11.79973400	-3.39695800
C	-6.15252300	10.76442900	0.35750600
H	-5.14443900	10.33198900	0.35164800
H	-6.78459800	10.06018300	0.90710400
C	-8.42584600	11.51202000	-1.37754500
H	-9.04527900	10.90902500	-0.70490400
H	-8.31452800	12.50328700	-0.92360400
C	-9.08724600	11.62938400	-2.75792000
H	-8.50394300	12.25477700	-3.44211900
H	-9.22458800	10.64497300	-3.21972000
H	-10.07363400	12.09275200	-2.65785800
C	-4.17485400	11.68095400	-2.15771800
H	-3.85951800	11.88186200	-1.12930100
H	-3.87311000	10.65785700	-2.40794000
H	-3.62243900	12.36675800	-2.80855900
C	-6.14971300	12.14639100	1.02839400

H	-7.15750400	12.56403300	1.10958500
H	-5.75250600	12.05913500	2.04519700
H	-5.52551100	12.87080900	0.49504800
C	-10.46573700	-6.70615700	-5.19148700
H	-10.83880400	-6.53823500	-6.19665400
C	-10.56650600	8.12058900	1.87315800
H	-11.01401700	8.74731400	1.10834900
C	10.55542700	8.14661600	3.26816200
H	11.06182100	8.85923500	2.62501700
C	10.12204200	-7.86239200	-4.51469800
H	10.62098100	-7.74097200	-5.47089900
